# Same stimulus, same temporal context, different percept? Individual differences in hysteresis and adaptation when perceiving multistable dot lattices

**DOI:** 10.1177/20416695221109300

**Published:** 2022-07-06

**Authors:** Eline Van Geert, Pieter Moors, Julia Haaf, Johan Wagemans

**Affiliations:** Laboratory of Experimental Psychology, Department of Brain and Cognition, KU Leuven, Belgium; Laboratory of Experimental Psychology, Department of Brain and Cognition, KU Leuven, Belgium; Psychological Methods Group, 1234 University of Amsterdam, The Netherlands; Laboratory of Experimental Psychology, Department of Brain and Cognition, KU Leuven, Belgium

**Keywords:** attraction, repulsion, individual differences, perceptual organization, serial dependencies, context effects

## Abstract

How we perceptually organize a visual stimulus depends not only on the stimulus itself, but also on the temporal and spatial context in which the stimulus is presented and on the individual processing the stimulus and context. Earlier research found both attractive and repulsive context effects in perception: tendencies to organize visual input similarly to preceding context stimuli (i.e., hysteresis, attraction) co-exist with tendencies that repel the current percept from the organization that is most dominant in these contextual stimuli (i.e., adaptation, repulsion). These processes have been studied mostly on a group level (e.g., Schwiedrzik et al., 2014). Using a Bayesian hierarchical model comparison approach, the present study (*N* = 75) investigated whether consistent individual differences exist in these attractive and repulsive temporal context effects, with multistable dot lattices as stimuli. In addition, the temporal stability of these individual differences in context effects was investigated, and it was studied how the strength of these effects related to the strength of individual biases for absolute orientations. The results demonstrate that large individual differences in the size of attractive and repulsive context effects exist. Furthermore, these individual differences are highly consistent across timepoints (one to two weeks apart). Although *almost everyone* showed both effects in the expected direction, *not every* single individual did. In sum, the study reveals differences in how individuals combine previous input and experience with current input in their perception, and more generally, this teaches us that different individuals can perceive identical stimuli differently, even within a similar context.

When we visually experience the world, our experience consists of organized wholes rather than many separate sensations (Wagemans, 2018). Perceptual organization of the visual input we receive from the world is an active process, including perceptual grouping and figure-ground segregation. Although the Gestalt principles of perceptual organization are often described as “laws,” which seems to imply a deterministic character, individual differences exist in sensitivity to several grouping principles such as grouping by proximity and grouping by similarity (Wagemans et al., 2018). Furthermore, when individuals perceive multistable stimuli, individual biases can exist, for instance, in the probability to perceive one orientation more often than another objectively equiprobable orientation (Kubovy & van den Berg, 2002).

Perceptual organization of current visual input can however also be influenced by its temporal context, including previously presented stimuli and their perceived organization. Earlier research has found both attractive and repulsive context effects in perception (Snyder et al., 2015). Attractive context effects (also called hysteresis, stabilization, facilitation, etc.) entail that individuals tend to organize current visual input in a similar way as preceding or simultaneous context stimuli (see the left side of Figure 1): When people perceive a certain organization in the context stimulus, they are more likely to perceive the same organization in the test stimulus. The repulsive context effect (also known as negative hysteresis, adaptation, contrast, differentiation, etc.) entails that perception tends to repel or move away from the organization that is dominant in the contextual stimuli (see the right side of Figure 1): When a lot of evidence for a certain organization is present in the context stimulus, people are less likely to perceive that organization in the test stimulus. Attractive and repulsive tendencies are concurrently present. Schwiedrzik et al. (2014) found evidence for two separate mechanisms underlying hysteresis and adaptation, as they mapped into distinct cortical networks. Whether they are part of the same process or separate processes is still under debate, however (e.g., Gepshtein & Kubovy, 2005).

**Figure 1.**
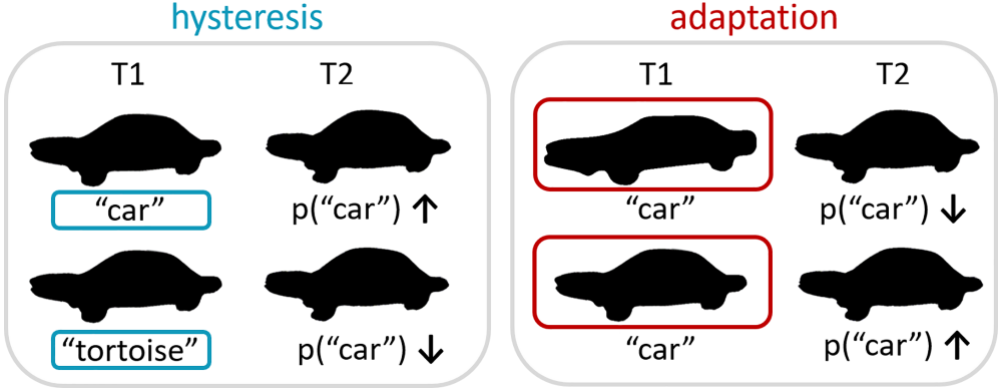
Illustration of attractive and repulsive context effects. Left side: attraction effect (hysteresis). When the stimulus is perceived as a car at time 1 (T1), the probability that another stimulus at time 2 (T2) will be perceived as a car is higher than when the stimulus at T1 was interpreted as a tortoise. Right side: repulsion effect (adaptation). When the stimulus at T1 is a very clear example of a car, the probability that another stimulus at T2 will be perceived as a car is lower than when the stimulus at T1 was a more ambiguous example of a car.

## Hysteresis and adaptation in multistable dot lattices

Gepshtein and Kubovy (2005) presented a paradigm that allows to disentangle attractive and repulsive context effects on perception. They used multistable dot lattices as context and test stimuli, and investigated the influence of (a) the perceived organization of the context stimulus (i.e., which organization was reported) and (b) the stimulus support for a certain organization in the context stimulus (dependent on the stimulus’ aspect ratio) on the perception of a second, test stimulus.

**Figure 2.**
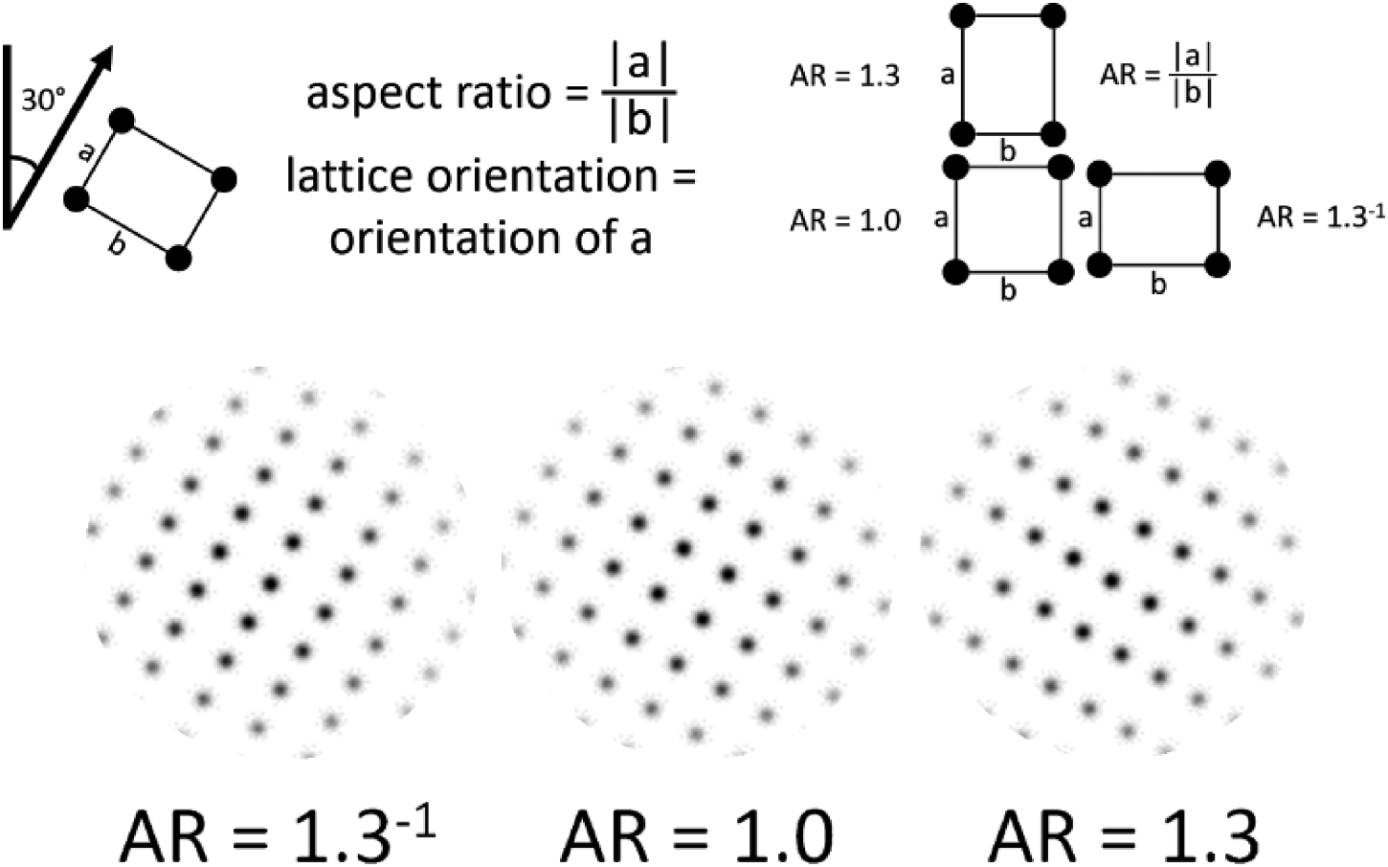
Explanation regarding the aspect ratio of a multistable rectangular dot lattice. In rectangular dot lattices, four different orientations can be perceived, of which two are more prevalent (as the dots are closer together along these orientations). The relative dominance of the *a* orientation relative to the *b* orientation is expressed in the aspect ratio of the dot lattice (AR = |*a*|/|*b*|).

Multistable dot lattices are arrays of aligned dots in which multiple orientations can be perceived (see Figure 2). The closer the dots are spaced along a particular orientation, the more likely they are grouped together and that orientation will be perceived (cf. the Gestalt law of proximity; Kubovy et al., 1998). This relative grouping strength has been shown to follow a decreasing exponential function of the relative inter-dot distance in that orientation (Kubovy et al., 1998).

**Figure 3.**
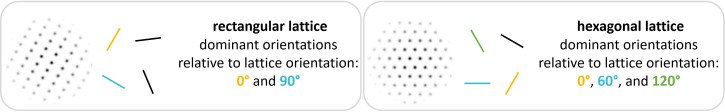
Dominantly perceived orientations in multistable rectangular and hexagonal dot lattices.

In rectangular dot lattices (see the left side of Figure 3), four different orientations can be perceived, of which two are more prevalent (as the dots are closer together along these orientations). The relative dominance of the *a* orientation relative to the *b* orientation is expressed in the aspect ratio of the dot lattice (AR = |*a*|/|*b*|).^1^ For a lattice with AR = 1, the distance between the dots in the *a* and *b* orientation is equal. For a lattice with AR < 1, the distance between the dots is smaller in the *a* than in the *b* orientation. For a lattice with AR > 1, the distance between the dots is smaller in the *b* than in the *a* orientation. In hexagonal dot lattices (see the right side of Figure 3), three prominent orientations are present and equally plausible, which makes it a very ambiguous or unstable lattice type. In both types of lattices we will define the axis orientation of the dot lattice as a whole by the orientation of *a*, which we will call the $0^{\circ }$ orientation. In the rectangular dot lattices, we will call the *b* orientation the $90^{\circ }$ orientation.

Gepshtein and Kubovy (2005) used rectangular dot lattices with a randomly varying lattice orientation as context stimuli and more ambiguous hexagonal dot lattices with the same random lattice orientation as test stimuli. The stimulus support for a particular organization in the context stimulus was manipulated by varying the aspect ratio of the rectangular lattice (i.e., the distance between the dots in the *a* vs. the *b* orientation). They then investigated the influence of (a) the perceived orientation and (b) the aspect ratio in the context stimulus on the perceived orientation in the test stimulus.

Hysteresis was present when participants perceived the same orientation in both the context and test stimulus (i.e., the a or $0^{\circ }$ orientation). Adaptation was present when participants perceived a different orientation in the test stimulus than the one for which there was most support in the context stimulus.

**Figure 4.**
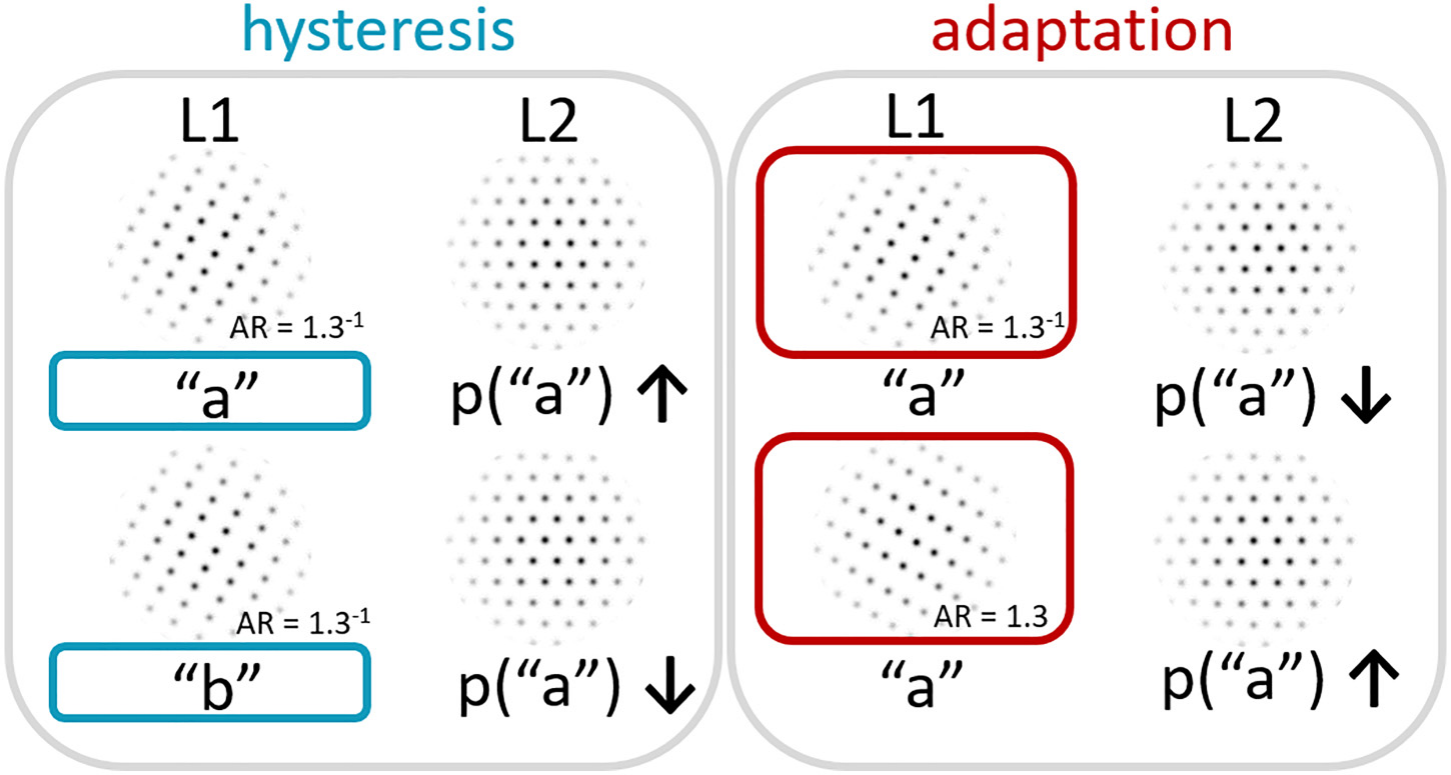
Illustration of attractive and repulsive context effects in dot lattices. Left side: attraction effect (hysteresis). When the first lattice (L1) is perceived as orientation *a*, the probability that the second lattice (L2) will be perceived as orientation *a* is higher than when L1 was interpreted as orientation *b*. Right side: repulsion effect (adaptation). When strong support for orientation *a* is present in L1, the probability that L2 will be perceived as a orientation *a* is lower than when L1 had less support for orientation *a*.

**Figure 5.**
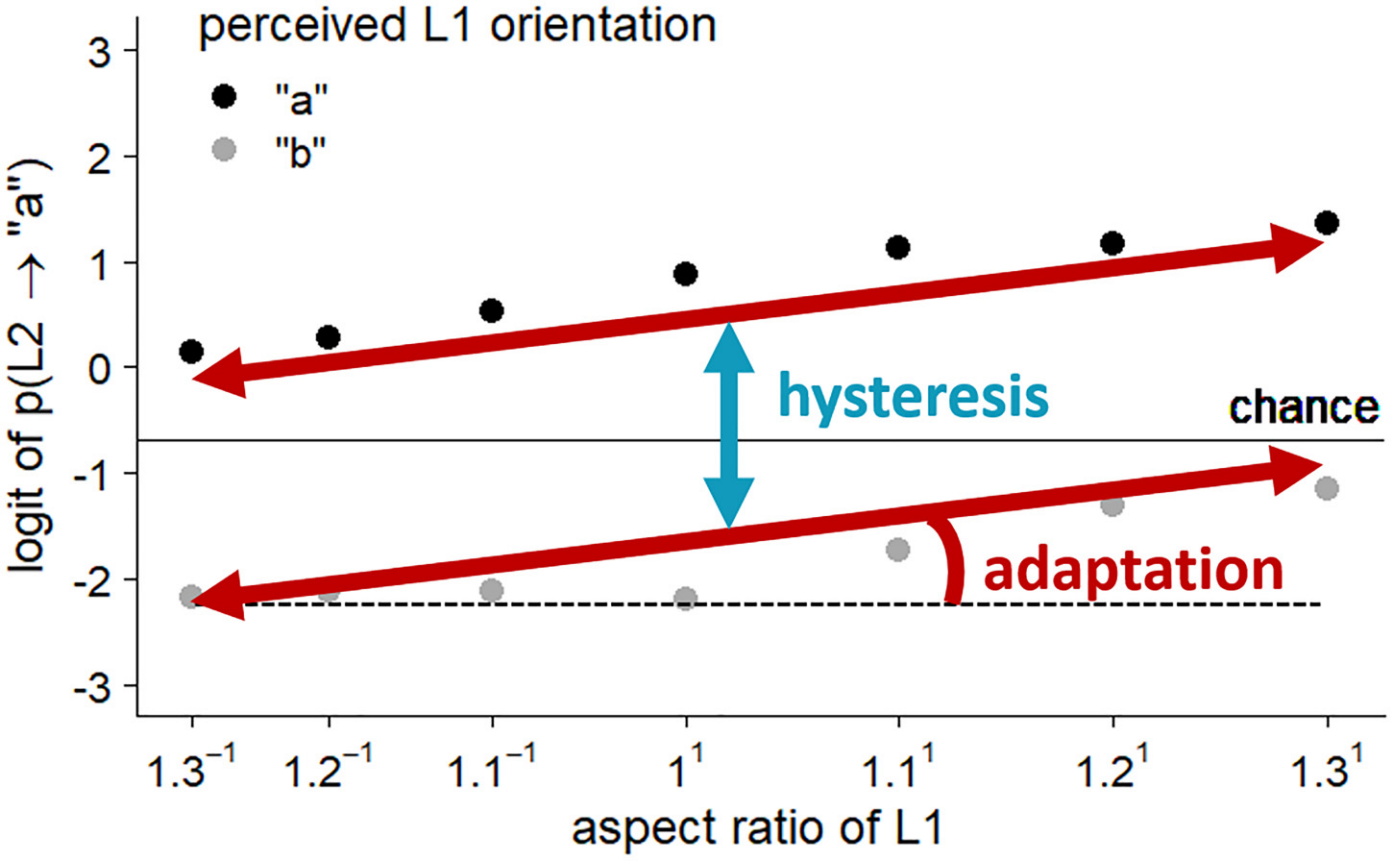
Expected average effects of hysteresis and adaptation on the perception of multistable dot lattices, based on the study of Schwiedrzik et al. (2014). Vertical separation of the two lines reflects the size of the perceptual hysteresis effect, the slope of both lines reflects the size of the perceptual adaptation effect.

Probabilities for perceiving a particular organization in the test stimulus increased when the same organization was perceived in the context stimulus compared to when an alternative organization was perceived in the context stimulus (i.e., hysteresis effect, see the left side of Figure 4). At the same time, the stronger the stimulus support was for a certain organization in the context stimulus (i.e., the closer the dots were together in one dominant orientation compared to the other dominant orientation), the lower the probability was that the same organization was perceived in the test stimulus (i.e., adaptation effect, see the right side of Figure 4). The effects of hysteresis and adaptation were found to combine multiplicatively (in a logistic regression model they related to the current percept independently). Schwiedrzik et al. (2014) used a very similar paradigm as Gepshtein & Kubovy (2005), tested more participants, and added brain imaging to investigate the neural underpinnings of both effects. They found similar behavioral results to those reported by Gepshtein & Kubovy (2005; see Figure 5), and the fMRI data provided evidence for two separate mechanisms underlying adaptation and hysteresis effects, as the effects mapped into distinct cortical networks. In addition, Schwiedrzik et al. (2014) reported interindividual variability in the size of the hysteresis effect, and these individual differences were correlated with differences in activation between hysteresis and no hysteresis trials for the right dorsomedial prefrontal cortex.

Does every individual show these attractive and repulsive context effects, and if so, to the same extent? Although the studies of Gepshtein and Kubovy (2005) and Schwiedrzik et al. (2014) demonstrated the existence of these effects when based on *averaged* data, none of these studies focused on *individual differences* in (the strength of) these temporal context effects.

Earlier work has shown that looking at averaged data alone can be misleading (Kanai & Rees, 2011) and that investigating individual differences can contribute to a richer understanding of visual perception (Mollon et al., 2017). More specifically, when testing the presence of an effect by looking at averaged data alone, one ignores the possibility for large consistent variation between individuals (Kanai and Rees, 2011). Interindividual differences are treated as noise, and it is assumed that the effect would be present for all individuals in case no measurement error would occur. Finding evidence for an average effect however does not guarantee the *true* effect for each individual to be of the same size or in the same direction. The average effect could even be purely an artifact from the averaging procedure (Van der Hulst et al., 2022). Haaf and Rouder (2019) proposed a model comparison approach to tackle exactly these questions: (a) whether the data provide evidence for true, consistent individual differences in the size of an effect, and (b) whether the estimated true effects are in the same direction for all tested individuals. To answer the first question, they compare evidence for a model assuming that individuals share a common effect with no individual variability (i.e., common-effect model) with a model that does not place any constraints on the individuals’ true effects (i.e., unconstrained model). To answer the second question, the unconstrained model is compared with a model that constrains true individuals’ effects to have a particular sign (e.g., to be positive; positive-effects model).

The current study investigates whether there is evidence for true individual differences in the size of hysteresis and adaptation effects, and whether every tested individual shows true hysteresis and adaptation effects in the expected direction. We do this by implementing the model comparison strategy proposed by Haaf and Rouder (2019). Important in this regard is that these model comparisons bring evidence for whether *true* individual variation exists, rather than whether individual variation is *observed* when conducting a task with a finite number of trials (e.g., Mollon et al., 2017). Observed variation between individuals can be due to multiple factors, including trial-by-trial noise, which would not indicate consistent, true interindividual variation. The hierarchical models used in this study allow for the modeling of trial-by-trial variation as well as variation across individuals, and estimate the true individual effects accurately even with a finite number of trials (in contrast, non-hierarchical sample effects are only estimating the true effects accurately in the large-trial limit; Rouder & Haaf, 2019). Establishing whether true individual differences in the size and/or direction of hysteresis and adaptation effects exist is a necessary first step before investigations in the sources and correlates of these true individual differences can become relevant.

The existence of true individual differences can be of theoretical importance (e.g., Haaf & Rouder, 2019; Miller & Schwarz, 2018), and this is the case for individual differences in hysteresis and adaptation effects as well. For example, in case everyone shows both hysteresis and adaptation, this would suggest both to be fundamental mechanisms in human visual perception. In case individuals differ in the extent to which they show hysteresis and adaptation, and the size of both effects is correlated across individuals, this would suggest at least some common factor affecting the processes underlying both effects. In case of evidence for the absence of a correlation between individual hysteresis and adaptation effects, this would imply clearly independent processes underlying the hysteresis and adaptation effects present in this task.

The results may also be important for our understanding of individual differences in perception in general. Interindividual differences in hysteresis and adaptation strength, if they exist, may cause differences in what individuals will actually perceive, even when the current visual input as well as the context stimuli are equal. In case evidence for a lack of interindividual differences is found, this is evidence against differential use of previous percept and previous stimulus context in the formation of the current percept, and differences in hysteresis and adaptation effects can then not explain perceptual differences between individuals given the same stimulus and context. In other words, the study will provide insight in whether individuals can differ in their perception alone (based on differences in previously encountered stimuli and percepts), or whether they can also differ in the processes underlying their perception: whether context info is differentially used across individuals, or whether everyone combines context and current stimulus in a similar way. Put differently, individual differences could either arise through different context information or previous experiences (i.e., previously encountered stimuli and percepts), or alternatively also through how the same context information is incorporated differently by different individuals. The first would imply that consistent individual differences in perception can be due to differences in external factors alone, and that in case everyone would have the same stimulus and perceptual history, everyone would have the same effects of the previous stimulus and previous percept on their current percept. The second would imply that even in case individuals have exactly the same stimulus and perceptual history, there would still be differences in what they perceive due to differential use of the stimulus and perceptual history when coming to the current percept.

Although earlier research has found evidence for individual differences in several tasks assessing hysteresis, adaptation, or their ratio (e.g., Abrahamyan et al., 2016; Mattar et al., 2018; McGovern et al., 2017; Song et al., 2013a, 2013b), only a few studies have attempted to quantify both effects concurrently at the level of the individual participant, by distinguishing the effects of previous stimulus support and previous percept or response (e.g., Bosch et al., 2020; Urai et al., 2017; Zhang & Alais, 2019). Moreover, in none of these studies individual differences in effects of previous stimulus and perceptual choice were the focus of study.

Urai et al. (2017) asked participants to report whether a test stimulus contained stronger or weaker motion than a reference stimulus, and found robust and idiosyncratic patterns of history biases based on previous stimulus and previous choice, with the weight of the preceding choice generally being stronger than the effect of the preceding stimulus. They also found large interindividual variability in the effect of the previous choice, with a majority of the participants showing hysteresis and some showing alternation.

Zhang and Alais (2019) asked participants to report which orientation ( $+45^{\circ }$ or $-45^{\circ }$ ) they perceived in a grating embedded in noise. In a version of the task where motor response and perceptual choice could not be distinguished, they found large individual differences in the effect of the previous choice or response, but rather consistently no effect of the previous stimulus shown. Based on the results from a task in which motor response and perceptual choice could be distinguished, they suggested that individual differences in the sign of the serial dependence reflect different relative weightings of the hysteresis effect for perceptual choice and the adaptation effect for motor response.

Bosch et al. (2020) examined the effects of choice history and evidence history on subsequent perceptual choices by asking participants to identify a coherent motion test stimulus as more or less coherent than a reference stimulus. They found evidence for a bias toward the previous choice, but, at the same time, they found evidence for a bias away from the direction of evidence on the previous trial, especially when it concerned strong evidence. Although almost all participants showed an attractive choice history bias and all participants showed a repulsive evidence history bias, the size of the choice history bias varied considerably across participants (cf. Supplemental Figure 2 in Bosch et al., 2020).

## Hysteresis and adaptation deriving from the same or separate mechanisms?

Whether hysteresis and adaptation effects are the result of the same process or of two separate processes is still under debate. Whereas some argue that both effects can be explained through a single mechanism of sensory integration operating over varying timescales (Mattar et al., 2016), of persistent bias (Gepshtein & Kubovy, 2005), or of neuronal adaptation (Maus et al., 2013), others state that both are separate processes, either in the same neuronal location (e.g., Brascamp et al., 2008) or in distinct cortical networks (Fritsche et al., 2020; Pascucci et al., 2019; Schwiedrzik et al., 2014). Additional arguments for assuming separate mechanisms are differences in the extent to which hysteresis and adaptation are dependent on attention, are modulated by subjective confidence, are modulated by working memory delay, or exhibit clear spatial specificity (for an overview, see Fritsche et al., 2020). Many have also distinguished the effects based on their source being stimulus-related, percept-related, choice-related or motor-related (e.g., Bosch et al., 2020; Carter et al., 2014; Cicchini et al., 2017; Pascucci et al., 2019; Sadil et al., 2021; Zhang & Alais, 2019).

In case individual differences are present in both hysteresis and adaptation, we can also determine the correlation in the size of both effects. A strong correlation between hysteresis and adaptation may suggest at least some common factor affecting the mechanisms underlying both effects, whereas evidence for a correlation close to zero may imply independent processes underlying both effects. Based on a reanalysis of the data from Schwiedrzik et al. (2014),^2^ we expect a positive correlation between individual hysteresis and adaptation effects.

## Hysteresis as a perceptual or decisional effect

Whereas adaptation is typically seen as a stimulus-related effect (e.g., Fritsche et al., 2017; Pascucci et al., 2019; Sadil et al., 2021), there is more debate on the nature of the hysteresis effect. Whereas some “serial dependence” research has suggested the attractive history effect to be the result of a perceptual process (e.g., Carter et al., 2014; Cicchini et al., 2017; Manassi et al., 2018; Schwiedrzik et al., 2018), other research has suggested a post-perceptual, decision-related source of the effect (e.g., Bosch et al., 2020; Fritsche et al., 2017; Pascucci et al., 2019).

In the current study we define hysteresis as a percept-related effect, but it cannot be excluded that the nature of the effect could be related to post-perceptual decision processes rather than perceptual processes. To control for the possibility of the hysteresis effect being a purely decisional rather than a perceptual effect, we included the control task presented by Schwiedrzik et al. (2018) as an additional task in our study. In this control task, the rectangular dot lattices used as context stimuli were replaced by random dot lattices that could not induce the perception of a particular orientation. Participants were then asked to choose between four simultaneously presented orientations. As in the main task, the test stimuli were hexagonal dot lattices, and also here participants chose between four simultaneously presented orientations. In case the hysteresis effect would be a decisional effect rather than a percept-related effect, an influence of the response to the first random dot lattice would still have an effect on the perceived orientation in the test stimulus (i.e., a hysteresis effect would be present). In case the hysteresis effect would be percept-related, no hysteresis effect would be found in this control task.

## Making the distinction between stimulus-related, percept-related, and response-related effects

Whereas the debate has mostly focused on attractive history effects being perceptual or post-perceptual (e.g., Cicchini et al., 2017; Fritsche et al., 2017; Manassi et al., 2018; Pascucci et al., 2019), we argue that it is important to make a distinction between stimulus-related effects on the one hand and percept-, response-, or decision-related effects on the other hand. The mixed results in the serial dependence literature are in our view partially due to the use of paradigms that cannot make this distinction between influences of previous stimuli and previous percepts. In addition, in many studies the distinction between percept, response, or decision is difficult to make. The literature suggesting the hysteresis effect to be post-perceptual has typically argued as follows: When the effect was not stimulus-related, they concluded it to be post-perceptual, and when stimulus-related effects were found those were typically reported as “perceptual”. Making the conceptual distinction between stimulus-related and percept-related effects could help clarify this literature. The earlier findings could potentially be interpreted as evidence for perceptual hysteresis as those studies did not distinguish between percept-related and stimulus-related effects (e.g., Bosch et al., 2020; Fritsche et al., 2017; Pascucci et al., 2019).

## Orientation bias

Earlier research reported effects of absolute orientation of stimuli on performance in several perceptual tasks (i.e., the “oblique effect”; Appelle, 1972) with performance being higher for horizontally or vertically oriented stimuli than for obliquely oriented stimuli. Absolute orientation can not only influence perceptual performance, it may also influence perceptual experience. Kubovy and van den Berg (2002) reported three main bias categories for absolute orientation in the perception of hexagonal dot lattices: preference for vertical, preference for horizontal, and preference for vertical and horizontal over oblique. Some individuals stayed in one bias pattern consistently, others gradually shifted from one pattern to another. In a study by Claessens and Wagemans (2008) observers generally preferred vertical over horizontal orientations, but the exact orientation bias distribution was subject to individual differences. In the present study, the relation of the strength of individual’s absolute orientation bias with the magnitude of their hysteresis and adaptation effects on perception will be investigated. We expect the effects of hysteresis and adaptation to be smaller when a stronger absolute orientation bias is present, as a stronger longer-term absolute orientation bias may overshadow influences of short-term temporal context like hysteresis and adaptation. In other words, we expect that individuals who have a stronger longer-term prior (likely based at least partially on longer-term stimulus history and perceptual history) will be less influenced by shorter-term expectations (i.e., hysteresis) as well as by shorter-term stimulus history (i.e., adaptation).

## Temporal stability of individual differences in hysteresis, adaptation, and orientation bias

Although previous research has investigated the temporal stability of some perceptual biases for motion direction and of grouping behavior in multistable dot lattices (e.g., Van der Hulst et al., 2022; Wexler et al., 2015), we do not know of any research on the temporal stability of individual differences in the *magnitude* of short-term history effects or (assumedly) longer-term perceptual absolute orientation biases. Wexler et al. (2015, Experiment 1) found that, although significant changes in structure-from-motion (SFM) and transparency-from-motion (TFM) bias directions occur, most biases are stable even over periods as long as one year. In addition, they found moderate but robust correlations between daily steps in the SFM and TFM biases, both within and between participants (Experiment 3). Van der Hulst et al. (2022) investigated the consistency of perceptual grouping behavior across two testing sessions that were one day apart. For most participants, behavior in both sessions was moderately to very strongly correlated, indicating that perceptual grouping behavior remained stable across testing sessions.

In this study, we investigate the temporal stability of individual differences in the magnitude of hysteresis and adaptation effects as well as of differences in the magnitude of the absolute orientation bias, by collecting data from the same participants in two sessions at least a week apart (minimally 7 days, maximally 14 days). As there are reasons to believe that the data for the second session may be less informative (e.g., participants may be less motivated for the second session because they already took part in the tasks before, non-random dropout may occur, etc.), all planned analyses (except for the ones on temporal stability) will be conducted based on the data for the first session. When estimating and testing the temporal stability of the hysteresis and adaptation effects, we will use the hierarchical model approach suggested by Rouder & Haaf (2019), as this approach provides a more accurate estimate of the correlation of individuals’ effects between sessions, as it is less affected by design choices (e.g., the number of trials per individual per session) than correlating effects estimated separately for each session (Rouder & Haaf, 2019).

## Research questions and hypotheses

This study thus investigates (a) whether the average attractive and repulsive context effects found in the perception of multistable dot lattices replicate (Gepshtein & Kubovy, 2005; Schwiedrzik et al., 2014), (b) whether consistent individual differences exist in the size of these effects, and (c) whether each individual shows both effects in the expected direction. Furthermore, it investigates (d) whether individual differences in hysteresis or adaptation effects in the dot lattice paradigm discussed are correlated, (e) whether the hysteresis effect is a perceptual or a purely decisional phenomenon, and (f) whether individual differences in hysteresis or adaptation effects in the dot lattice paradigm relate to differences in the strength of individuals’ absolute orientation biases. Finally, we also investigate (g) whether individual differences in the size of hysteresis and adaptation effects as well as in the magnitude of absolute orientation biases are stable across time.

All research questions and hypotheses can be found in detail in Table 1. Firstly, the study serves as a replication of the distinct *average* effects of hysteresis and adaptation on the perception of multistable dot lattices found in Gepshtein & Kubovy (2005) as well as Schwiedrzik et al. (2014; see Figure 5). Regarding the hysteresis effect, we predict that (a) the probability of perceiving orientation $0^{\circ }$ (the a orientation) in the second lattice will be higher when the first lattice is perceived as orientation $0^{\circ }$ than when the first lattice is perceived as orientation $90^{\circ }$ (H1). Regarding the adaptation effect, we predict that the probability of perceiving orientation $0^{\circ }$ in the second lattice will be lower for smaller aspect ratios in the first stimulus (|*a*|/|*b*|): The more the aspect ratio of the first stimulus is in favor of orientation $0^{\circ }$ , the less the second stimulus will be perceived as orientation $0^{\circ }$ (H2). Similar to those previous studies we also hypothesize that the hysteresis and adaptation effects will combine multiplicatively (H3).

**Table 1.** Research questions and hypotheses.

H	Hypothesis	Statistical Test
H1	Perceiving a certain organization in the context stimulus will increase the probability of perceiving that same organization in the test stimulus (i.e., hysteresis effect).	Calculate the Bayes factor in favor of the model including the percept of the first lattice as predictor compared to a model without the percept of the first lattice as predictor, using bridge sampling (Gronau et al., 2017).
H2	The stronger the stimulus support for a certain organization in the context stimulus (i.e., based on aspect ratio), the lower the probability to perceive that organization in the test stimulus (i.e., adaptation effect).	Calculate the Bayes factor in favor of the model including the aspect ratio of the first lattice as predictor compared to a model without the aspect ratio of the first lattice as predictor, using bridge sampling (Gronau et al., 2017).
H3	The hysteresis and adaptation effects described in H1 and H2 will combine multiplicatively and will thus be independent in logit space (i.e., there will be no significant interaction).	Calculate the Bayes factor in favor of the model without interaction between the percept and the aspect ratio of the first lattice as predictor compared to a model with the interaction between the percept and the aspect ratio of the first lattice as predictor, using bridge sampling (Gronau et al., 2017).
H4	Consistent individual differences will exist in the size of the estimated true individual hysteresis and adaptation effects (i.e., a model predicting individual differences in each of these effects will do better than a model predicting the same effect sizes for every participant).	Calculate the Bayes factor in favor of a model including random intercepts and slopes for every participant compared to a model including no random slopes (cf. unconstrained model vs. common effects model in Haaf & Rouder, 2019), using bridge sampling (Gronau et al., 2017). We conduct this model comparison for each effect separately.
H5	Every participant will show the hysteresis and adaptation effects described in H1 and H2 to some extent: Every participant in the study will show an estimated true positive hysteresis effect and an estimated true positive adaptation effect. A model predicting a positive effect size for every participant in the case of both hysteresis and adaptation will do better than a model without constraints on the direction of the effects for every participant.	Calculate the Bayes factor in favor of a model predicting a positive effect size for every participant compared to a model that does not place any order or equality constraints on individuals’ effects, using the encompassing approach (cf. positive effects model vs. unconstrained model in Haaf & Rouder, 2019). In the positive effects model, the main hysteresis and the main adaptation effect are both restricted to be positive. We conduct this model comparison for each effect separately, however.
H6	The size of individuals’ estimated true hysteresis effect will correlate positively with the size of their estimated true adaptation effect.	Calculate the Bayes factor in favor of a model that assumes the true linear correlation to be positive compared to a model assuming a non-positive true linear correlation, using the Savage-Dickey density ratio method (Wagenmakers et al., 2010).
H7	In the control task with a random dot lattice as context stimulus, responding to have perceived a certain organization in the context stimulus will not increase the probability of perceiving that same organization in the test stimulus (i.e., no hysteresis effect).	For the data of the control task, calculate the Bayes factor in favor of the model including the response to the first lattice as predictor compared to a model without the response to the first lattice as predictor, using bridge sampling (Gronau et al., 2017).
H8	The size of individuals’ orientation bias will correlate negatively with the size of their estimated true hysteresis and adaptation effects.	Calculate the Bayes factor in favor of a model that assumes the true linear correlation to be negative compared to a model assuming a non-negative true linear correlation, using the Savage-Dickey density ratio method (Wagenmakers et al., 2010).
H9	The size of individuals’ estimated true hysteresis and adaptation effects at a first timepoint will correlate positively with the size of their estimated true hysteresis and adaptation effects at a second timepoint at least one week later.	Calculate the Bayes factor in favor of a general model that allows for a correlation between individuals’ hysteresis (adaptation) effects across sessions compared to a model that assumes uncorrelated individual hysteresis (adaptation) effects per session, using bridge sampling (Gronau et al., 2017). In addition, compare this general model that allows for a correlation between individuals’ hysteresis (adaptation) effects across sessions with a model that assumes fully correlated individual hysteresis (adaptation) effects across sessions (cf. Rouder & Haaf, 2019).
H10	The size of individuals’ absolute orientation bias as measured at a first timepoint will correlate positively with the size of their absolute orientation bias as measured at a second timepoint at least one week later.	Calculate the Bayes factor in favor of a model that assumes the true linear correlation to be positive compared to a model assuming a non-positive true linear correlation, using the Savage-Dickey density ratio method (Wagenmakers et al., 2010).

Secondly, *individual* hysteresis and adaptation effects are investigated. Based on the methods developed by Haaf and Rouder (2019), we investigate whether consistent individual differences exist in the size of these hysteresis and adaptation effects (H4), by comparing evidence for a model with a common hysteresis effect (a common adaptation effect) across individuals with a model including a variable hysteresis effect (adaptation effect) for every individual (Haaf & Rouder, 2019). In addition, we investigate whether the evidence is in favor of true hysteresis and adaptation effects in the expected direction for everyone (H5), by comparing evidence for a positive effects model and an unconstrained model (Haaf & Rouder, 2019).

Thirdly, we investigate whether individual differences in hysteresis or adaptation effects in the dot lattice paradigm discussed correlate with each other (H6). Furthermore, we examine whether the hysteresis effect is a perceptual or a purely decisional effect by adding a control task in which the first lattice can not induce the perception of a particular orientation (H7). In this control task, we predict the absence of an attractive effect of the response to the first lattice on the percept of the second lattice (i.e., no hysteresis effect). As longer-term biases may diminish the influence of short-term temporal context effects, we also explore whether individual differences in hysteresis or adaptation effects correlate negatively with the strength of the individual’s absolute orientation bias (H8).

Finally, we study the temporal stability of individual differences in the size of individual’s hysteresis and adaptation effects (H9), as well as in the magnitude of individuals’ absolute orientation biases (H10). We predict individual differences in the magnitude of these effects to be correlated positively across sessions.

## Methods

The data collection for this study is part of the data collection for a larger research project. Here we specify all collected measures that are used in the scope of this specific study.

### Participants

Anyone between 18 and 100 years old, with (corrected to) normal vision, and able to understand Dutch instructions was able to participate. Participants were recruited via the faculty’s participant pool, personal contacts of the researchers, social media, and offline advertisements in public places and university buildings. Depending on the wish of the participant, either a monetary compensation of 8 euros per hour or one research credit per hour was offered for participation. The only criteria for exclusion from the analyses concerning the first session were (a) incomplete participation to the first session and (b) choosing the diagonal options in the first lattice in the main task in more than 40% of the trials (this is interpreted as an indication of random responding, based on the law of proximity, Kubovy et al., 1998). For the analyses including the data for both the first and the second session, the exclusion criteria were (a) incomplete participation to the first or the second session and (b) choosing the diagonal options in the first lattice in the main task in more than 40% of the trials in either the first or the second session.

We opted for a sequential Bayes factor design with minimal and maximal n (Schönbrodt & Wagenmakers, 2018). The minimum sample size for the first session was 30 participants. After each five additional participants meeting the inclusion criteria, the Bayes factors related to RQ4 and RQ5 were calculated. Data collection would be terminated when either a Bayes factor of 1/6 or 6 was reached for both main research questions (i.e., H4 and H5 in Table 1)^3^, or when a sample size of 75 participants for the first session was reached (i.e., 2.5 times the original sample size). As we only conduct Bayesian analyses, a sequential stopping rule was allowed and appropriate (Rouder, 2014, 2019; Schönbrodt & Wagenmakers, 2018; Schönbrodt et al., 2017).

Although a Bayes factor of 1/6 or 6 was reached for both main research questions after 55 participants (and we should have stopped according to the preregistered criteria), we continued data collection until data was collected from 75 participants fulfilling the inclusion criteria. The decision to continue was made partly because of logistic reasons (i.e., participation was already scheduled) and partly because of our preference to continue collecting after a sudden direction change in one of the Bayes factors related to H5 going from 50 to 55 participants.^4^ The final sample size for the first session therefore consisted of 75 participants between the ages of 18 and 65 years (59 women, 15 men, one other, $M_{age}=22.56$ years, $SD_{age}=7.92$ years). The data of six participants were excluded from analyses based on the stated exclusion criteria: one participant did not complete the first session and five participants chose the diagonal options in the first lattice in the main task in more than 40% of the trials. The final sample size for the analyses based on the first and the second session consisted of 72 participants between the ages of 18 and 65 years (57 women, 14 men, one other, $M_{age}=22.69$ years, $SD_{age}=8.04$ years). The data of nine participants were excluded from analyses based on the stated exclusion criteria: besides the six participants who were excluded because of reasons related to the first session, one participant did not complete the second session and two participants chose the diagonal options in the first lattice in the main task of the second session in more than 40% of the trials. As the exclusion criteria for analyses related to the first and second session combined focused on the main task, we did include the data from all 75 participants for the visualizations and analyses relating to the absolute orientation bias task only.

### Material

#### Dot lattice stimuli and main task

**Figure 6.**
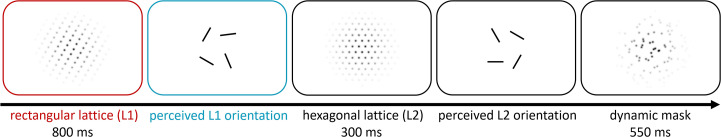
Illustration of trial structure. *Note.* For reasons of visibility, the shown trial components in this figure have black dots on a white background. The actual experiment had white dots on a grey background, as indicated in the task description.

A first version of the dot lattice paradigm that as used here was introduced by Gepshtein and Kubovy (2005) and a modified version was used by Schwiedrzik et al. (2014). Each trial (see Figure 6) consisted of:

- The presentation of a red fixation dot only (1000 ms).
- The presentation of a rectangular dot lattice L1 at a randomly chosen $0^{\circ }$ -orientation (800 ms), on a gray background. The orientation of the lattice was randomized to minimize the accumulation of perceptual bias across trials. The dot lattice had a diameter of 11.5 degrees of visual angle (dva) and the exact position of the dots in the lattice was jittered between 0 and 1.15 dva to prevent that dots of subsequent displays occupy systematically related portions of space. The “dots” were white Gaussian blobs with a diameter of 0.25 dva. The inter-dot distance, here defined as center-to-center distance, was kept to ±1 dva and was varied with aspect ratio so that the product of the distance in the $0^{\circ }$ -orientation and in the $90^{\circ }$ -orientation (|*a*| × |*b*|) was invariant.
- A response screen for reporting the percept of L1 (4-AFC; four icons with lines parallel to possible organizations: $0^{\circ }$ , 90 ${}^{\circ }$ , and two diagonal orientations; duration under observer’s control). The position of the response options was randomized across trials. Once the participant had selected one of the four responses by pressing the corresponding key (*e*/*f*/*i*/*j*), a green circle appeared around the chosen orientation (for 200 ms) and the experiment automatically progressed. This was followed by an additional 100 ms interval, which made the interval between response to the first lattice and the presentation of the second lattice 300 ms.
- The presentation of a hexagonal dot lattice L2 at the same randomly chosen 0 ${}^{\circ }$ -orientation as dot lattice L1 (300 ms), on a gray background. The same diameters and inter-dot distances were applied as in (b).
- A response screen for reporting the percept of L2 (4-AFC; four icons with line parallel to possible organizations: 0 ${}^{\circ }$ , 60 ${}^{\circ }$ , 120 ${}^{\circ }$ , and 90 ${}^{\circ }$ ; duration under observer’s control). The position of the response options was randomized across trials. Once the participant had selected one of the four responses by pressing the corresponding key (*e*/*f*/*i*/*j*), a green circle appeared around the chosen orientation (for 200 ms) and the experiment automatically progressed. This was followed by an additional 100 ms interval, which made the interval between response to the second lattice and the presentation of the mask 300 ms.
- Mask presented on a gray background (550 ms; dynamic random dot mask updated at 25 Hz).

The red fixation dot was continuously present in the center of the screen. Participants were instructed to fixate on the central fixation dot, and to report the first perceived organization in case the percept switched during the presentation period of the target stimulus (either L1 or L2). There were 21 practice trials to get participants acquainted with the task.

The independent variable is the inter-dot distance ratio in the first dot lattice stimulus (i.e., |*a*|/|*b*| = aspect ratio of L1). This ratio varied between $1.3^{-1}$ and 1.3, with values of $1.3^{-1}$ , $1.2^{-1}$ , $1.1^{-1}$ , 1, 1.1, 1.2, and 1.3.

The dependent variables are the individual reports of the percept of the first (L1) and of the second dot lattice (L2) in each trial. Dominant percepts at aspect ratio equal to 1 are parallel to the orientations 0 ${}^{\circ }$ and 90 ${}^{\circ }$ in the first lattice, and parallel to orientations 0 ${}^{\circ }$ , 60 ${}^{\circ }$ , and 120 ${}^{\circ }$ in the second lattice.

The 0 ${}^{\circ }$ -orientation in each trial was randomly chosen, covering 90 ${}^{\circ }$ in steps of 1 ${}^{\circ }$ .

As in Schwiedrzik et al. (2014), each participant was asked to complete nine blocks of 70 trials, with 10 trials for each of the seven aspect ratios per block. The order of trials was pseudorandomized: each aspect ratio occurred equally often in each block, but otherwise the order within each block was randomized. Furthermore, the location of the four response options within and between trials was also randomized.

#### Control task

To control for the possibility of the hysteresis effect being a purely decisional rather than a perceptual effect, we included the control task presented by Schwiedrzik et al. (2018) as an additional task in our study. This control task was equal to the main task, with the exception of the presentation of the first lattice. In this control task, the first lattice in each trial was a random dot lattice instead of a rectangular dot lattice, as this random dot lattice cannot induce a particular orientation. The response screen for the first lattice in each trial included the relative 0 ${}^{\circ }$ , 90 ${}^{\circ }$ , 45 ${}^{\circ }$ , and 135 ${}^{\circ }$ orientations (i.e., the two diagonal orientations for a lattice with an aspect ratio of 1). Each participant was asked to complete one block of 90 trials. The order of the trials was randomized, as well as the location of the four response option within and between trials. There were three practice trials to get participants acquainted with the task.

#### Absolute orientation bias

As we expected the effects of hysteresis and adaptation to be smaller when a strong absolute orientation bias was present, we included a task with ambiguous hexagonal dot lattices only, varying in absolute orientation with the a orientation from 1 ${}^{\circ }$ to 60 ${}^{\circ }$ . In every hexagonal lattice, six different orientations can be perceived, of which three are most and equally dominant in general. Four blocks of 60 trials were presented, with every absolute orientation shown once per block and the presentation order randomized within each block. There were five practice trials to get participants acquainted with the task.

Each trial consisted of:

- The presentation of a red fixation dot only (750 ms).
- The presentation of a hexagonal dot lattice at a randomly chosen 0 ${}^{\circ }$ -orientation varying between 1 ${}^{\circ }$ and 60 ${}^{\circ }$ (500 ms), on a gray background. The same diameters and inter-dot distances were applied as in the main task described above.
- A response screen for reporting the percept of the hexagonal lattice (4-AFC; four icons with lines parallel to possible organizations: 0 ${}^{\circ }$ , 60 ${}^{\circ }$ , 120 ${}^{\circ }$ , and 90 ${}^{\circ }$ ; duration under observer’s control). The position of the response options was randomized across trials. Once the participant had selected one of the four responses by pressing the corresponding key (*e*/*f* /*i*/*j*), a green circle appeared around the chosen orientation (for 200 ms) and the experiment automatically progressed. This was followed by an additional 200 ms interval, which made the interval between response to the lattice and presentation of the next 1150 ms (200 ms feedback, 200 ms interval, 750 ms fixation dot).

### Procedure

The experimental sessions took place in a darkened room using a cathode ray tube monitor ViewSonic G90fB, 1024 by 768 pixels, at 60 cm distance, refresh rate 60 Hz. Participants’ stable head position was guaranteed by using a chinrest with forehead support. The dot lattice stimuli were generated in Matlab 2018b using the code of Schwiedrzik et al. (2014).^5^ Stimulus presentation and response collection was controlled using Python 3 (Van Rossum & Drake, 1995) and the PsychoPy library (Peirce, 2007). In the first session, participants first completed the orientation bias task, then the main task measuring hysteresis and adaptation, and finally the control task. In the second session, participants completed the orientation bias task and the main task measuring hysteresis and adaptation for the second time. The second session took place at least one week after the first session, with a minimum of 7 days and a maximum of 14 days apart.^6^

### Data analysis

We used R [Version 4.0.4; R Core Team (2021)] for all our analyses.^7^ All models were fitted using the R package brms (Bürkner, 2017, 2018). The analysis procedure described below (except for the analyses related to H7, H8, and H10) had been worked out and was tested on the data previously collected by Schwiedrzik et al. (2014).^8^

#### Preprocessing

Planned analyses were restricted to the response alternatives with equal likelihood at aspect ratio equal to 1. This means that only trials in which participants responded 0 ${}^{\circ }$ or 90 ${}^{\circ }$ for the first lattice and 0 ${}^{\circ }$ , 60 ${}^{\circ }$ , or 120 ${}^{\circ }$ for the second lattice were used. For this reason, we excluded 9,026 out of 47,250 trials (19.10%) from analyses of the main task in the first session, as well as 3,807 out of 6,750 trials (56.40%) for the control task in the first session^9^ , and 6,434 out of 45,360 trials (14.18%) for the main task in the second session. In the absolute orientation bias task, 1,848 out of 18,000 trials (10.27%) with 90 ${}^{\circ }$ responses were excluded in the first session, and 1,045 out of 18,000 trials (5.81%) in the second session.

For visualization purposes, we computed, per participant and on average, the logit of the probability to perceive the 0 ${}^{\circ }$ orientation in the first stimulus (i.e., logit[*p*(*l*1 $\to 0^{\circ }$ )]) and the logit of the probability to perceive the 0 ${}^{\circ }$ orientation in the second stimulus given that the first stimulus was perceived as orientation 0 ${}^{\circ }$ or orientation 90 ${}^{\circ }$ (i.e., logit[*p*(*l*2 $\to 0^{\circ }$ )] for *l*1 $\to 0^{\circ }$ and for *l*1 $\to 90^{\circ }$ ) to overcome floor effects at high aspect ratios^10^ :

$$logit[p(l1\to 0^{\circ })]=ln\left[\frac{\;p(l1\to 0^{\circ })}{1-p(l1\to 0^{\circ })}\right]$$

and

$$logit[p(l2\to 0^{\circ })]=ln\left[\frac{\;p(l2\to 0^{\circ })}{1-p(l2\to 0^{\circ })}\right].$$

To determine the preferred orientation direction and the size of the individual’s absolute orientation bias, we calculated the direction and magnitude of the orientation vector per participant (cf. Curray, 1956). The orientation vector is the vector of all chosen orientations, excluding trials in which participants chose the unlikely 90 ${}^{\circ }$ orientation in the hexagonal lattices (1,848 trials out of 18,000 were excluded for this reason in the first session and 1,045 trials out of 18,000 in the second session). The vector direction can be interpreted as the preferred orientation direction, whereas the vector magnitude, which varies from 0% to 100%, can be interpreted as the strength of the absolute orientation bias. Vector magnitude ( L ) and direction ( $\bar{\theta }$ ) were calculated as follows (Curray, 1956):

$$\begin{array}{rl} L & =\frac{\sqrt{(\sum n\;sin\;2\;\theta {)}^{2}+(\sum n\;cos\;2\;\theta {)}^{2}}}{\sum n}*100\\ \bar{\theta } & =\frac{1}{2}\;arctan\frac{\sum n\;sin\;2\;\theta }{\sum n\;cos\;2\;\theta }. \end{array}$$

#### Data visualizations

We plot the average and individual results on probability scale and logit scale for perceiving the first lattice as orientation 0 ${}^{\circ }$ (Y-axis: logit[*p*(*l*1 $\to 0^{\circ }$ )]; X-axis: aspect ratio L1) and for perceiving the second lattice as orientation 0 ${}^{\circ }$ (*Y* -axis: logit[*p*(*l*2 $\to 0^{\circ }$ )]; *X*-axis: aspect ratio L1; grouping var = *l*1 $\to 0^{\circ }$ or *l*1 $\to 90^{\circ }$ ). As the relative grouping strength of the dots in a lattice among a certain orientation has been shown to follow a decreasing exponential trend in function of the relative inter-dot distance in that orientation (Kubovy et al., 1998), the logit of the probability is approximately linear. Vertical separation of the two lines reflects the size of the perceptual hysteresis effect; the slope of both lines reflects the size of the perceptual adaptation effect. We also plot the results regarding absolute orientation bias, on average, per individual, and per block.

Regarding the individual estimates of the hysteresis and adaptation effect, we plot mean estimates and 95% highest density continuous intervals for the hysteresis and adaptation effect separately, the correlation between individual hysteresis and adaptation effects, as well as the correlation between the individual orientation bias and the size of the estimated individual hysteresis and adaptation effects.

#### Model estimation

The full model used to estimate individual hysteresis and adaptation effects is a Bayesian multilevel binary logistic regression model predicting the percept of the second lattice ( $Y_{ijkl}$ ), with aspect ratio of the first lattice ( AR ) and the percept of the first lattice ( R10 ) as fixed and random effects. The model thus includes fixed and individual random effects for percept in the first lattice (i.e., hysteresis effect) as well as aspect ratio in the first lattice (i.e., adaptation effect), and individual random intercepts.

$Y_{ijkl}$ stands for the response variable, more specifically the percept of the second lattice, for the *l*th replicate for the *i*th participant, *i* = 1, …, *I* in the *j*th condition for aspect ratio of the first lattice ( AR ), *j* = 1, …, 7 and the *k*th condition for the percept of the first lattice ( R10 ), *k* = 1, 2 with *l* = 1, …, $L_{ijk}$ . I is the number of participants in the data. $Y_{ijkl}$ is modeled to follow a Bernoulli distribution with a probability $p_{ijkl}$ of the second lattice being perceived as the 0 ${}^{\circ }$ orientation. The percept of the first and the second lattice can be 0 (when different from the 0 ${}^{\circ }$ orientation in the lattice) or 1 (when equal to the 0 ${}^{\circ }$ orientation in the lattice). Centered aspect ratio was used, which means that a value of zero corresponds to an aspect ratio of 1, a value of $1.1^{-1}-1$ (i.e., ≈ −0.09 ) corresponds to $1.1^{-1}$ , and a value of $1.1^{1}-1$ (i.e., 0.10) to an aspect ratio of 1.1.

$$\begin{array}{rl} & Y_{ijkl}∼Bernoulli(p_{ijkl})\\ & log\left(\frac{\;p_{ijkl}}{1-p_{ijkl}}\right)=\beta_{0}+\beta_{\;j}AR+\beta_{k}R10+\beta_{i0}+\beta_{ij}AR+\beta_{ik}R10 \end{array}$$

$\beta_{0}$ represents the fixed intercept, whereas $\beta_{\;j}$ and $\beta_{k}$ represent the fixed adaptation and hysteresis effect, respectively. $\beta_{i0}$ , $\beta_{ij}$ , and $\beta_{ik}$ represent the individual random intercepts, the individual random slopes of aspect ratio of the first lattice (i.e., adaptation effects), and the individual random slopes of percept of the first lattice (i.e., hysteresis effect), respectively. Another way to formulate the model is:

R20∼Intercept+AR+R10+(Intercept+AR+R10|participant).

**Figure 7.**
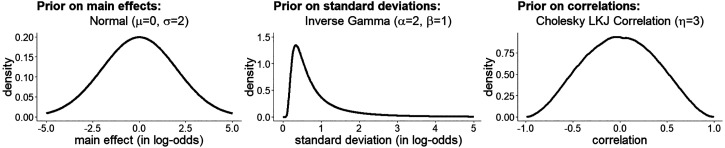
Illustration of priors used in the model predicting the percept of L2.

Figure 7 visualizes the priors we specified for the fixed effects, for the standard deviation of the random effects, and for the correlation matrix.

We fitted this model of perceived L2 orientation using brms (Bürkner, 2017, 2018). We used four chains with 20,000 iterations each with the default number of warmup iterations per chain. In case of computational issues we could have decided to deviate from the specified number of iterations, but this was not necessary. We used a delta equal to .8 and a maximum treedepth of 10. For any other sampling specifications we used the default settings when possible.

#### Average hysteresis effect (H1)

To test the presence of an average hysteresis effect across individuals, we compared a model including the percept of the first lattice as predictor versus a model without the percept of the first lattice as predictor and calculated the Bayes factor in favor of the model including the hysteresis effect, using bridge sampling (Gronau et al., 2017). In case the Bayes factor was in favor of the model including the hysteresis effect, we report the mean and 95% highest density continuous interval (HDCI) for the coefficient related to the percept of L1 in the full model described above, to have an estimate of the size of the average hysteresis effect.

#### Average adaptation effect (H2)

To test the presence of an average adaptation effect across individuals, we compared a model including the aspect ratio of the first lattice as predictor versus a model without the aspect ratio of the first lattice as predictor and calculated the Bayes factor in favor of the model including the adaptation effect, using bridge sampling (Gronau et al., 2017). In case the Bayes factor was in favor of the model including the adaptation effect, we report the mean and 95% highest density continuous interval (HDCI) for the coefficient related to the aspect ratio of L1 in the full model, to have an estimate of the size of the average adaptation effect.

#### Independence of average hysteresis and adaptation effects (H3)

To test the independence of the average hysteresis and adaptation effects, we compared a model including the interaction between the percept and the aspect ratio of the first stimulus as predictor versus a model without the interaction and calculated the Bayes factor in favor of the model without the interaction, using bridge sampling (Gronau et al., 2017). In case the Bayes factor was in favor of the model including the interaction effect, we report the mean and 95% highest density continuous interval (HDCI) for the interaction coefficient in a full model including the interaction and all random effects, to have an estimate of the size of the average interaction effect.

#### Individual hysteresis and adaptation effects: Do individual effects differ? (H4)

To test whether individual hysteresis and adaptation effects differ in size, we calculated the Bayes factor in favor of a model including random intercepts and slopes for every participant compared to a model including no random slopes (cf. unconstrained model vs. common effects model in Haaf & Rouder, 2019), using bridge sampling (Gronau et al., 2017). We conducted this model comparison for each effect separately.

#### Individual hysteresis and adaptation effects: Does everyone show the effects? (H5)

To test whether every individual participant shows a positive hysteresis or adaptation effect, we calculated the Bayes factor in favor of a model predicting a positive effect size for every participant compared to a model that does not place any order or equality constraints on individuals’ effects, using the encompassing approach (cf. positive effects model vs. unconstrained model in Haaf & Rouder, 2019). In the positive-effects model, the main hysteresis and the main adaptation effect are both restricted to be positive. The model comparison was done for each effect separately, however.

#### Does the size of hysteresis and adaptation effects correlate positively across individuals? (H6)

To determine the size of the hysteresis effect, we used the individual estimates for the effect of the percept of the first lattice on the percept of the second lattice. To determine the size of the adaptation effect, we used the individual estimates for the effect of aspect ratio of the first lattice on the percept of the second lattice. These estimates are based on the Bayesian model of the percept of the second lattice described above, with the aspect ratio of the first lattice and the percept of the first lattice as fixed effects, with random intercepts and random slopes for both hysteresis and adaptation effects.

To test whether the size of individuals’ hysteresis effect correlates positively with the size of their adaptation effect, we calculated the Bayes factor in favor of a model that assumes the true linear correlation to be positive compared to a model assuming a non-positive true linear correlation using the Savage-Dickey density ratio method (Wagenmakers et al., 2010). As this is a one-sided hypothesis, the Bayes factor is equal to the posterior probability under the hypothesis (*r* > 0) against its alternative (*r* <= 0). To have an estimate of the strength of the correlation, we report the mean and 95% HDCI for the correlation between estimated individual hysteresis and adaptation effects, based on the full model described above.

#### Is the hysteresis effect absent in the control task? (H7)

To test the presence of an average hysteresis effect across individuals in the control task, we compared a model including the response to the first lattice as predictor versus a model without the response to the first lattice as predictor and calculated the Bayes factor in favor of the model without the hysteresis effect, using bridge sampling (Gronau et al., 2017). In case the Bayes factor was in favor of the model including the hysteresis effect, we report the mean and 95% highest density continuous interval (HDCI) for the coefficient related to the response to the first lattice in a model including the response to the first lattice as a main and random effect, to have an estimate of the size of the effect.

#### Do individual differences in absolute orientation bias correlate negatively with hysteresis and adaptation effects? (H8)

To test whether the size of individuals’ orientation bias correlates negatively with the size of their hysteresis and adaptation effects, we calculated the Bayes factor in favor of a model that assumes the true linear correlation to be negative compared to a model assuming a non-negative true linear correlation, using the Savage-Dickey density ratio method (Wagenmakers et al., 2010). As this is a one-sided hypothesis, the Bayes factor is equal to the posterior probability under the hypothesis (*r* < 0) against its alternative (*r* >= 0). We conducted this model comparison for each effect separately. To have an estimate of the strength of the correlation, we report the mean and 95% HDCI for the correlation between individual orientation bias estimates and individual hysteresis (adaptation) effects.

#### Does the size of individuals’ hysteresis and adaptation effects correlate positively across timepoints? (H9)

To test whether the size of individuals’ hysteresis effect correlates positively across timepoints, we calculated the Bayes factor in favor of a general model that allows for a correlation between individuals’ hysteresis (adaptation) effects across sessions compared to a model that assumes uncorrelated individual hysteresis (adaptation) effects per session (Rouder & Haaf, 2019), using bridge sampling (Gronau et al., 2017). In addition, we compared this general model that allows for a correlation between individuals’ hysteresis (adaptation) effects across sessions with a model that assumes fully correlated individual hysteresis (adaptation) effects across sessions (Rouder & Haaf, 2019). We conducted these model comparisons for each effect separately. To have an estimate of the strength of the temporal stability, we report the mean and 95% HDCI for the correlation between individual hysteresis (adaptation) estimates across sessions, based on the winning model (in case the winning model is not the model assuming the absence of a correlation).

#### Does the size of individuals’ absolute orientation biases correlate positively across timepoints? (H10)

To test whether the size of individuals’ absolute orientation bias correlates positively across timepoints, we calculated the Bayes factor in favor of a model that assumes the true linear correlation to be positive compared to a model assuming a non-positive true linear correlation using the Savage-Dickey density ratio method (Wagenmakers et al., 2010). As this is a one-sided hypothesis, the Bayes factor is equal to the posterior probability under the hypothesis (*r* > 0) against its alternative (*r* <= 0). To have an estimate of the strength of the temporal stability, we report the mean and 95% HDCI for the correlation between individual orientation bias estimates across sessions.

## Results

**Figure 8.**
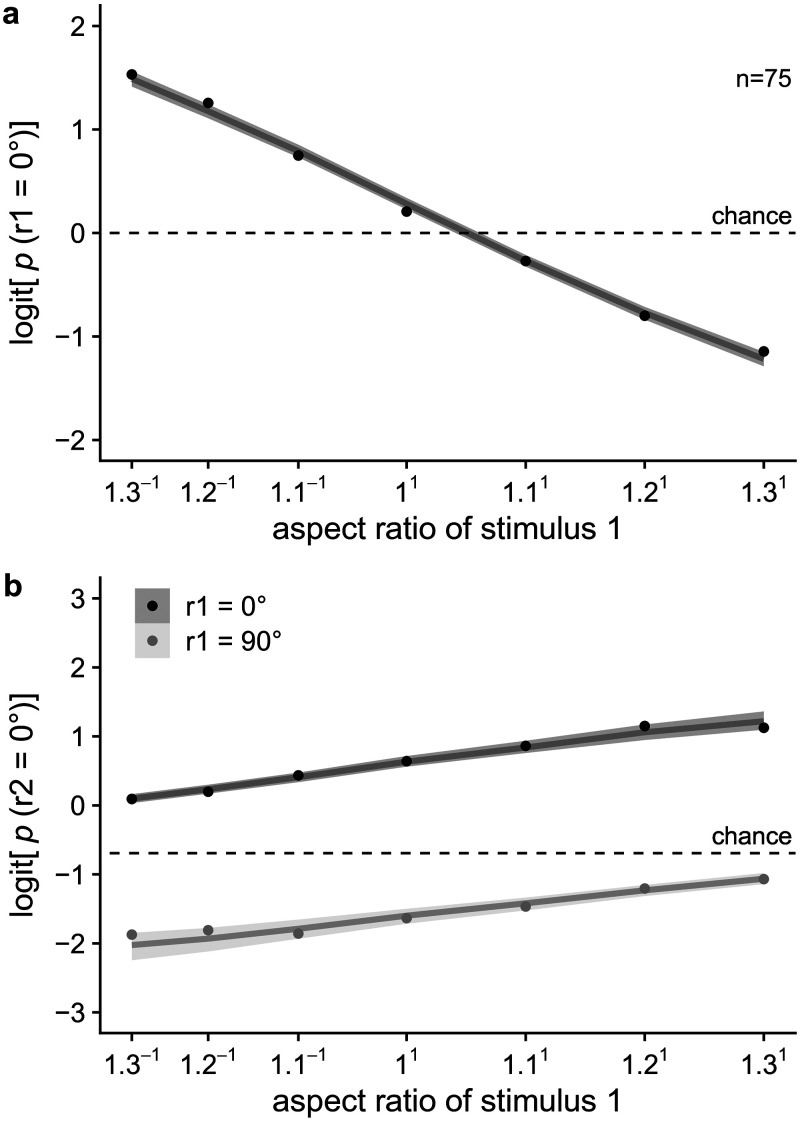
(a) Mean response to the first stimulus dependent on aspect ratio (logit). The probability of responding 0 ${}^{\circ }$ to the first stimulus decreases with aspect ratio (|*a*|/|*b*|). The value of aspect ratio increases with increasing distance in the 0 ${}^{\circ }$ -orientation, leading to more 90 ${}^{\circ }$ responses. (b) Mean response to the second stimulus dependent on aspect ratio (logit). The probability of responding 0 ${}^{\circ }$ to the second stimulus increases with aspect ratio (|*a*|/|*b*|; i.e., adaptation effect), and increases when the first stimulus was perceived as 0 ${}^{\circ }$ rather than 90 ${}^{\circ }$ (i.e., hysteresis effect). Dots indicate observed values. In addition, mean posterior predictions and their 95% highest density continuous intervals are shown.

**Figure 9.**
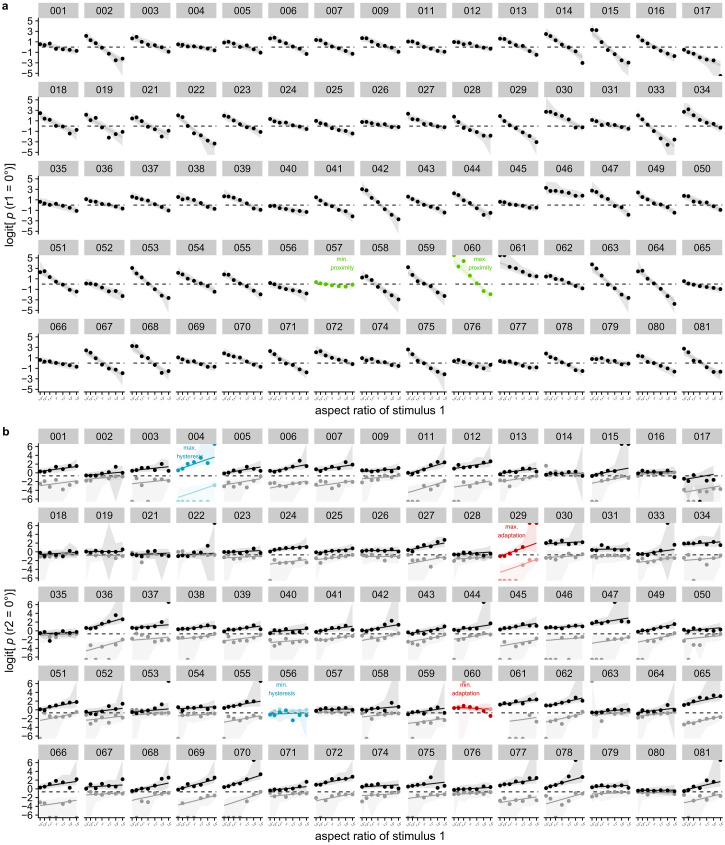
(a) Mean individual responses to the first stimulus dependent on aspect ratio (logit). The probability of responding 0 ${}^{\circ }$ to the first stimulus decreases with aspect ratio (|*a*|/|*b*|). The value of aspect ratio increases with increasing distance in the 0 ${}^{\circ }$ -orientation, leading to more 90 ${}^{\circ }$ responses. Dots indicate observed values. In addition, mean posterior predictions and their 95% highest density continuous intervals are shown. Plots for participants with the smallest and largest estimated proximity effect are indicated in green. (b) Mean individual responses to the second stimulus dependent on aspect ratio (logit). The probability of responding 0 ${}^{\circ }$ to the second stimulus increases with aspect ratio (|*a*|/|*b*|; i.e., adaptation effect), and increases when the first stimulus was perceived as 0 ${}^{\circ }$ rather than 90 ${}^{\circ }$ (i.e., hysteresis effect). Dots indicate observed values. In addition, mean posterior predictions and their 95% highest density continuous intervals are shown. Plots for participants with the smallest and largest estimated hysteresis effect are indicated in blue, participants with the smallest and largest estimated adaptation effect are indicated in red.

In Figures 8 and 9 one can find the results on logit scale on average and per participant respectively. The same figures representing the results on probability scale can be found in the Supplemental Appendix (see Figures A1 and A2). In addition, graphs using the alternative logit calculation as used by Gepshtein and Kubovy (2005) and Schwiedrzik et al. (2014) are provided in the Supplemental Appendix too (see Figures A3 and A4).

### Confirmatory analyses

#### Average hysteresis and adaptation effects? (H1-2)

The Bayes factor in favor of the model including the influence of the L1 percept is very large, with the exact value outside of computer precision. This means that the data are more likely under the model with the hysteresis effect. The Bayes factor in favor of the model including the influence of aspect ratio on the second lattice is $8\times 10^{25}$ . This means that the data are more likely under the model with the adaptation effect. For a visual representation of the average predicted hysteresis and adaptation effects in the full model, see Figures 8 and A1.

**Figure 10.**
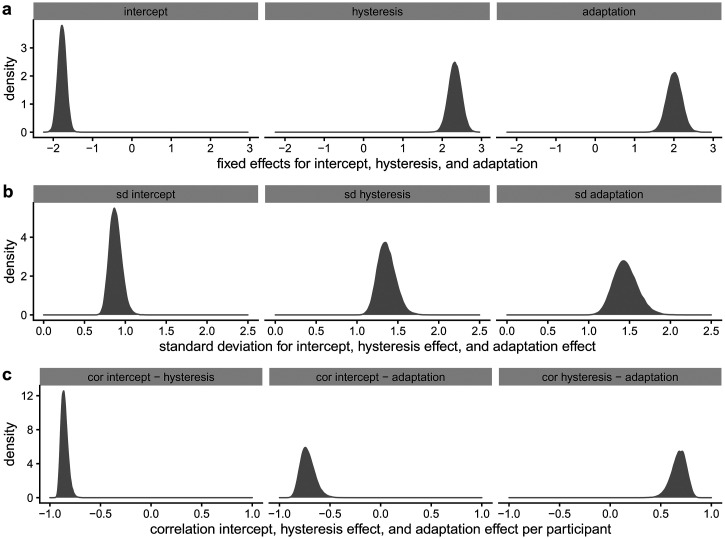
Posterior distributions of fixed effects, standard deviation of random effects, and the correlation between the random effects for the model of perceived L2 orientation.

**Figure 11.**
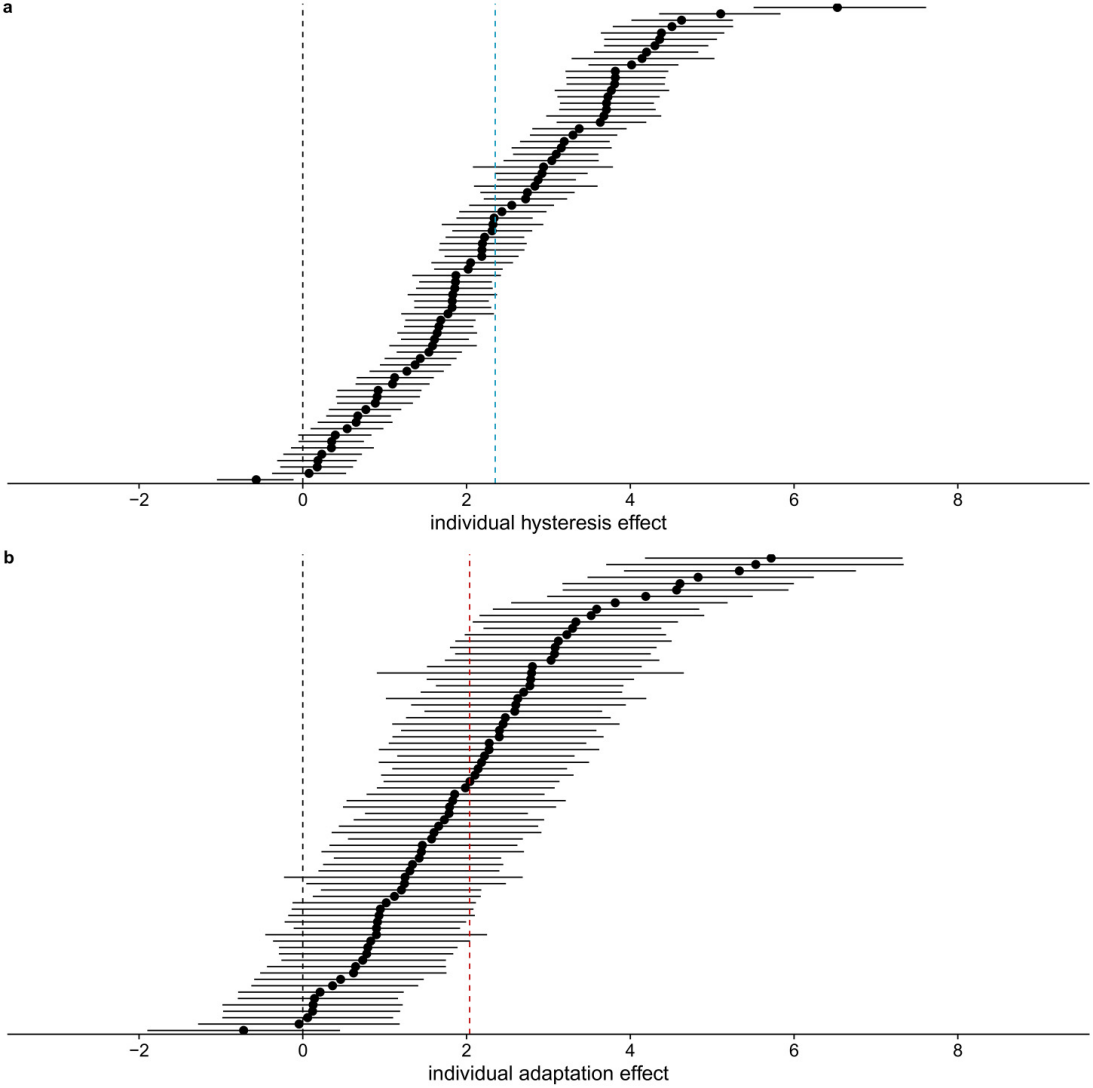
Slopes for the effect of perceived L1 orientation and aspect ratio on perceiving the 0 ${}^{\circ }$ orientation in L2 per participant. Mean and 95% highest density continuous intervals are shown. The colored line indicates the average mean effect across participants. The black line indicates a slope of zero.

Figure 10 shows the posterior distributions of the fixed effects, standard deviation of random effects, and the correlation between the random effects in the model predicting the perceived orientation in the second lattice. Figure 10a shows the posteriors for the effect of the perceived orientation in the first lattice (i.e., hysteresis effect) and the effect of aspect ratio (i.e., adaptation effect) on the perceived orientation in the second lattice. The 95% highest density continuous interval for the main hysteresis effect ranges from 2.01 to 2.64. The 95% highest density continuous interval for the main adaptation effect ranges from 1.64 to 2.38. Figure 11 shows the estimated individual effects of perceived L1 orientation and aspect ratio of L1 in the model predicting perceived L2 orientation.

#### Absence of interaction effect between hysteresis and adaptation? (H3)

The Bayes factor in favor of the model including no interaction compared to the model including an interaction is 7.3039 . This means that the data are more likely under the model without the interaction between the hysteresis and adaptation effect.

#### Are there individual differences in the size of hysteresis and adaptation effects? (H4)

The Bayes factor in favor of the model with a random effect for percept L1 (i.e., a random hysteresis effect) compared to the common effects model is very large, with the exact value outside of computer precision. This means that the observed data are more likely under the unconstrained model than under the common effects model. The Bayes factor in favor of the model with a random effect for aspect ratio (i.e., a random adaptation effect) compared to the common effects model is $2\times 10^{45}$ . This means that the observed data are more likely under the unconstrained model than under the common effects model. These Bayes factors indicate that it is much more likely to assume individual differences in both the hysteresis and adaptation effects than to assume everyone to show the same effect sizes.

#### Does everyone show hysteresis and adaptation? (H5)

The Bayes factor comparing the likelihood of the observed data under the positive effects model and under the unconstrained model for the percept of L1 (i.e., hysteresis effect) is 0.0228 (inverse BF: 43.8232 ). This means that the observed data are less likely under the positive effects model than under the unconstrained model. The Bayes factor comparing the likelihood of the observed data under the positive effects model and under the unconstrained model for aspect ratio of L1 (i.e., adaptation effect) is 0.0145 (inverse BF: 69.1914 ). This means that the observed data are less likely under the positive effects model than under the unconstrained model. These Bayes factors indicate that it is more likely to assume that not everyone shows a hysteresis or adaptation effect than to assume that everyone shows these effects.

#### Correlation between individual hysteresis and adaptation effects? (H6)

**Figure 12.**
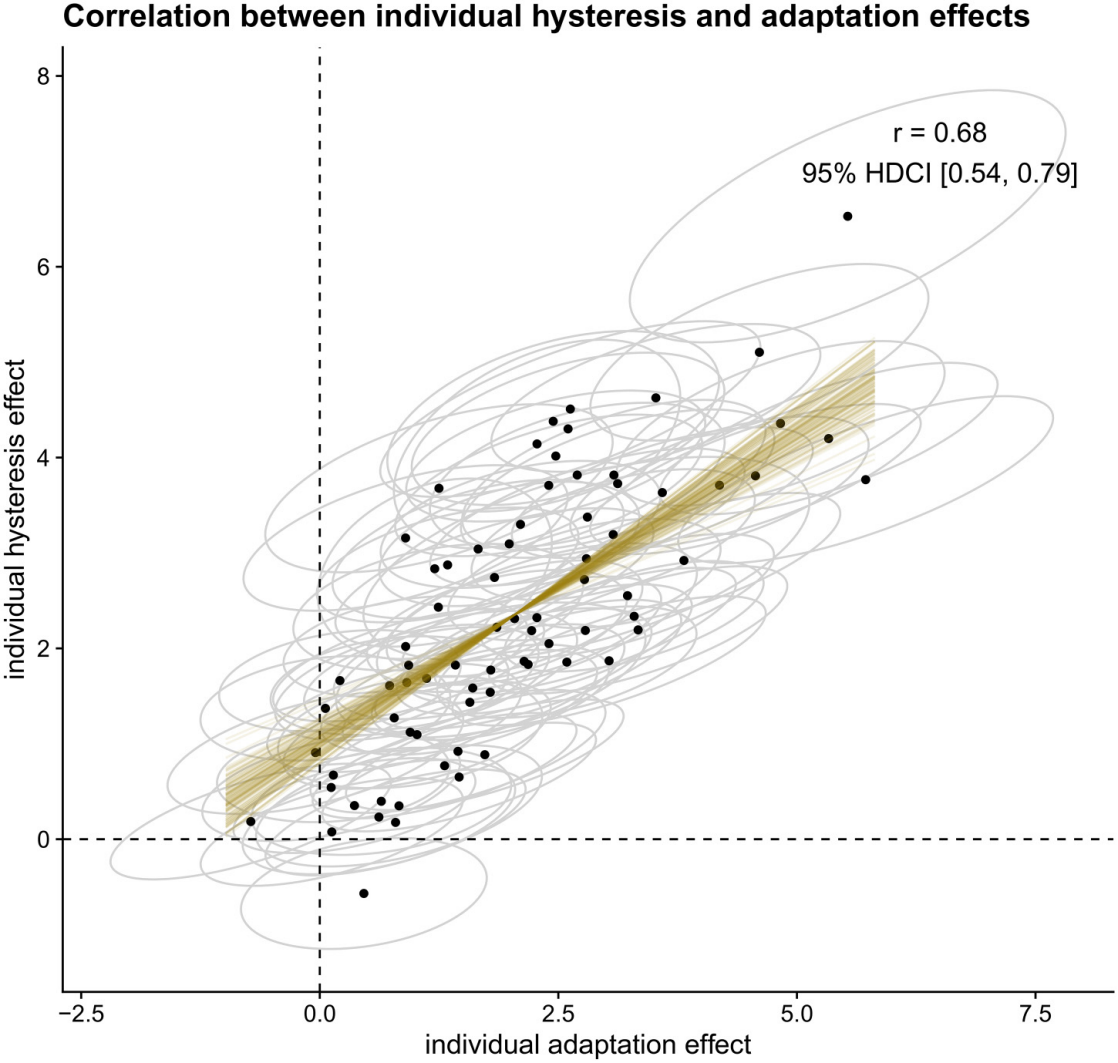
Correlation between individual slopes for the effect of aspect ratio and perceived L1 orientation on perceiving the 0 ${}^{\circ }$ orientation in L2. Mean and 80% highest density continuous intervals per individual are shown. The black lines indicate a slope of zero. The colored lines give examples of plausible correlation estimates. *Note.* As the estimated correlation value shown comes from a hierarchical model including both estimates of the hysteresis and the adaptation effect, potential attenuation of the correlation as a result of noise is already taken into account.

Figure 12 shows the correlation between the individual slopes for aspect ratio and perceived L1 orientation in the model predicting perceived L2 orientation. The Bayes factor in favor of a model that assumes the true linear correlation to be larger than zero compared to a model assuming a true linear correlation smaller than or equal to zero is larger than $1\times 10^{4}$ . This means that the observed data are more likely under the model assuming a positive linear correlation between individual hysteresis and adaptation effects than under the model assuming a non-positive linear correlation. The 95% highest density continuous interval for the correlation between individual effects of perceived L1 orientation and aspect ratio on the perceived L2 orientation ranges from 0.53 to 0.81.

#### Absence of hysteresis effect in the control task? (H7)

**Figure 13.**
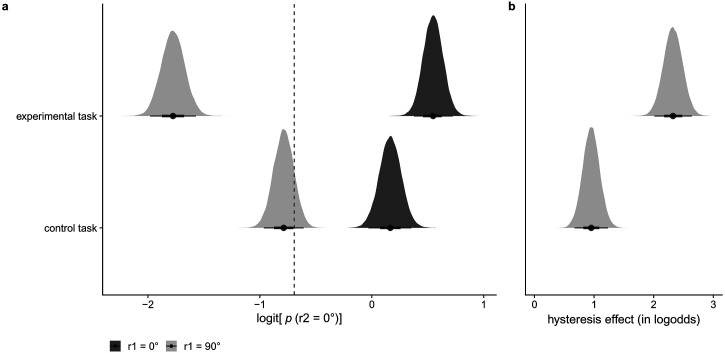
(a) Posterior distribution for the percept of the second lattice separately for each percept of the first lattice in the control task and in the experimental task. (b) Estimated hysteresis effect in the control task and the experimental task. Mean, 66%, and 95% highest density continuous intervals are shown. The vertical black line indicates chance level.

**Figure 14.**
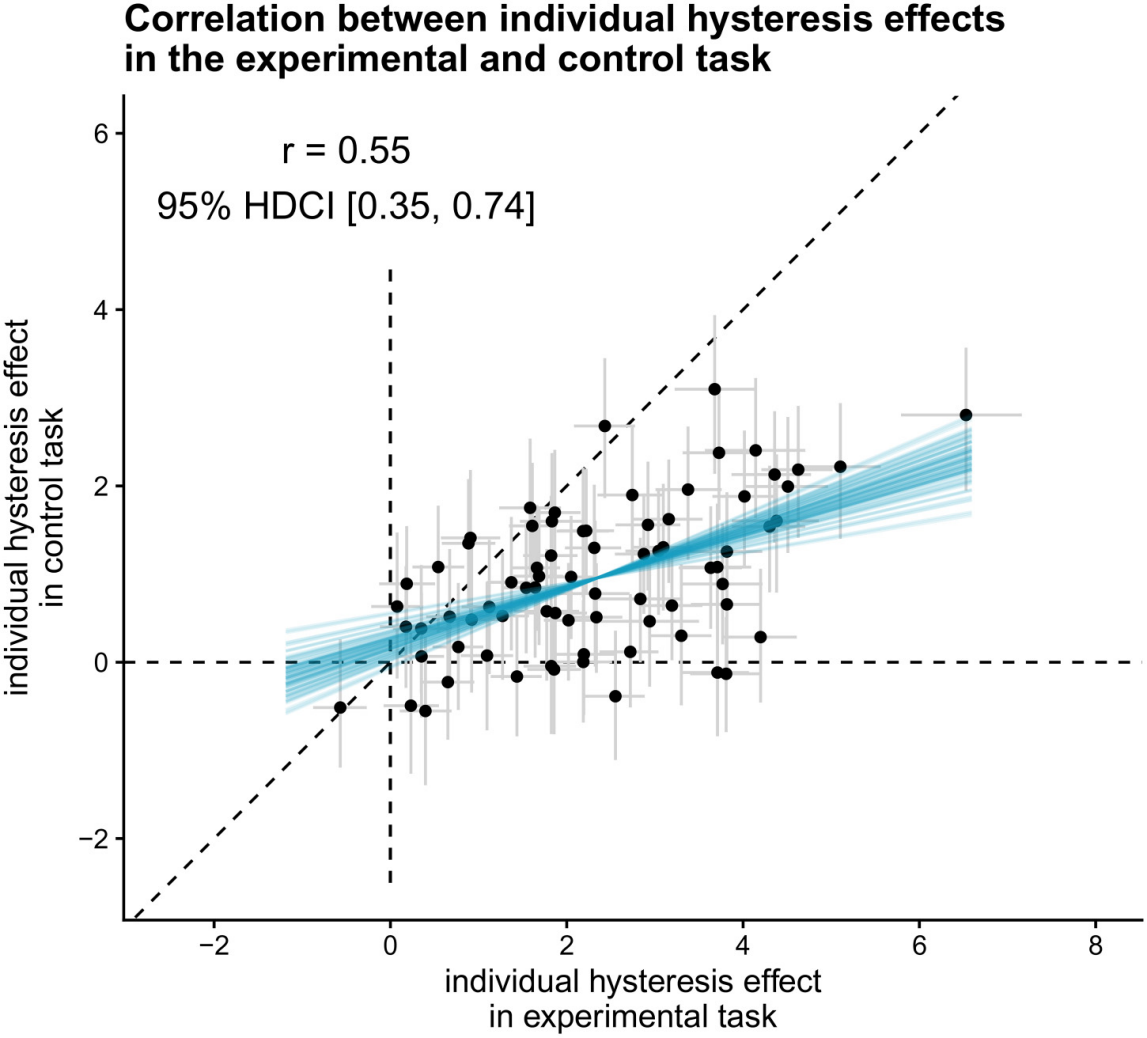
Correlation of estimated individual hysteresis effects in the experimental and control task. Mean and 80% highest density continuous intervals are shown. The diagonal black line indicates equal effects in control and experimental task. The horizontal and vertical black lines indicate a hysteresis effect of zero. The blue lines give examples of plausible correlation estimates.

The Bayes factor in favor of the model including the influence of the L1 percept for the data of the control task is $2\times 10^{29}$ . This means that the data are more likely under the model with the hysteresis effect. The 95% highest density continuous interval for the perceived L1 orientation coefficient in the model including a fixed and random hysteresis effect per participant in the control task ranges from 0.67 to 1.23. Although this means that the hysteresis effect is present in the control task, the effect is remarkably smaller than in the experimental hysteresis and adaptation task (see Figure 13). In addition, several participants do not show an irrefutably positive hysteresis effect in the control task. For an overview of the individual estimated hysteresis effects in the experimental and control task, see Figure 14.

#### Correlation with strength of absolute orientation bias? (H8)

**Figure 15.**
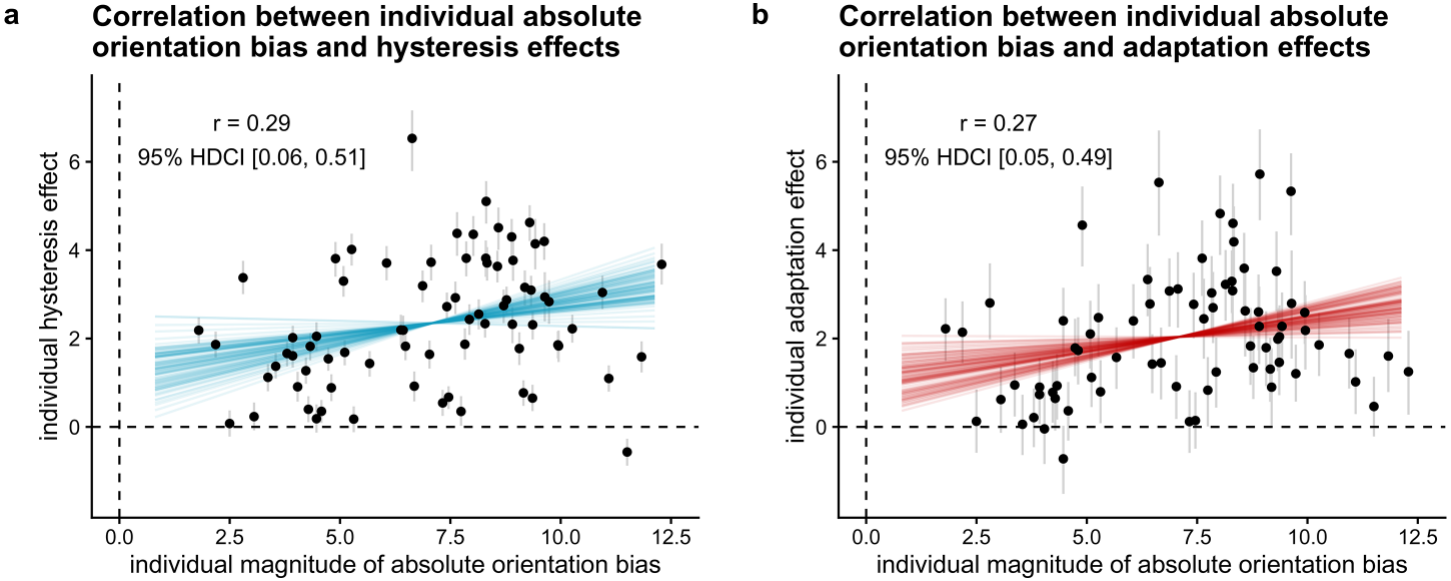
(a) Correlation between individual slopes for the effect of the aspect ratio of L1 on perceiving the 0 ${}^{\circ }$ orientation in L2 and magnitude of the absolute orientation bias in the first session. (b) Correlation between individual slopes for the effect of perceived L1 orientation on perceiving the 0 ${}^{\circ }$ orientation in L2 and magnitude of the absolute orientation bias in the first session. Mean and 80% highest density continuous intervals for the hysteresis and adaptation effects are shown. The black lines indicate an effect of zero. The colored lines give examples of plausible correlation estimates.

The direction and magnitude of the orientation bias per participant can be found in the Supplemental Appendix (see Figures A11 to A21). Figure 15 shows the correlation between the magnitude of the absolute orientation bias per individual and the individual slopes for aspect ratio and perceived L1 orientation in the model predicting perceived L2 orientation for the first session. The Bayes factor in favor of a model that assumes the true linear correlation between the individual hysteresis effects and the magnitude of the absolute orientation bias for the first session to be smaller than zero compared to a model assuming a true linear correlation larger than or equal to zero is 0.006 (inverse BF: 165.6667 ). This means that the observed data are less likely under the model assuming a negative linear correlation between individual hysteresis and absolute orientation bias effects than under the model assuming a non-negative linear correlation. The 95% highest density continuous interval for the correlation between the individual hysteresis effects and the magnitude of the absolute orientation bias ranges from 0.06 to 0.51, with a mean of 0.29. In addition to the planned analysis above, we calculated the Bayes factor in favor of a model assuming the true linear correlation between the individual hysteresis effects and the magnitude of the absolute orientation bias for the first session to be larger than zero compared to a model assuming a true linear correlation smaller than or equal to zero. This Bayes factor is equal to 165.6667 , meaning that the observed data are more likely under the model assuming a positive linear correlation between individual hysteresis and absolute orientation bias effects than under the model assuming a non-positive linear correlation.

The Bayes factor in favor of a model that assumes the true linear correlation between the individual adaptation effects and the magnitude of the absolute orientation bias for the first session to be smaller than zero compared to a model assuming a true linear correlation larger than or equal to zero is 0.0085 (inverse BF: 118.0476 ). This means that the observed data are less likely under the model assuming a negative linear correlation between individual hysteresis and absolute orientation bias effects than under the model assuming a non-negative linear correlation. The 95% highest density continuous interval for the correlation between the individual adaptation effects and the magnitude of the absolute orientation bias ranges from 0.05 to 0.49, with a mean of 0.27.

In addition to the planned analysis above, we calculated the Bayes factor in favor of a model assuming the true linear correlation between the individual adaptation effects and the magnitude of the absolute orientation bias for the first session to be smaller than zero compared to a model assuming a true linear correlation larger than or equal to zero. This Bayes factor is equal to 118.0476 , meaning that the observed data are more likely under the model assuming a positive linear correlation between individual adaptation and absolute orientation bias effects than under the model assuming a non-positive linear correlation.

Furthermore, we explored whether a quadratic model could better fit the data than a positive linear relation. For the hysteresis effect the Bayes factor of the model assuming a quadratic relation compared to a model assuming a linear relation is equal to 0.2584 (inverse BF: 3.8693 ), meaning that the observed data are less likely under the model assuming a quadratic relation between individual hysteresis and absolute orientation bias effects than under the model assuming a linear relation. For the adaptation effect the Bayes factor of the model assuming a quadratic relation compared to a model assuming a linear relation is equal to 0.4654 (inverse BF: 2.1487 ), meaning that the observed data are less likely under the model assuming a quadratic relation between individual adaptation and absolute orientation bias effects than under the model assuming a linear relation.

#### Temporal stability of individual differences in strength of hysteresis and adaptation effects? (H9)

In Supplemental Figures A5 to A7 one can find the results on logit scale on average and per participant for both sessions separately. The same figures representing the results on probability scale can be found in the Supplemental Figures A8 to A10.

The Bayes factor in favor of the model that allows for a correlation between individuals’ hysteresis effects across sessions compared to a model assuming uncorrelated individual hysteresis effects is $2\times 10^{19}$ . This means that the observed data are more likely under the model allowing for a correlation between individual hysteresis effects across sessions than under the model assuming uncorrelated effects. The Bayes factor in favor of the model that allows for a correlation between individuals’ hysteresis effects across sessions compared to a model assuming fully correlated individual hysteresis effects is $5\times 10^{83}$ . This means that the observed data are more likely under the model allowing for a correlation between individual hysteresis effects across sessions than under the model assuming fully correlated effects.

The Bayes factor in favor of a model that allows for a correlation between individuals’ adaptation effects across sessions compared to a model assuming uncorrelated individual adaptation effects is $4\times 10^{15}$ . This means that the observed data are more likely under the model allowing for a correlation between individual adaptation effects across sessions than under the model assuming uncorrelated effects. The Bayes factor in favor of the model that allows for a correlation between individuals’ adaptation effects across sessions compared to a model assuming fully correlated individual adaptation effects is $4\times 10^{-7}$ (inverse BF: $2\times 10^{6}$ ). This means that the observed data are less likely under the model allowing for a correlation between individual adaptation effects across sessions than under the model assuming fully correlated effects.

**Figure 16.**
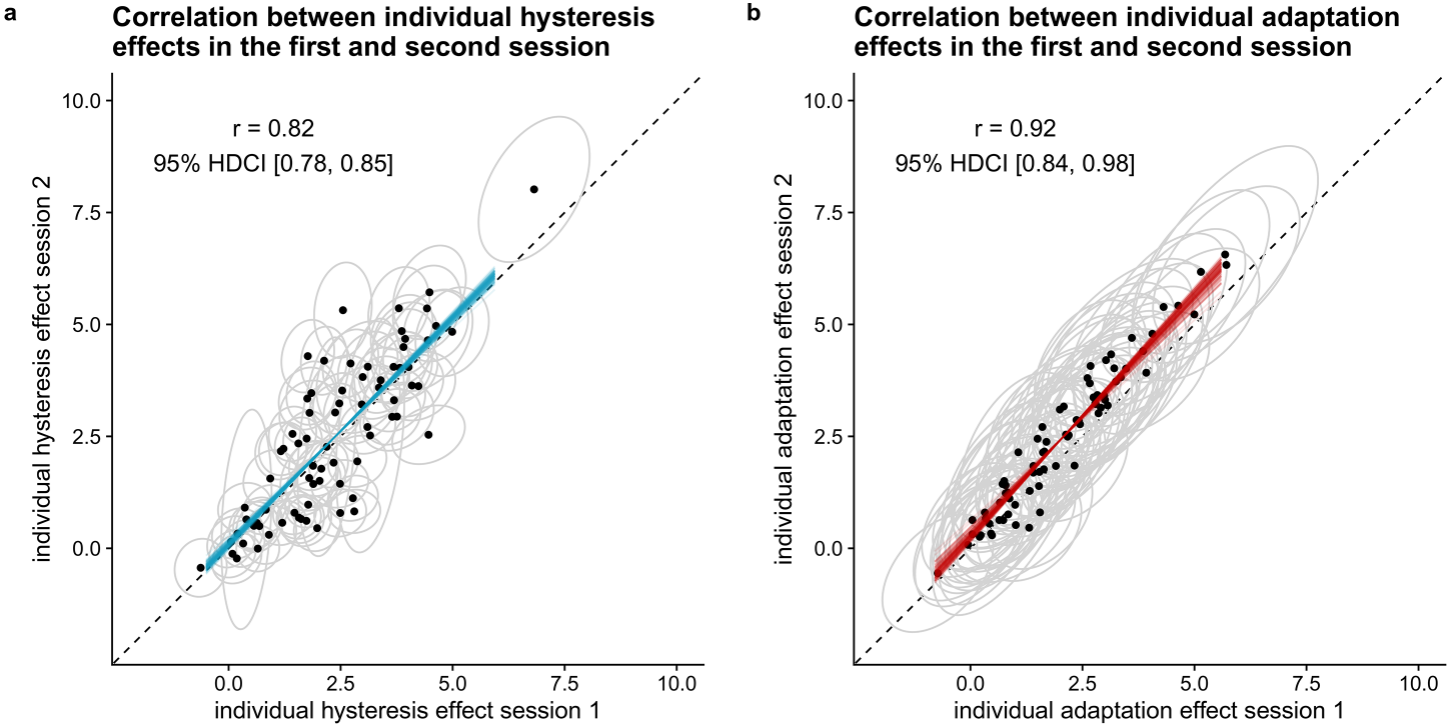
(a) Correlation between individual slopes for the effect of aspect ratio of L1 on perceiving the 0 ${}^{\circ }$ orientation in L2 in the first and the second session. (b) Correlation between individual slopes for the effect of perceived L1 orientation on perceiving the 0 ${}^{\circ }$ orientation in L2 in the first and the second session. Mean and 80% highest density continuous intervals per individual are shown. The black diagonal line indicates equally sized effects in both sessions. The colored lines give examples of plausible correlation estimates. *Note.* As the estimated correlation values shown come from a hierarchical model including both the estimates from the first and the second session, potential attenuation of the correlation as a result of noise is already taken into account.

Figure 16 shows the correlation between the first and second session individual slopes for aspect ratio and perceived L1 orientation in the model predicting perceived L2 orientation that allows for a correlation in the effects across sessions.^11^

#### Temporal stability of individual differences in strength of absolute orientation bias effects? (H10)

**Figure 17.**
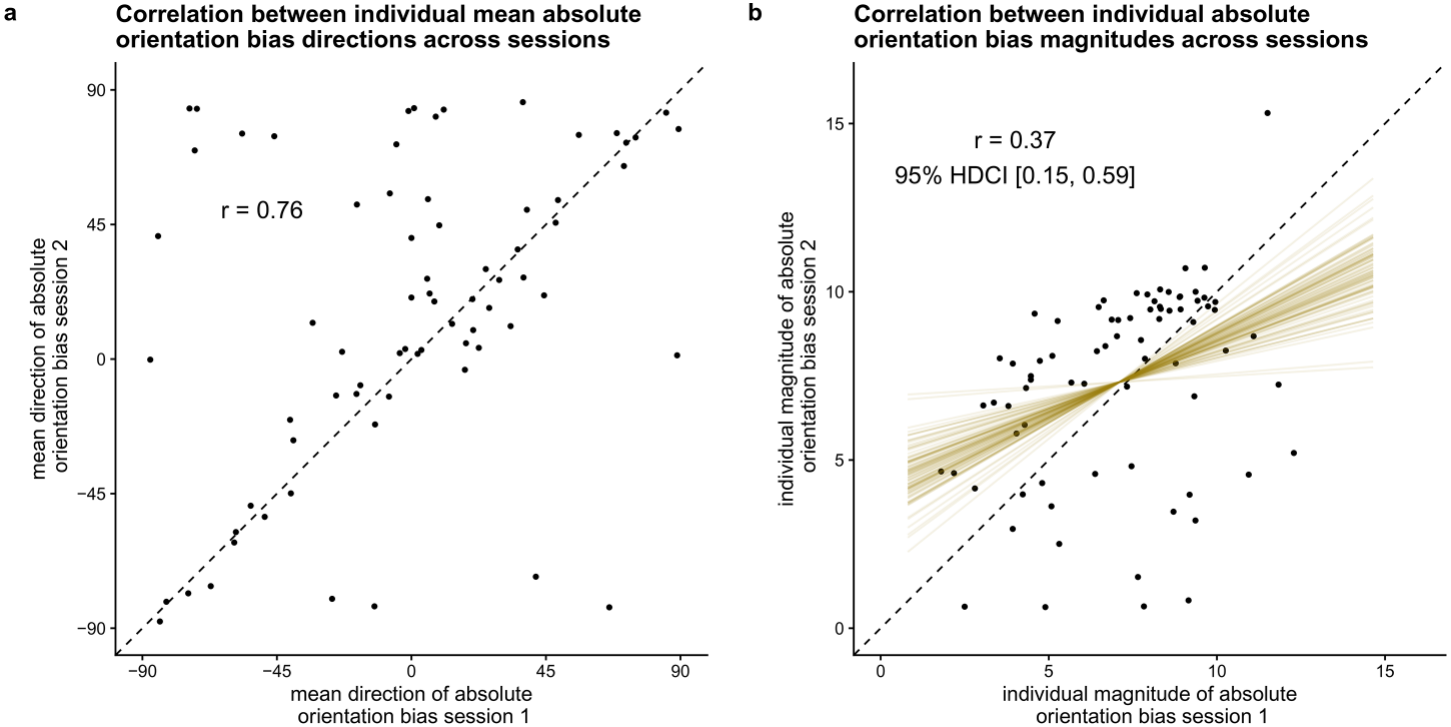
(a) Correlation between the mean direction of the absolute orientation bias in the first and second session per individual. The circular-circular correlation coefficient as defined in Mardia & Jupp (2000) is given. The black diagonal line indicates equal mean directions for the first and second session. (b) Correlation between the mean magnitude of absolute orientation bias in the first and second session per individual. The black diagonal line indicates equally sized magnitudes for the first and second session. The colored lines give examples of plausible correlation estimates.

The Bayes factor in favor of a model that assumes the true linear correlation between the magnitude of the absolute orientation biases for the first and second session to be positive compared to a model assuming a true linear correlation smaller than or equal to zero is 1999 . This means that the observed data are more likely under the model assuming a positive linear correlation between the magnitudes of the absolute orientation bias effects across sessions than under the model assuming a non-positive linear correlation. Figure 17a shows the correlation between the magnitude of the absolute orientation bias per individual in the first and second session. Figure 17b shows the correlation between the magnitude of the absolute orientation bias per individual in the first and second session.

### Additional exploratory analyses

#### Individual differences in the proximity effect?

**Figure 18.**
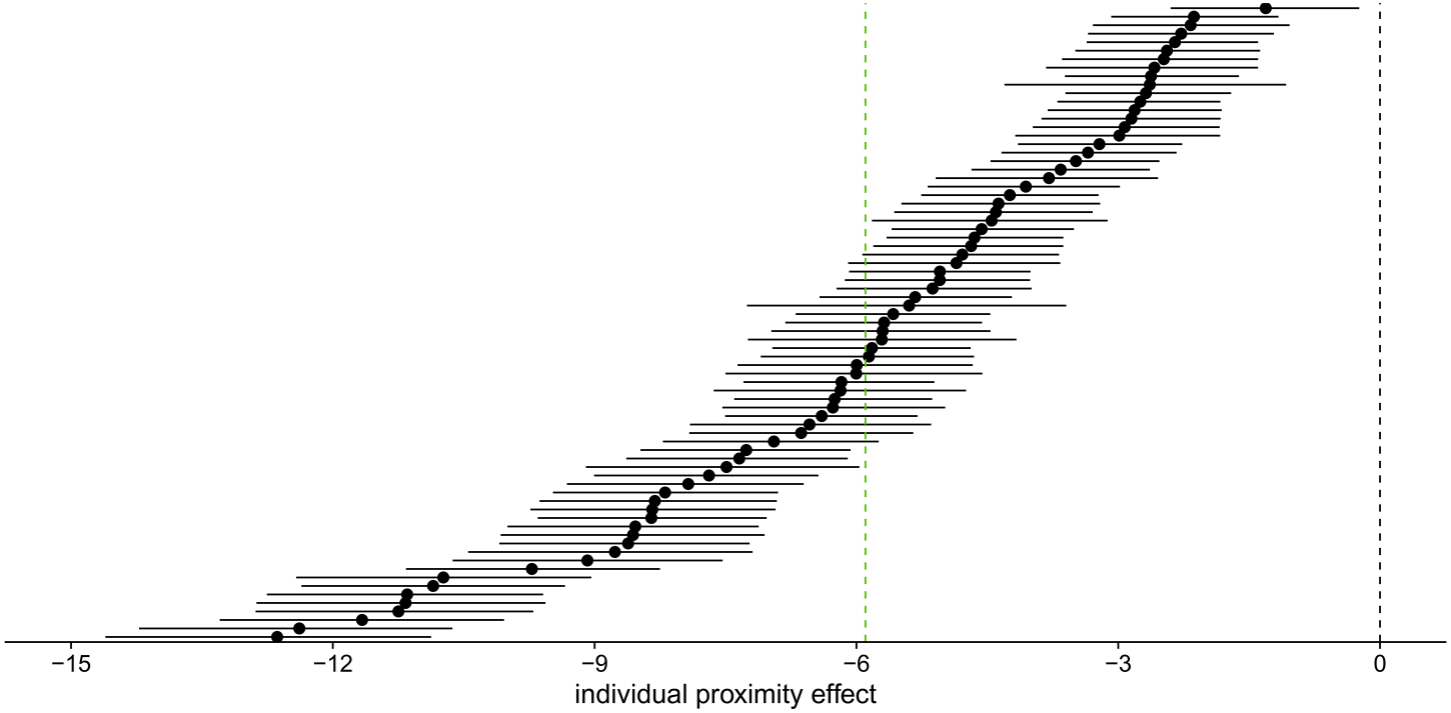
Slopes for the effect of aspect ratio on perceiving the 0 ${}^{\circ }$ orientation in L1 per participant (i.e., proximity effect). Mean and 95% highest density continuous intervals are shown. The colored line indicates the average mean effect across participants. The black line indicates a slope of zero.

We explored whether the current dataset provided formal evidence for consistent individual differences in the proximity effect, that is, the direct effect of the aspect ratio in the first lattice on which orientation was perceived in the first lattice. The Bayes factor in favor of the model with a random effect for proximity compared to the common effects model is $3\times 10^{253}$ . This means that the observed data are more likely under the unconstrained model than under the common effects model. This Bayes factor indicates that it is much more likely to assume individual differences in the proximity effect than to assume everyone to show the same effect size. Figure 18 shows the estimated individual effects of aspect ratio of L1 (i.e., proximity effect) in the model predicting perceived L1 orientation.

In addition, we explored whether the current data provided evidence for the hypothesis that everyone shows the proximity effect in the expected direction. The Bayes factor comparing the likelihood of the observed data under the negative effects model and under the unconstrained model for the proximity effect is 4.5621 . This means that the observed data are more likely under the negative effects model than under the unconstrained model. This Bayes factor indicates that it is more likely to assume that everyone shows a proximity effect in the expected direction, than to assume that not everyone shows this effect in the expected direction.

#### Temporal stability of individual proximity effects?

**Figure 19.**
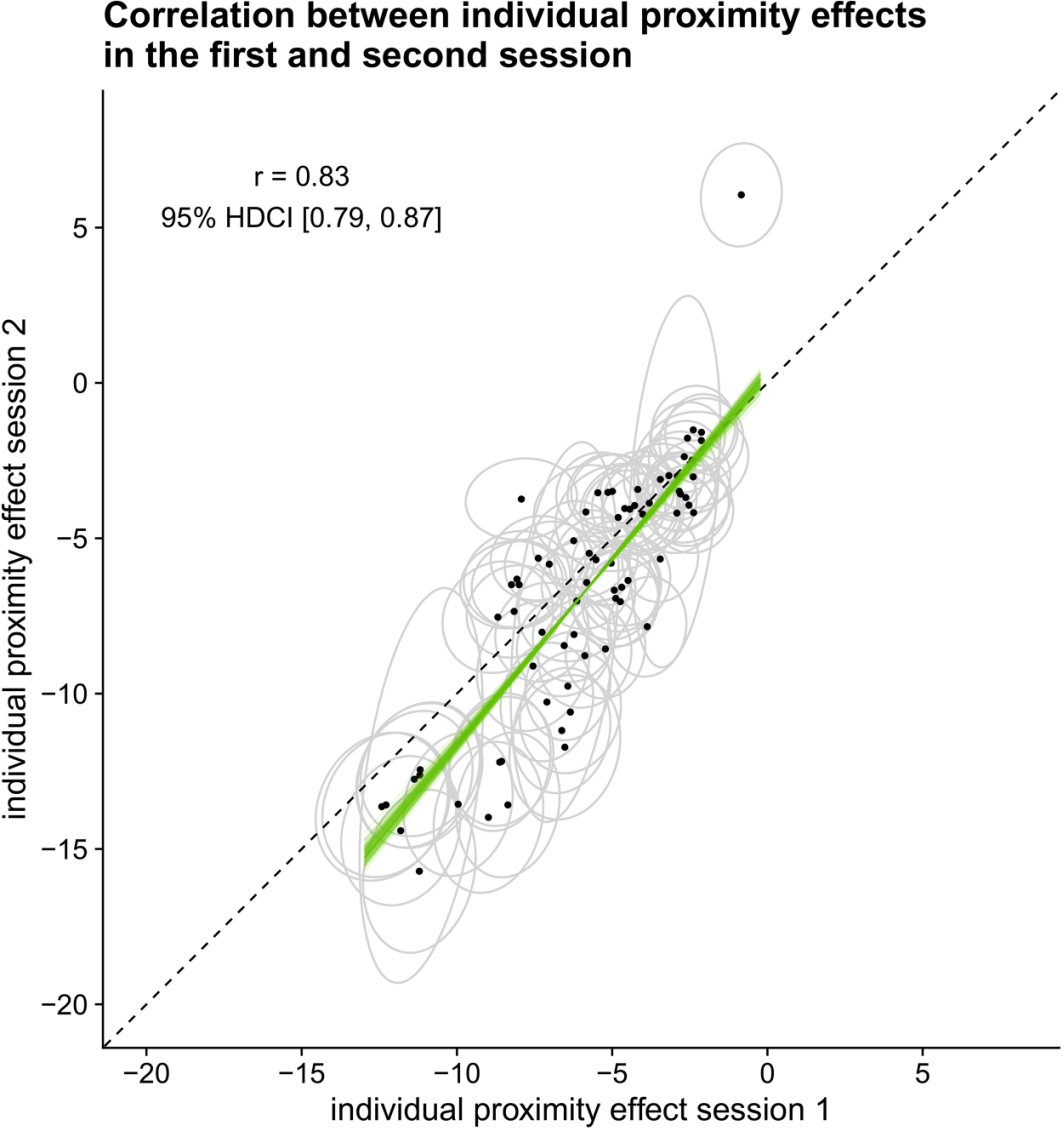
Correlation between individual slopes for the effect of aspect ratio on perceiving the 0 ${}^{\circ }$ orientation in L1 in the first and the second session. Mean and 80% highest density continuous intervals are shown. The black lines indicate a slope of zero. The green lines give examples of plausible correlation estimates. *Note.* As the estimated correlation value shown comes from a hierarchical model including both the estimates for the first and the second session, potential attenuation of the correlation as a result of noise is already taken into account.

Figure 19 shows the correlation between the first and second session individual slopes for aspect ratio in the model predicting perceived L1 orientation. It is clear from the figure that the correlation between individual proximity effects for both sessions is very high: individuals with a strong proximity effect in the first session tend to also have a strong proximity effect in the second session. In addition, except for one participant, all proximity effects are in the expected direction. The absolute size of the proximity effect per individual tended to be slightly larger in the second session.

The Bayes factor in favor of a model that allows for a correlation between individuals’ proximity effects across sessions compared to a model assuming uncorrelated individual proximity effects is $6\times 10^{13}$ . This means that the observed data are more likely under the model allowing for a correlation between individual proximity effects across sessions than under the model assuming uncorrelated effects. The Bayes factor in favor of the model that allows for a correlation between individuals’ proximity effects across sessions compared to a model assuming fully correlated individual proximity effects is $4\times 10^{63}$ . This means that the observed data are more likely under the model allowing for a correlation between individual proximity effects across sessions than under the model assuming fully correlated effects.

#### Relation between individual proximity effects and context effects?

Given that individual proximity effects and temporal attractive and repulsive context effects (i.e., hysteresis and adaptation) show very stable across sessions, we were interested in the relation between the direct effect of aspect ratio on (more often) perceiving the 0 ${}^{\circ }$ orientation in the first lattice (i.e., proximity effect) and the indirect effect of aspect ratio on (less often) perceiving the 0 ${}^{\circ }$ orientation in the second lattice (i.e., adaptation effect). In addition, we computed the correlation between individual proximity effect and hysteresis effects.

**Figure 20.**
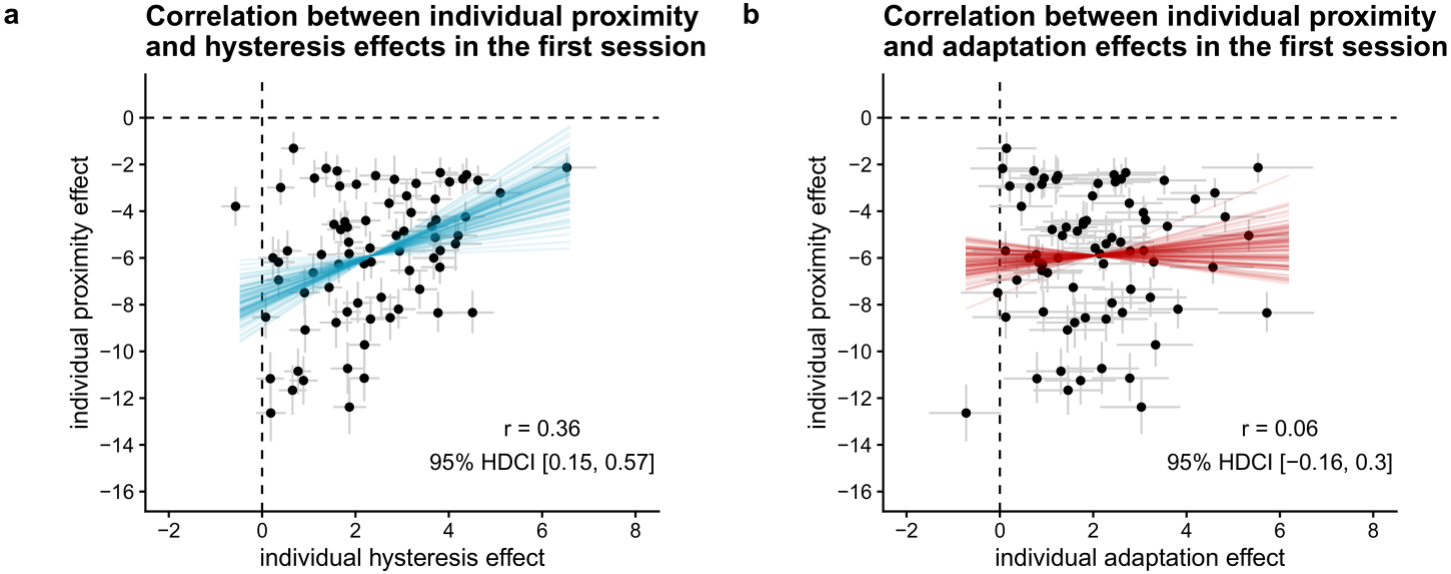
(a) Correlation of estimated individual hysteresis effects concerning the second lattice with estimated individual proximity effects concerning the first lattice. (b) Correlation of estimated individual adaptation effects concerning the second lattice with estimated individual proximity effects concerning the first lattice. Mean and 80% highest density continuous intervals are shown. The black lines indicate a slope of zero. The colored lines give examples of plausible correlation estimates.

Figure 20 shows the correlation between the proximity effect and the temporal context effects per individual. The correlation of individual proximity effects and individual adaptation effects was negligible (see Figure 20b): knowing the size of an individual’s proximity effect does not tell us much about the size of an individual’s adaptation effect. The size of individual proximity effects and individual hysteresis effects was negatively correlated (see Figure 20a),^12^ but also the differences in variance across the range of hysteresis effects needs to be taken into account: whereas individuals with a strong influence of their previous percept on their current percept have a larger probability of having a small proximity effect, individuals with a small hysteresis effect do not necessarily have a strong direct effect of aspect ratio on their percept (i.e., a strong proximity effect).

#### Relation of proportion of non-dominant responses, left-right response bias, and context effects?

**Figure 21.**
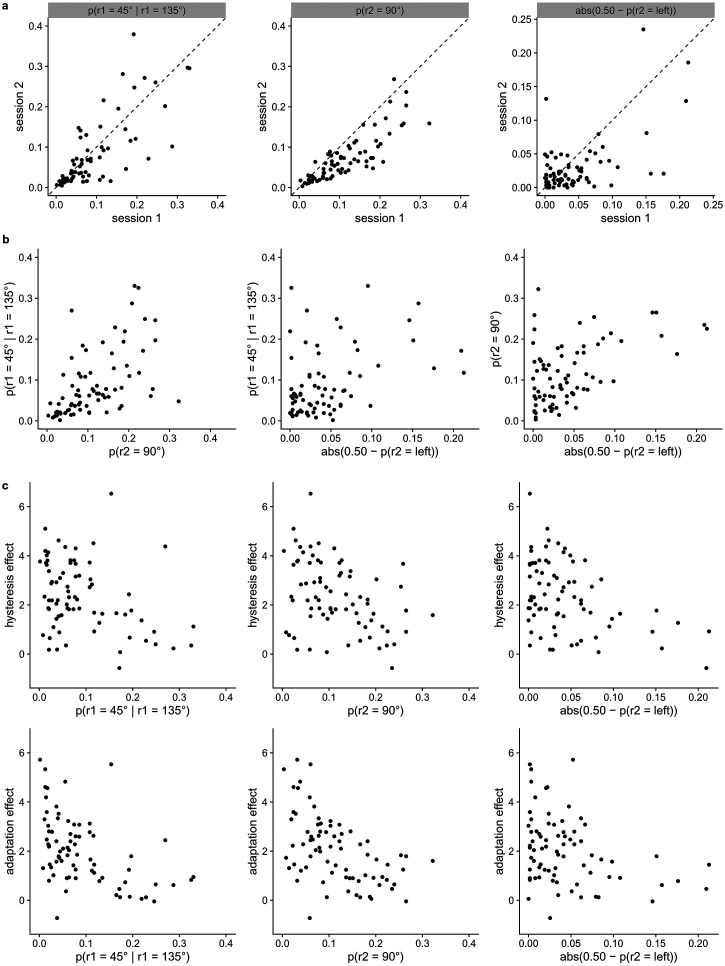
(a) Correlation across sessions for the probability of diagonal L1 responses, the probability of 90 ${}^{\circ }$ responses for L2, and the absolute difference from chance level in selecting a left or right response option for L2 per individual. The black diagonal lines indicate equal probabilities. (b) Correlations between the probability of diagonal L1 responses, the probability of 90 ${}^{\circ }$ responses for L2, and the absolute difference from chance level in selecting a left or right response option for L2 per individual. (c) Correlation of estimated individual hysteresis and adaptation effects concerning the second lattice with individual probabilities of diagonal L1 responses, probabilities of 90 ${}^{\circ }$ responses for L2, and individual magnitudes of the left-right response asymmetry to the second lattice.

We explored the relation of an individual’s probability to give non-dominant responses to the first and second lattice as well as their asymmetry of choosing a response option for the second lattice requiring a response with the left or the right hand with the size of the individual’s attractive and repulsive context effects. First, the probability of giving a diagonal response in the first lattice was positively correlated across sessions, as was the probability of giving (impossible) 90 ${}^{\circ }$ responses to the second lattice (see Figure 21a). Although for the magnitude of the left-right response asymmetry most participants showed only slight deviations from chance, participants with strong deviations from chance level did at least sometimes show this deviation in both sessions (see Figure 21a). Furthermore, the probability of giving a diagonal response in the first lattice correlated considerably with the probability of giving an (impossible) 90 ${}^{\circ }$ response to the second lattice as well as the difference in proportion of left and right responses to the second lattice compared to chance level (see Figure 21b). When correlating an indviduals’ probability of giving non-dominant responses to their estimated hysteresis and adaptation effects, a consistent pattern arises: Whereas participants with a small number of non-dominant responses vary widely in the size of their hysteresis and adaptation effects, having more non-dominant responses seems to relate to smaller hysteresis and adaptation effects. A similar pattern is visible for the relation between the size of the left-right response asymmetry to the second lattice and the size of the hysteresis and adaptation effects: individuals with a large left–right response asymmetry typically have small hysteresis and adaptation effects, whereas the range of possible hysteresis and adaptation effect sizes is much wider for individuals with only a small left-right response asymmetry.

## Discussion and conclusion

With this Registered Report, we investigated (a) whether we could replicate the average attractive and repulsive context effects found in the perception of multistable dot lattices, (b) whether consistent differences in the size of these effects could be found between individuals, and (c) whether every individual showed both effects in the expected direction. In addition, we investigated (d) whether individual differences in both context effects were positively correlated, (e) whether the hysteresis effect could be ascribed to perceptual or decisional causes, (f) whether individual differences in both context effects were correlated with the strength of individual’s absolute orientation biases, and (g) whether individual differences in attractive and repulsive context effects as well as in the magnitude of absolute orientation biases were stable across time. In addition, we exploratorily investigated (h) whether consistent differences in the size of the proximity effect exist between individuals and whether every individual showed a proximity effect in the expected direction, (i) whether individual differences in the proximity effect were stable across time, (j) whether individual differences in the proximity effect correlated with individual differences in the hysteresis and adaptation effects, and (k) how individual differences in proportions of non-dominant responses and left-right motor response biases related to individual differences in the hysteresis and adaptation effects.

### Summary of the main findings

#### Average results on attractive and repulsive context effects replicate (H1-3)

When looking at the results averaged across participants (see Figures 8 and A1), we successfully replicated the attractive effect of the previous percept (i.e., perceived L1 orientation; cf. H1) and the repulsive effect of the previous stimulus (i.e., aspect ratio of L1; cf. H2) on the current percept (i.e., perceived L2 orientation), as well as the absence of evidence for an interaction between both effects (cf. H3). The Bayes factors, indicating how to update our belief in one model relative to the alternative model, were strongly in favor of including both the hysteresis and the adaptation effect. The Bayes factor comparing a model with and without interaction between the hysteresis and the adaptation effect was in favor of the model without the interaction. This study thus fully replicates the average results from Schwiedrzik et al. (2014) and Gepshtein and Kubovy (2005).

#### Consistent individual differences exist in the magnitude of attractive and repulsive context effects (H4)

The results averaged across participants do not tell the complete story, however: finding evidence for an average effect does not guarantee individuals’ true effects to be of the same size or in the same direction. When inspecting individual results for the experimental task, it is clear that individuals differ in how strongly the aspect ratio of the first lattice and their percept of the first lattice influence their percept of the second lattice (see Figures 9b and A2b). Bayes factors strongly preferred the unconstrained models above the common effects models, providing evidence for true individual differences in both the size of the hysteresis effect and the size of the adaptation effect.

This evidence for true individual differences in the size of attractive and repulsive temporal context effects is of theoretical importance: It tells us that individuals cannot only differ in their perception because of differences in previously encountered stimuli and percepts, but can also differ in the way context is incorporated into perception: different individuals use context information concerning the previous stimulus and the previous percept to a different extent. In other words, even when individuals would have exactly the same stimulus history and perceptual history, they could still differ in what they perceive due to differential use of the stimulus history and perceptual history when forming a new percept.

#### Not everyone clearly shows attractive and repulsive context effects (H5)

As the Bayes factors concerning H5 indicated a preference for the unconstrained models above the positive effects models, these results indicate that neither do all individuals show a clear attractive effect of the previous percept, nor do all individuals show a clear repulsive effect of the previous stimulus. Nevertheless, *almost* everyone showed clear attractive and repulsive context effects in the expected direction. The number of participants with an estimated true non-positive hysteresis effect and/or an estimated true non-positive adaptation effect was very low (see Figure 11). Importantly, also individuals with an estimated non-positive hysteresis or adaptation effect showed consistency across sessions, indicating that the non-positive estimate was not a strange oddity (see Figure 16). In addition, we explored possible differences between the participants with somewhat extreme results and the other participants but found no consistent differences regarding the demographics (e.g., age) or the number of days in-between the two test sessions.

Even though the current findings indicate that not *everyone* shows hysteresis and adaptation effects in the expected direction, the results do correspond well with the results from the reanalysis of the data collected by Schwiedrzik et al. (2014). As having a true non-positive hysteresis and/or adaptation effect seems to be very rare, it is reasonable that no non-positive effects were found in that reanalysis, which only included 27 participants. The current sample thus gives a more complete and nuanced picture on the range of plausible hysteresis and adaptation effects, but also confirms that *almost* everyone shows attractive and repulsive context effects in the expected direction.

Also the finding that almost everyone shows an attractive effect of the previous percept and a repulsive effect of the previous stimulus evidence has theoretical implications. Future research can aim to shed light on why these context effects show this direction for almost all individuals. Nevertheless, it is equally important for future research to take the full range of individual differences present into account when attempting to explain these effects, including the presence of at least some individuals with a true effect in the opposite direction. In addition, it is important to investigate whether existing models of attractive and repulsive temporal context effects can incorporate the variability found, as a good model should not only be able to predict the mean, but also plausible variation in the effect’s size and direction.

#### At least a common factor affecting both hysteresis and adaptation (H6)

The results indicate a strong positive correlation between estimated individual hysteresis and adaptation effects. This positive correlation thus suggests that there may at least be a common factor affecting the processes underlying both effects. It is unclear however what exactly may explain this high positive correlation between the magnitude of both effects. One way to understand the high positive correlation is that hysteresis and adaptation are both context effects, and that individuals can be contrasted based on how strongly they are influenced by context in general versus how strongly they are influenced by the direct perceptual evidence present. The current results can thus not exclude the hypothesis that both context effects stem from the same underlying mechanism, and thus also seem to support the hypothesis that both effects have at least some common underlying factor. This conclusion is similar to the conclusion of Snyder et al. (2019), who found a positive correlation between individual differences in inhibition across contrast and assimilation tasks, indicating at least some common factor influencing the size of both context effects.

#### The attractive context effect is partially percept-related, partially decision-related (H7)

As the experimental task could not distinguish between a perceptual or a post-perceptual nature of the hysteresis effect, a control task was included in which perceptual factors were ruled out. Even in the control task there was an attractive effect of the previous response, although this effect was considerably smaller than in the experimental task (and absent for at least some participants). This suggests that the attractive context effect is neither solely percept-related (i.e., dependent on actually perceiving the orientation in question), nor solely decision-related (i.e., dependent on the choice for a specific orientation without involving perception). In this way, the results nuance earlier perspectives stating ‘‘serial dependence’’ to be either a fully percept-related or a fully decision-related effect (e.g., Bosch et al., 2020; Carter et al., 2014; Cicchini et al., 2017; Fritsche et al., 2017; Manassi et al., 2018; Pascucci et al., 2019; Schwiedrzik et al., 2018). Also, individuals seem to differ in the extent to which their hysteresis effect is percept- or decision-related: several participants do not show an indisputably positive hysteresis effect in the control task, indicating a more perceptual basis for their hysteresis effect. The difference in the size of the hysteresis effect between the experimental and the control task could potentially also be interpreted as related to decision confidence^13^ : biases based on past decisions could be expected to be larger in cases in which decision confidence was higher because the past decision was based on perceptual evidence, compared to cases in which no perceptual evidence was present. Even when following this interpretation in terms of decision confidence however, the actual reason for the difference stays perceptual.

#### The magnitude of individual’s absolute orientation bias and their attractive and repulsive context effects correlate positively (H8)

In contrast to our expectation, the magnitude of individual’s absolute orientation bias did correlate positively rather than negatively with the strength of individual’s attractive and repulsive context effects. It has to be noted, however, that Bayes factors provide an evidence ratio between two specific models, in this case being the model assuming a negative linear correlation versus the model assuming a non-negative linear correlation. Consequently, a high Bayes factor does not guarantee the winning model to provide a good fit to the observed data. From the scatterplots (see Figure 15), it is unclear whether a linear model provides a good fit for the data. Although we tested whether a quadratic model could better predict the data pattern, relative evidence for a model assuming an inverted U-shaped curve compared to a linear model was slightly in favor of the linear model.

The results provide slight evidence for a positive relation between the size of individuals’ absolute orientation bias and their hysteresis and adaptation effects. This could be interpreted as a slight positive relation between different types of biases, but the actual reason for this positive correlation is unclear. Furthermore, although the linear model was preferred over the quadratic model, it is unclear whether the linear model does provide a good fit for the data.

#### Individual differences in attractive and repulsive context effects are stable over time (H9)

Individual differences in both attractive and repulsive context effects in the used multistable dot lattice paradigm show to be very stable, at least across a period of 7 to 14 days. For hysteresis (i.e., attractive context effect of the previous percept), the winning model was the model assuming a correlation but no full correlation between the hysteresis effects in both sessions. For adaptation (i.e., repulsive context effect of the previous stimulus shown), the winning model was the model assuming a full correlation between the adaptation effects in both sessions. These results indicate that individual differences in the size of hysteresis and adaptation effects are reliable indices of individual differences across time, at least in the current multistable dot lattices paradigm, and it can be useful to investigate their relations with other individual difference factors as well as with estimates of individual hysteresis and adaptation effects assessed using different stimuli and tasks. Our results indicating a strong but not full correlation of individual differences in attractive context effects are in line with the results of Kondo et al. (2022), who found a high degree of consistency within individual observers when assessing attractive serial dependence in orientation perception.

#### Differences in the magnitude of individual’s absolute orientation bias are stable over time (H10)

Although a large number of the participants in the sample showed a very consistent mean absolute orientation bias strength, this was not the case for all participants. Post-hoc analyses showed that the mean direction of the absolute orientation bias stayed relatively stable across time, at least for most participants (see Figure 17).

#### Consistent individual differences exist in the magnitude of proximity effects, and everyone shows the proximity effect

When exploring individual results for the proximity effect, it became clear that individuals differ in how strongly the aspect ratio of the first lattice influences their percept of the first lattice (see Figures 9a and A2a). The Bayes factor indicated a strong preference for the unconstrained model above the common effects model, indicating that there is evidence for true individual differences in the size of the proximity effect. When exploring whether everyone shows the proximity effect in the expected direction, the Bayes factor indicated a preference for the negative effects model above the unconstrained model. This supports the idea that all individuals show a proximity effect in the expected direction.

#### Individual differences in the proximity effect are stable over time

Post-hoc analyses indicated high stability for individual differences in how strongly participants are affected by proximity in their percept of the first lattice (see Figure 19). The absolute size of the proximity effect per individual tended to be slightly larger in the second session. Furthermore, the size of individuals’ proximity effects was negatively related to the size of individuals’ hysteresis effects: the larger an individual’s hysteresis effect, the smaller the range of plausible values for their proximity effect, and the smaller their proximity effect. To the contrary, the size of an individual’s proximity effect was uncorrelated to the size of their adaptation effect in the current sample (see Figure 20). Although at first sight proximity seems to be differentially related to hysteresis and adaptation, this result should be replicated and further investigated before making firm conclusions. In case the differential relationship of hysteresis and adaptation with proximity holds, this would suggest a dissociation between hysteresis and adaptation.

#### Proportion of non-dominant responses and left-right response bias relate negatively to attractive and repulsive context effects

Post-hoc visualizations (see Figure 21) indicated stable individual differences in the probability of choosing a non-dominant response option for the percept of both the first and the second lattice. Although most participants showed only slight deviations from chance, participants with strong deviations from chance level when choosing a response option for the second lattice requiring a response with the left or the right hand did at least sometimes show this deviation in both sessions. High probabilities of choosing non-dominant responses related negatively to hysteresis and adaptation effects: Whereas participants with a small number of non-dominant responses vary widely in the size of their hysteresis and adaptation effects, having more non-dominant responses seems to be related to smaller hysteresis and adaptation effects. In addition, individuals with a large left-right asymmetry in their L2 responses showed smaller hysteresis and adaptation effects. These exploratory results may indicate that more attentive participants show a larger range of possible hysteresis and adaptation effect sizes, whereas less attentive participants have smaller effects of previous percept and previous stimulus on the current percept.

### Suggestions for future research

#### Factors correlating with individual differences in hysteresis and adaptation effects

By providing strong empirical evidence for the existence of consistent differences in individuals’ true attractive and repulsive context effects, this work can form a starting point for future work exploring potential factors to explain these individual differences. Some earlier research already suggested relations between the reduced use or differential weighting of stimulus history and some clinical conditions, using different tasks (not distinguishing between stimulus history and perceptual history). Stein et al. (2020) found that a reduced influence of previous stimuli on working memory contents in patients with schizophrenia and anti-NMDAR encephalitis. Lieder et al. (2019) showed a differential use of previous sensory information in individuals with autism and dyslexia: whereas individuals with autism relied more on longer-term statistics, individuals with dyslexia relied more on information about the immediate past. Future research can explore relations with the multistable dot lattices paradigm in different clinical conditions, but can also explore other potential correlates of individual differences in the use of stimulus history and perceptual history across tasks (e.g., personality differences). In addition, special attention needs to be paid to the individuals showing negative hysteresis and adaptation effects. Future research can investigate what underlies the unexpected direction of the effects in these individuals, and needs to take the existence of those negative effects into account whenever attempting to explain individual differences.

#### Explaining the strong positive correlation between hysteresis and adaptation effects

As the current study found evidence for a strong positive correlation between the size of attractive and repulsive context effects across individuals, future research can further investigate the source of this strong positive correlation. Especially theoretical and modeling work, in combination with empirical validation, can be useful to get a more concrete insight in the process underlying this positive correlation.

#### Processes underlying hysteresis and adaptation effects

Why does almost everyone show attractive effects of the previous percept and decision, and repulsive effects of the previous stimulus? Also regarding this question, future theoretical work and modeling efforts, in combination with empirical validation, can contribute to a better understanding of the underlying processes. In addition, the existence of true individual differences in the size and direction of an effect has consequences for the models and theories aiming to explain or predict these effects: It is important to verify whether existing models and theories can reproduce or explain the range of variability found in the effects’ size and direction across individuals, as a good model should not predict the mean alone, but also plausible variation in the effect’s size and direction.

#### Individual differences in the presence of an interaction effect between hysteresis and adaptation

Although the results from our study suggest the model without an interaction effect between hysteresis and adaptation to be preferred above the model including an interaction, individual differences seem to exist in the presence of this interaction, with most participants not showing an interaction, but some clearly showing an interaction between the two (e.g., participants 011, 029, and 081 in the current dataset). Future research could investigate whether it is worth including an interaction effect for a subsample of the participants to more accurately estimate their context effects.

#### Generalizability of individual differences in hysteresis and adaptation effects to different stimuli and tasks

The current results indicate highly stable individual differences in attractive and repulsive context effects across time, at least when assessed using this specific multistable dot lattices paradigm. Future research needs to examine whether the stable individual differences in attractive and repulsive context effects found in the current task correlate with similar individual differences assessed using different tasks or stimuli.

#### Further disentangling hysteresis as a perceptual and a decisional effect

The current study finds support for a partially perceptual and partially decisional nature of the attractive context effect. In addition, the current results suggest that individuals may differ in the extent to which their attractive context effect is related to perception or decision: Whereas some individual’s effects are almost equal in size in both control and experimental task (i.e., indicating a mainly decisional nature of the effect), most individuals show a considerably smaller effect in the control task (i.e., indicating a combination of perceptual and decisional nature), and some individuals do show no evidence for a hysteresis effect in the control task (i.e., hinting at a fully perceptual nature). Future studies can focus more specifically on individual differences in the nature of these effects, and in that way disentangle individuals’ perception- and decision-related attractive context effects.

#### Further disentangling stimulus-, percept-, decision-, and response-related effects

One of the advantages of using the current multistable dot lattices paradigm is the explicit distinction that can be made between effects of the previous stimulus and those of the previous percept/decision/response. We believe this distinction is crucial to enhance clarity in the research literature. Although previous work has often distinguished between stimulus and decision or stimulus and response, any non-stimulus related effect has typically been reported as ‘‘postperceptual.’’ We want to clarify that the fact that an effect is non-stimulus related does not directly imply that the effect is postperceptual, but that the effect could be the consequence of the way the stimulus was experienced (i.e., the percept), rather than being put away as purely decisional or response-related. That said, explicitly studying the distinct contributions of all different factors, including stimulus, percept, decision, and motor response, is a relevant topic for future research.

#### Replicating and explaining the relation between absolute orientation biases and hysteresis and adaptation effects

The results concerning the relationship between absolute orientation bias and hysteresis and adaptation effects found in the current study were ambiguous and require further study and replication by other researchers. Given that the current results replicate, it is worth explaining how the complex relation between these individual difference factors emerges.

### Take home message

In this study, we replicated the average attractive effect of a previous percept on the current percept and the repulsive effect of previously presented stimulus evidence on the current percept. Large individual differences in the size of these attractive and repulsive context effects exist, however, and these individual differences are consistent across timepoints (one to two weeks apart). Although *almost everyone* shows both effects in the expected direction, *not every* single individual does. Furthermore, individual differences in the size of attractive and repulsive context effects are strongly positively correlated, suggesting at least a common factor influencing the processes underlying both effects. In addition, the attractive context effect is shown to be partially percept-related and partially decision-related, nuancing earlier debates on the origin of this effect.

In sum, the study provides insight in how individuals differ in how they combine previous input and experience with current input in their perception, and more generally, this tells us that different individuals can perceive identical stimuli differently, even within a similar context.

## Open and reproducible practices statement

This manuscript was written in R Markdown using the papaja package (Aust & Barth, 2020) with code for data analysis integrated into the text. The data, materials, and analysis and manuscript code for the experiment are available at https://doi.org/10.17605/osf.io/wae6k. The preregistration for this experiment is available at https://doi.org/10.17605/osf.io/qmgca.

## Supplemental Material

sj-pdf-1-ipe-10.1177_20416695221109300 - Supplemental material for Same stimulus, same temporal context, different percept? Individual differences in hysteresis and adaptation when perceiving multistable dot lattices Click here for additional data file. Supplemental material, sj-pdf-1-ipe-10.1177_20416695221109300 for Same stimulus, same temporal context, different percept? Individual differences in hysteresis and adaptation when perceiving multistable dot lattices by Eline Van Geert, Pieter Moors, Julia Haaf, and Johan Wagemans in i-Perception

## Notes

1. Aspect ratio is sometimes defined as |*a*|/|*b*| with |*a*| and |*b*| with a fixed orientation but changing length (e.g., Schwiedrzik et al., 2014), and sometimes as |*b*|/|*a*|, where |*a*| varies in orientation but is always the orientation with the shortest inter-dot distance (e.g., Gepshtein & Kubovy, 2005). Here we will use |*a*|/|*b*| with |*a*| and |*b*| with a fixed orientation but changing length.

2. The results from this reanalysis are available on the Open Science Framework: https://osf.io/xa5ut/

3. As it was not feasible to use sequential sampling based on all hypotheses, we focused our sampling plan on hypotheses 4 and 5. We view these hypotheses as our main targets for this study. As we believe the conclusiveness of our results depends mostly on the informativeness of the Bayes factors for hypotheses 4 and 5, a Bayes factor less informative than 6 or 1/6 for any of the additional hypotheses would not be as problematic. At least for hypotheses 1-2 and for hypothesis 6, our reanalysis of the data from Schwiedrzik et al. (2014; with our exclusion criteria, *N* = 27) indicated that this sample size was enough to get a Bayes factor above 6. For hypothesis 7–10, we could not test this as Schwiedrzik et al. (2014) did not include a control task, an absolute orientation bias task, or two separate sessions.

4. More specifically, the Bayes factor comparing the likelihood of the observed data under the positive effects model and under the unconstrained model for the percept of L1 (i.e., hysteresis effect) went from 3.04 (in favor of positive effects model) to 0.02 (conclusive and in favor of the unconstrained model) when going from 50 to 55 participants. Also the Bayes factor comparing the likelihood of the observed data under the positive effects model and under the unconstrained model for the aspect ratio of L1 (i.e., adaptation effect) suddenly became conclusive going from 50 to 55 participants, albeit without direction change: from 0.55 to 0.05.

5. We thank the authors for sharing their code with us.

6. For two participants the sessions were more than 14 days apart (i.e., 17 and 21 days), as the second session had to be rescheduled because of medical reasons. Nevertheless, we included their data for the second session in the final analysis.

7. For an overview of all R packages used, see the Supplemental Appendix.

8. We thank the authors for sharing their data with us for this purpose. The results from this reanalysis are available on the Open Science Framework: https://osf.io/xa5ut/

9. For the control task, approximately 50% of trials are excluded, which is to be expected given that no orientation was visible in the first lattice (and all responses should be chosen approximately equally).

10. Gepshtein and Kubovy (2005) and Schwiedrzik et al. (2014) used an alternative calculation of the logodds (cf. p. 489 in Gepshtein & Kubovy, 2005). We provide these alternative visualizations in the Supplemental Appendix to facilitate the comparison to these papers.

11. Different from what we had preregistered, we do not show the mean and 95% HDCI for the correlation in the winning model for the adaptation effect, as the winning model was the model assuming fully correlated effects across sessions and thus assumes a correlation equal to 1.

12. Note that a more negative proximity effect indicates a stronger effect of proximity on perception. Consequently, the positive slope in Figure 20a implies a negative correlation.

13. We thank an anonymous reviewer of our First Stage Registered Report for this suggestion.

## References

[bibr1-20416695221109300] Abrahamyan A. Silva L. L. Dakin S. C. Carandini M. Gardner J. L. (2016). Adaptable history biases in human perceptual decisions. Proceedings of the National Academy of Sciences of the U.S.A., 113, E3548–E3557. 10.1073/pnas.1518786113 PMC4922170 27330086

[bibr2-20416695221109300] Appelle S. (1972). Perception and discrimination as a function of stimulus orientation: The “oblique effect” in man and animals. Psychological Bulletin, 78, 266–278. 10.1037/h0033117 4562947

[bibr3-20416695221109300] Aust F. Barth M. (2020). *papaja: Create APA manuscripts with R Markdown*. Retrieved from https://github.com/crsh/papaja.

[bibr4-20416695221109300] Bosch E. Fritsche M. Ehinger B. V. de Lange F. P. (2020). Opposite effects of choice history and evidence history resolve a paradox of sequential choice bias. Journal of Vision, 20, 9–9. 10.1167/jov.20.12.9 PMC7683864 33211062

[bibr5-20416695221109300] Brascamp J. W. Knapen T. H. J. Kanai R. Noest A. J. van Ee R. van den Berg A. V. (2008). Multi-timescale perceptual history resolves visual ambiguity. PloS one, 3, e1497. 10.1371/journal.pone.0001497 18231584 PMC2204053

[bibr6-20416695221109300] Bürkner P.-C. (2017). brms: An R package for Bayesian multilevel models using Stan. Journal of Statistical Software, 80, 1–28. 10.18637/jss.v080.i01

[bibr7-20416695221109300] Bürkner P.-C. (2018). Advanced Bayesian multilevel modeling with the R package brms. The R Journal, 10, 395–411.

[bibr8-20416695221109300] Carter O. Snyder J. S. Fung S. Rubin N. (2014). Using ambiguous plaid stimuli to investigate the influence of immediate prior experience on perception. Attention, Perception, & Psychophysics, 76, 133–147. 10.3758/s13414-013-0547-5 24101343

[bibr9-20416695221109300] Cicchini G. M. Mikellidou K. Burr D. (2017). Serial dependencies act directly on perception. Journal of Vision, 17, 6. 10.1167/17.14.6 29209696

[bibr10-20416695221109300] Claessens P. M. E. Wagemans J. (2008). A Bayesian framework for cue integration in multistable grouping: Proximity, collinearity, and orientation priors in zigzag lattices. Journal of Vision, 8, 33. 10.1167/8.7.33 19146265

[bibr11-20416695221109300] Curray J. R. (1956). The analysis of two-dimensional orientation data. The Journal of Geology, 64, 117–131.

[bibr12-20416695221109300] Fritsche M. Mostert P. de Lange F. P. (2017). Opposite effects of recent history on perception and decision. Current Biology: CB, 27, 590–595. 10.1016/j.cub.2017.01.006 28162897

[bibr13-20416695221109300] Fritsche M. Spaak E. de Lange F. P. (2020). A Bayesian and efficient observer model explains concurrent attractive and repulsive history biases in visual perception. eLife, 9, e55389. 10.7554/eLife.55389 32479264 PMC7286693

[bibr14-20416695221109300] Gepshtein S. Kubovy M. (2005). Stability and change in perception: Spatial organization in temporal context. Experimental Brain Research, 160, 487–495. 10.1007/s00221-004-2038-3 15517224

[bibr15-20416695221109300] Gronau Q. F. Sarafoglou A. Matzke D. Ly A. Boehm U. Marsman M. , … Steingroever H. (2017). A tutorial on bridge sampling. Journal of Mathematical Psychology, 81, 80–97. 10.1016/j.jmp.2017.09.005 29200501 PMC5699790

[bibr16-20416695221109300] Haaf J. M. Rouder J. N. (2019). Some do and some don’t? Accounting for variability of individual difference structures. Psychonomic Bulletin & Review, 26, 772–789. 10.3758/s13423-018-1522-x 30251148

[bibr17-20416695221109300] Kanai R. Rees G. (2011). The structural basis of inter-individual differences in human behaviour and cognition. Nature Reviews Neuroscience, 12, 231–242. 10.1038/nrn3000 21407245

[bibr18-20416695221109300] Kondo A. Murai Y. Whitney D. (2022). The test-retest reliability and spatial tuning of serial dependence in orientation perception. Journal of Vision, 22, 5. 10.1167/jov.22.4.5 PMC8944387 35293956

[bibr19-20416695221109300] Kubovy M. Holcombe A. O. Wagemans J. (1998). On the lawfulness of grouping by proximity. Cognitive Psychology, 35(1), 71–98. 10.1006/cogp.1997.0673 9520318

[bibr20-20416695221109300] Kubovy M. van den Berg M. (2002). Oblique effects in grouping: Surprising individual differences. Journal of Vision, 2, 480–480. 10.1167/2.7.48012678646

[bibr21-20416695221109300] Lieder I. Adam V. Frenkel O. Jaffe-Dax S. Sahani M. Ahissar M. (2019). Perceptual bias reveals slow-updating in autism and fast-forgetting in dyslexia. Nature Neuroscience, 22, 256–264. 10.1038/s41593-018-0308-9 30643299

[bibr22-20416695221109300] Manassi M. Liberman A. Kosovicheva A. Zhang K. Whitney D. (2018). Serial dependence in position occurs at the time of perception. Psychonomic Bulletin & Review, 25, 2245–2253. 10.3758/s13423-018-1454-5 29582377

[bibr23-20416695221109300] Mardia K. V. Jupp P. E. (2000). Directional Statistics. Wiley.

[bibr24-20416695221109300] Mattar M. G. Carter M. V. Zebrowitz M. S. Thompson-Schill S. L. Aguirre G. K. (2018). Individual differences in response precision correlate with adaptation bias. Journal of Vision, 18, 10.1167/18.13.18 PMC6314105 30593060

[bibr25-20416695221109300] Mattar M. G. Kahn D. A. Thompson-Schill S. L. Aguirre G. K. (2016). Varying timescales of stimulus integration unite neural adaptation and Prototype Formation. Current Biology, 26, 1669–1676. 10.1016/j.cub.2016.04.065 27321999 PMC4942354

[bibr26-20416695221109300] Maus G. W. Chaney W. Liberman A. Whitney D. (2013). The challenge of measuring long-term positive aftereffects. Current Biology : CB, 23, 10.1016/j.cub.2013.03.024 PMC3838704 23701683

[bibr27-20416695221109300] McGovern D. P. Walsh K. S. Bell J. Newell F. N. (2017). Individual differences in context-dependent effects reveal common mechanisms underlying the direction aftereffect and direction repulsion. Vision Research, 141, 109–116. 10.1016/j.visres.2016.08.009 27756699

[bibr28-20416695221109300] Miller J. Schwarz W. (2018). Implications of individual differences in on-average null effects. Journal of Experimental Psychology: General, 147, 377–397. 10.1037/xge0000367 29058941

[bibr29-20416695221109300] Mollon J. D. Bosten J. M. Peterzell D. H. Webster M. A. (2017). Individual differences in visual science: What can be learned and what is good experimental practice? Vision Research, 141, 4–15. 10.1016/j.visres.2017.11.001 29129731 PMC5730466

[bibr30-20416695221109300] Pascucci D. Mancuso G. Santandrea E. Libera C. D. Plomp G. Chelazzi L. (2019). Laws of concatenated perception: Vision goes for novelty, decisions for perseverance. PLOS Biology10.1371/journal.pbio.3000144 PMC6400421 30835720

[bibr31-20416695221109300] Peirce J. W. (2007). PsychoPy–Psychophysics software in Python. Journal of Neuroscience Methods, 162, 8–13. 10.1016/j.jneumeth.2006.11.017 17254636 PMC2018741

[bibr32-20416695221109300] R Core Team. (2021). *R: A language and environment for statistical computing*. Vienna, Austria: R Foundation for Statistical Computing. Retrieved from https://www.R-project.org/

[bibr33-20416695221109300] Rouder J. N. (2014). Optional stopping: No problem for Bayesians. Psychonomic Bulletin & Review, 21, 301–308. 10.3758/s13423-014-0595-4 24659049

[bibr34-20416695221109300] Rouder J. N. (2019). Optional stopping and the interpretation of the Bayes factor. PsyArXiv10.31234/osf.io/m6dhw

[bibr35-20416695221109300] Rouder J. N. Haaf J. M. (2019). A psychometrics of individual differences in experimental tasks. Psychonomic Bulletin & Review, 26, 452–467. 10.3758/s13423-018-1558-y 30911907

[bibr36-20416695221109300] Sadil P. Cowell R. Huber D. E. (2021). The yin-yang of serial dependence effects: Every response is both an attraction to the prior response and a repulsion from the prior stimulus. PsyArXiv10.31234/osf.io/f52yz PMC11488665 37566217

[bibr37-20416695221109300] Schönbrodt F. D. Wagenmakers E.-J. (2018). Bayes factor design analysis: Planning for compelling evidence. Psychonomic Bulletin & Review, 25, 128–142. 10.3758/s13423-017-1230-y 28251595

[bibr38-20416695221109300] Schönbrodt F. D. Wagenmakers E. -J. Zehetleitner M. Perugini M. (2017). Sequential hypothesis testing with Bayes factors: Efficiently testing mean differences. Psychological Methods, 22, 322–339. 10.1037/met0000061 26651986

[bibr39-20416695221109300] Schwiedrzik C. M. Ruff C. C. Lazar A. Leitner F. C. Singer W. Melloni L. (2014). Untangling perceptual memory: Hysteresis and adaptation map into separate cortical networks. Cerebral Cortex, 24, 1152–1164. 10.1093/cercor/bhs396 23236204 PMC3977616

[bibr40-20416695221109300] Schwiedrzik C. M. Sudmann S. S. Thesen T. Wang X. Groppe D. M. Mégevand P. , … Melloni L. (2018). Medial prefrontal cortex supports perceptual memory. Current Biology, 28, R1094–R1095. 10.1016/j.cub.2018.07.066 30253147

[bibr41-20416695221109300] Snyder H. K. Rafferty S. M. Haaf J. M. Rouder J. N. (2019). Common or distinct attention mechanisms for contrast and assimilation? Attention, Perception, & Psychophysics, 81, 1944–1950. 10.3758/s13414-019-01713-8 31044394

[bibr42-20416695221109300] Snyder J. S. Schwiedrzik C. M. Vitela A. D. Melloni L. (2015). How previous experience shapes perception in different sensory modalities. Frontiers in Human Neuroscience, 9. 10.3389/fnhum.2015.00594 PMC4628108 26582982

[bibr43-20416695221109300] Song C. Schwarzkopf D. S. Lutti A. Li B. Kanai R. Rees G. (2013a). Effective connectivity within human primary visual cortex predicts interindividual diversity in illusory perception. Journal of Neuroscience, 33, 18781–18791. 10.1523/JNEUROSCI.4201-12.2013 24285885 PMC3841448

[bibr44-20416695221109300] Song C. Schwarzkopf D. S. Rees G. (2013b). Variability in visual cortex size reflects tradeoff between local orientation sensitivity and global orientation modulation. Nature Communications, 4, 2201. 10.1038/ncomms3201 PMC3731653 23887643

[bibr45-20416695221109300] Stein H. Barbosa J. Rosa-Justicia M. Prades L. Morató A. Galan-Gadea A. , … Compte A. (2020). Reduced serial dependence suggests deficits in synaptic potentiation in anti-NMDAR encephalitis and schizophrenia. Nature Communications, 11, 4250. 10.1038/s41467-020-18033-3 PMC7447775 32843635

[bibr46-20416695221109300] Urai A. E. Braun A. Donner T. H. (2017). Pupil-linked arousal is driven by decision uncertainty and alters serial choice bias. Nature Communications, 8, 14637. 10.1038/ncomms14637 PMC5337963 28256514

[bibr47-20416695221109300] Van der Hulst E. van Heusden E. Wagemans J. Moors P. (2022). *Grouping by proximity and luminance similarity is additive for everyone: An analysis of individual differences in grouping sensitivity*. Retrieved from osf.io/p845j. 10.3758/s13414-023-02770-w 37740153

[bibr48-20416695221109300] Van Rossum G. Drake, Jr. F. L. (1995). *Python reference manual*. Centrum voor Wiskunde en Informatica Amsterdam.

[bibr49-20416695221109300] Wagemans J. (2018). Perceptual organization. In J. T. Wixted (Series Ed.) & J. Serences (Vol. Ed.) (Ed.), *The Stevens’ Handbook of Experimental Psychology and Cognitive Neuroscience: Vol. 2. Sensation, Perception & Attention* (pp. 803–872). Hoboken, NJ: John Wiley & Sons, Inc. 10.1002/9781119170174.epcn218.

[bibr50-20416695221109300] Wagemans J. Claessens P. M. E. Moors P. (2018). *Perceptual grouping in dot lattices revisited*. 41st European Conference on Visual Perception (ECVP), Trieste, Italy: Abstract published in *Perception*, 48(S1) (Supplement). 10.1177/0301006618824879.

[bibr51-20416695221109300] Wagenmakers E.-J. Lodewyckx T. Kuriyal H. Grasman R. (2010). Bayesian hypothesis testing for psychologists: A tutorial on the SavageDickey method. Cognitive Psychology, 60(3), 158–189. 10.1016/j.cogpsych.2009.12.001 20064637

[bibr52-20416695221109300] Wexler M. Duyck M. Mamassian P. (2015). Persistent states in vision break universality and time invariance. Proceedings of the National Academy of Sciences of the U.S.A., 112, 14990–14995. 10.1073/pnas.1508847112 PMC4672830 26627250

[bibr53-20416695221109300] Zhang H. Alais D. (2019). Individual difference in serial dependence results from opposite influences of perceptual choices and motor responses. bioRxiv 631309. 10.1101/631309 PMC7438638 32744618

