# Supplementary material for: Same stimulus, same temporal context, different percept? Individual differences in hysteresis and adaptation when perceiving multistable dot lattices
Source: Iperception. 2022 Jul 6;13(4):20416695221109300. doi: 10.1177/20416695221109300 (PMC9274434; doi:10.1177/20416695221109300)
Supplement: sj-pdf-1-ipe-10.1177_20416695221109300 - Supplemental material for Same stimulus, same temporal context, different percept? Individual differences in hysteresis and adaptation when perceiving multistable dot lattices [file sj-pdf-1-ipe-10.1177_20416695221109300.pdf]

Appendix

Supplementary Figures and Information

As indicated in the Methods section, for all our analyses we used R [Version 4.0.4; R Core Team (2021)] and the R-packages *BayesFactor* [Version 0.9.12.4.2; Morey & Rouder (2018)], *brms* [Version 2.16.3; Bürkner (2017); Bürkner (2018); Bürkner (2021)], *coda* [Version 0.19.4; Plummer, Best, Cowles, & Vines (2006)], *cowplot* [Version 1.1.1; Wilke (2020)], *devtools* [Version 2.4.3; Wickham, Hester, Chang, & Bryan (2021)], *dplyr* [Version 1.0.7; Wickham, François, Henry, & Müller (2021)], *ellipse* [Version 0.4.2; Murdoch & Chow (2020)], *forcats* [Version 0.5.1; Wickham (2021a)], *ggforce* [Version 0.3.3; Pedersen (2021)], *ggplot2* [Version 3.3.5; Wickham (2016)], *ggstance* [Version 0.3.5; Henry, Wickham, & Chang (2020)], *glue* [Version 1.5.1; Hester & Bryan (2021)], *here* [Version 1.0.1; Müller (2020)], *kableExtra* [Version 1.3.4; Zhu (2021)], *knitr* [Version 1.36; Xie (2015)], *lubridate* [Version 1.8.0; Grolemund & Wickham (2011)], *Matrix* [Version 1.3.2; Bates & Maechler (2021)], *papaja* [Version 0.1.0.9997; Aust & Barth (2020)], *patchwork* [Version 1.1.1; Pedersen (2020)], *purrr* [Version 0.3.4; Henry & Wickham (2020)], *Rcpp* [Version 1.0.7; Eddelbuettel & François (2011); Eddelbuettel & Balamuta (2018)], *readr* [Version 2.1.1; Wickham, Hester, & Bryan (2021)], *rstan* [Version 2.21.3; Stan Development Team (2021)], *StanHeaders* [Version 2.21.0.7; Stan Development Team (2020)], *stringr* [Version 1.4.0; Wickham (2019)], *tibble* [Version 3.1.6; Müller & Wickham (2021)], *tidybayes* [Version 3.0.1; Kay (2021)], *tidyr* [Version 1.1.4; Wickham (2021b)], *tidyverse* [Version 1.3.1; Wickham et al. (2019)], and *usethis* [Version 2.1.3; Wickham, Bryan, & Barrett (2021)].

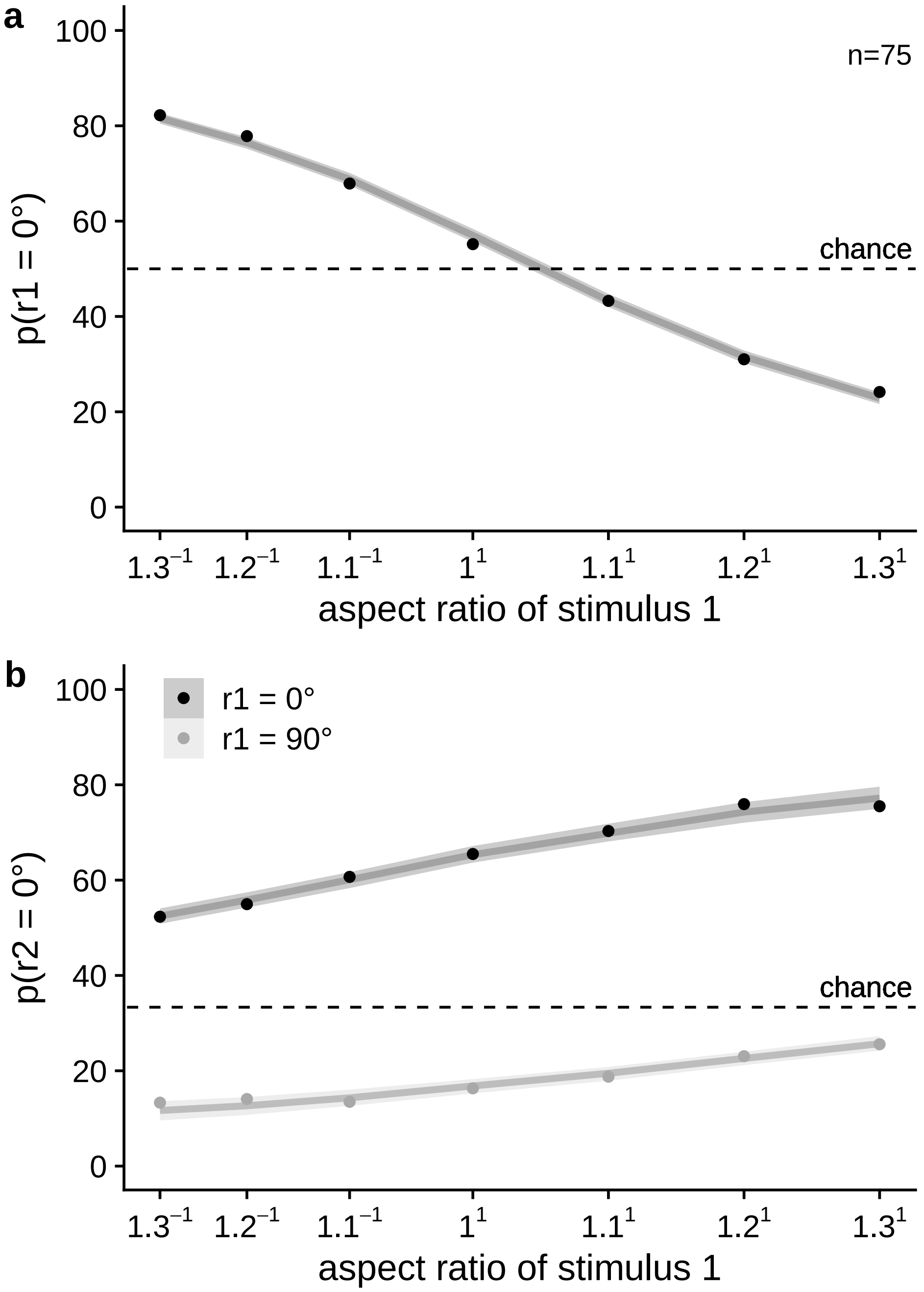

Figure A1. (a) Mean response to the first stimulus dependent on aspect ratio (percentage). The probability of responding 0° to the first stimulus decreases with aspect ratio (|a|/|b|). The value of aspect ratio increases with increasing distance in the 0°-orientation, leading to more 90° responses. (b) Mean response to the second stimulus dependent on aspect ratio (percentage). The probability of responding 0° to the second stimulus increases with aspect ratio (|a|/|b|; i.e., adaptation effect), and increases when the first stimulus was perceived as 0° rather than 90° (i.e., hysteresis effect). Dots indicate observed values. In addition, mean posterior predictions and their 95% highest density continuous intervals are shown.

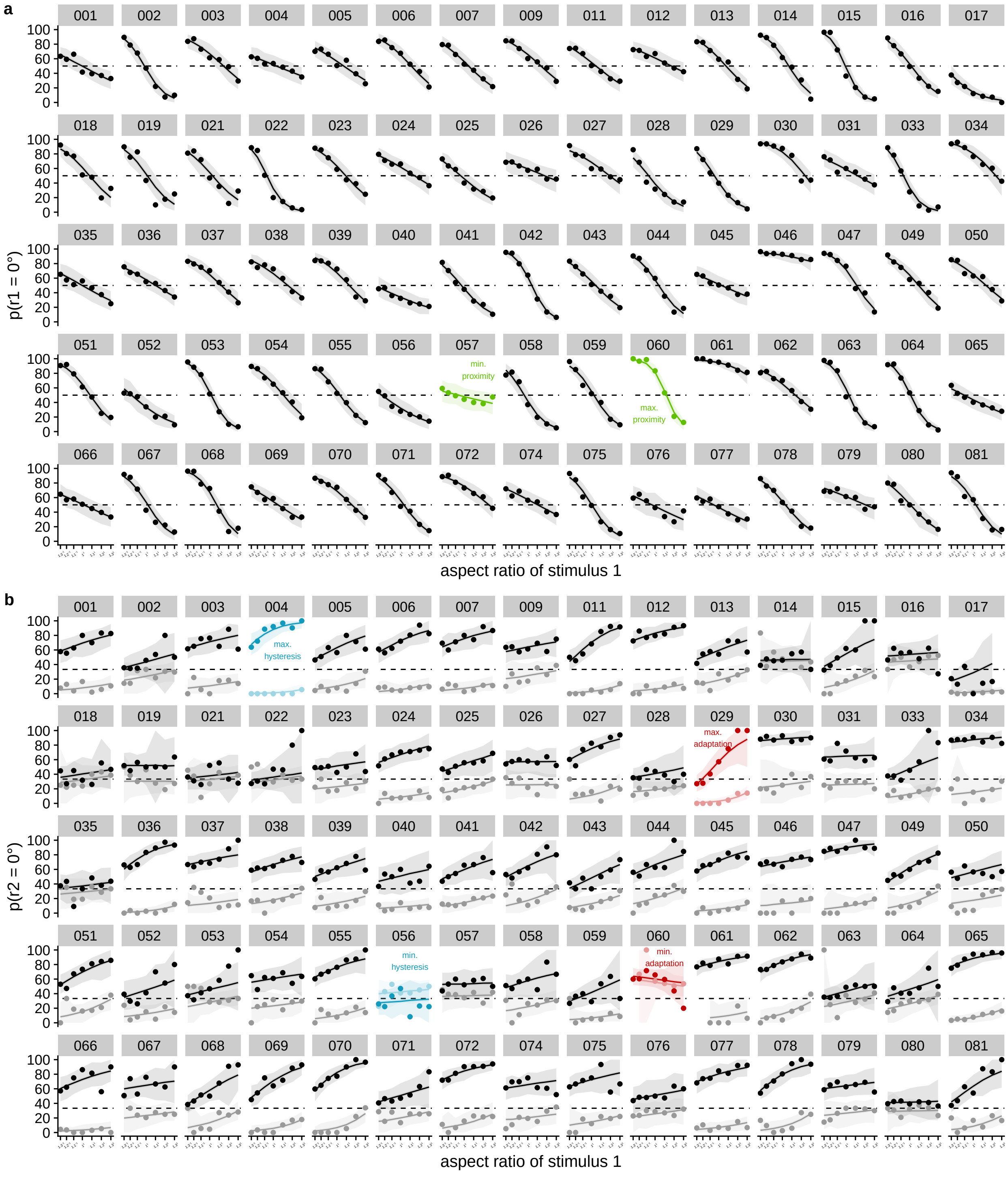

Figure A2. (a) Mean individual responses to the first stimulus dependent on aspect ratio (percentage). The probability of responding 0° to the first stimulus decreases with aspect ratio (|a|/|b|). The value of aspect ratio increases with increasing distance in the 0°-orientation, leading to more 90° responses. Dots indicate observed values. In addition, mean posterior predictions and their 95% highest density continuous intervals are shown. Plots for participants with the smallest and largest estimated proximity effect are indicated in red. (b) Mean individual responses to the second stimulus dependent on aspect ratio (percentage). The probability of responding 0° to the second stimulus increases with aspect ratio (|a|/|b|; i.e., adaptation effect), and increases when the first stimulus was perceived as 0° rather than 90° (i.e., hysteresis effect). Dots indicate observed values. In addition, mean posterior predictions and their 95% highest density continuous intervals are shown. Plots for participants with the smallest and largest estimated hysteresis effect are indicated in blue, participants with the smallest and largest estimated adaptation effect are indicated in red.

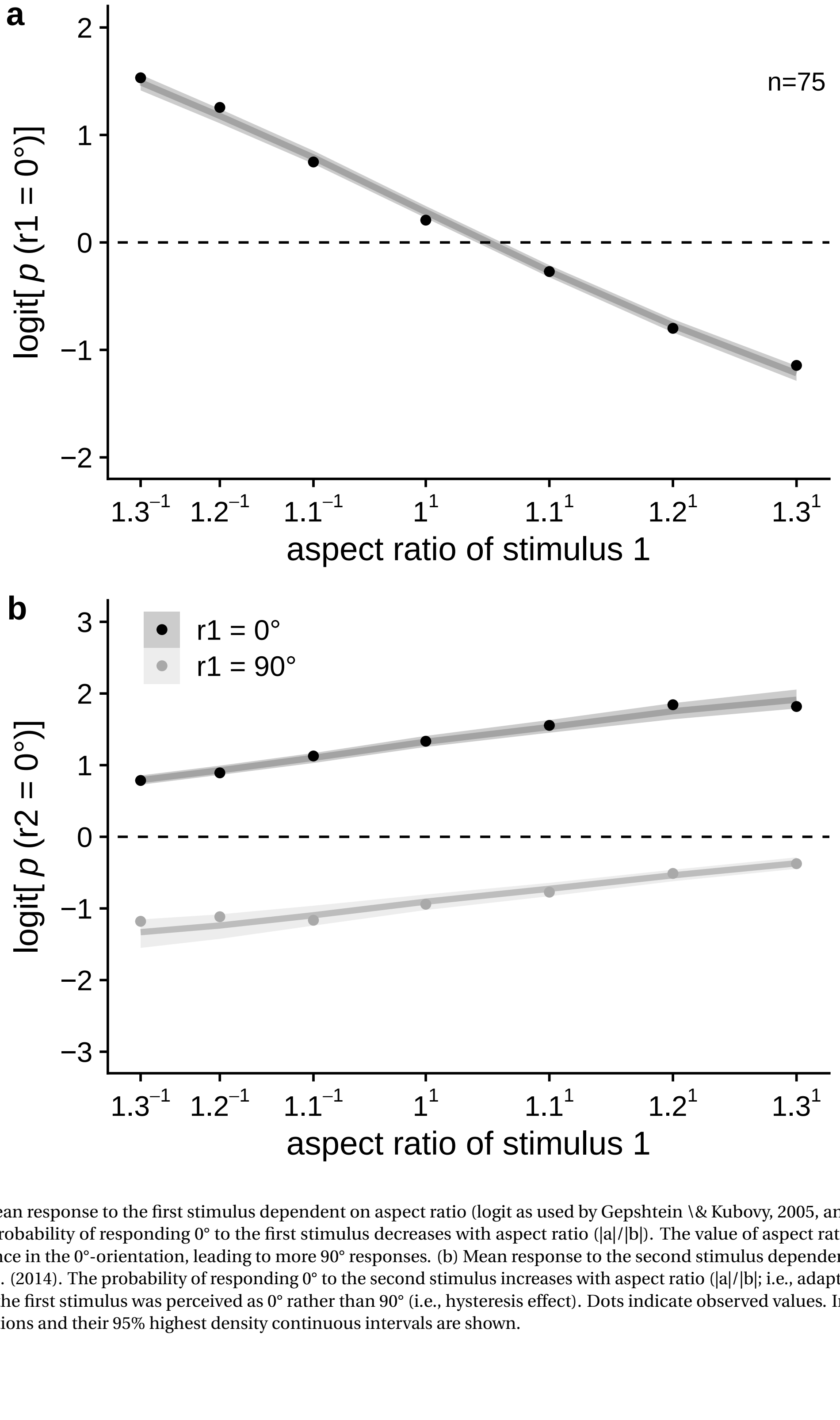

Figure A3. (a) Mean response to the first stimulus dependent on aspect ratio (logit as used by Gepshtein \& Kubovy, 2005, and Schwiedrzik et al., 2014). The probability of responding 0° to the first stimulus decreases with aspect ratio (|a|/|b|). The value of aspect ratio increases with increasing distance in the 0°-orientation, leading to more 90° responses. (b) Mean response to the second stimulus dependent on aspect ratio Schwiedrzik et al. (2014). The probability of responding 0° to the second stimulus increases with aspect ratio (|a|/|b|; i.e., adaptation effect), and increases when the first stimulus was perceived as 0° rather than 90° (i.e., hysteresis effect). Dots indicate observed values. In addition, mean posterior predictions and their 95% highest density continuous intervals are shown.

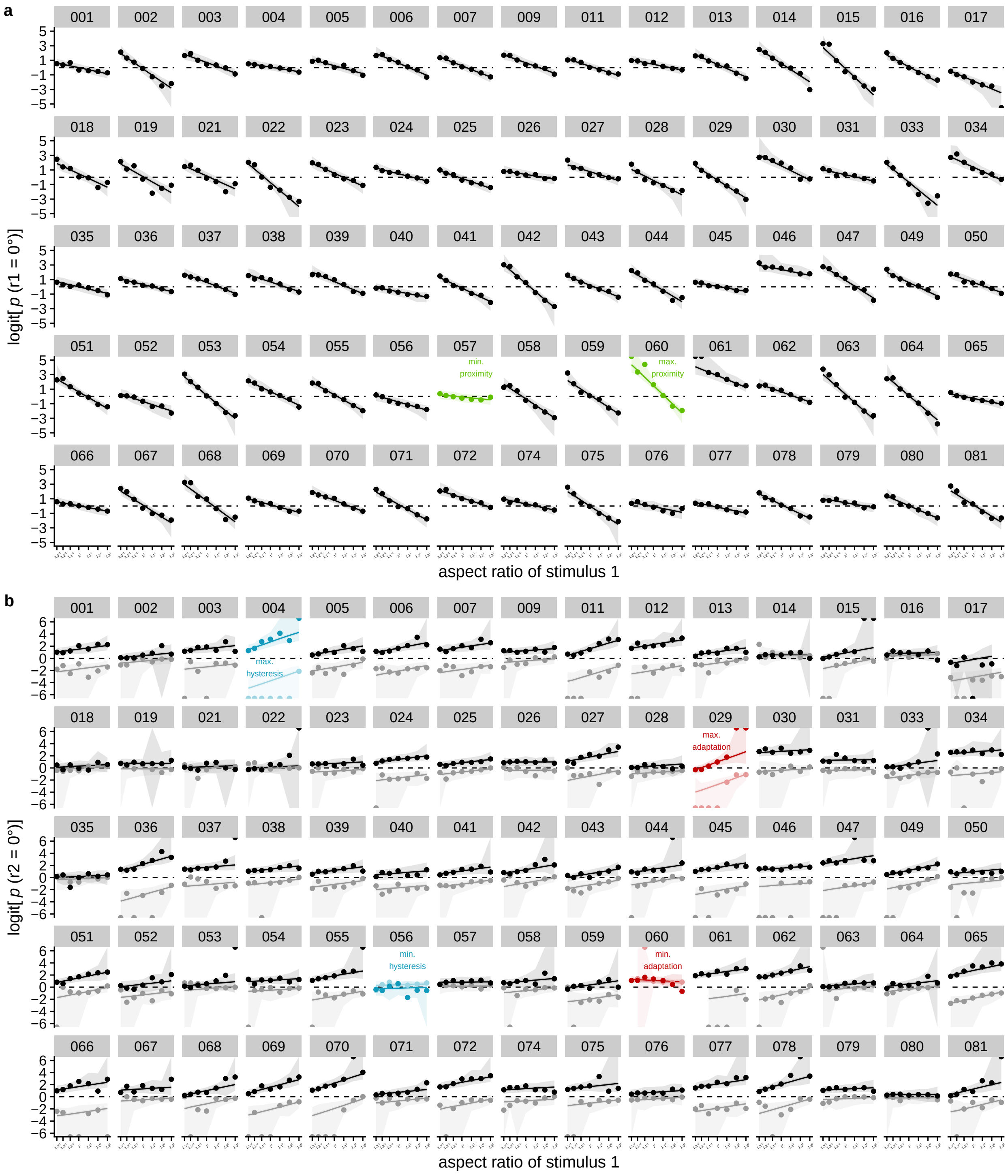

Figure A4. (a) Mean individual responses to the first stimulus dependent on aspect ratio (logit as used by Gepshtein \& Kubovy, 2005, and Schwiedrzik et al., 2014). The probability of responding 0° to the first stimulus decreases with aspect ratio (|a|/|b|). The value of aspect ratio increases with increasing distance in the 0°-orientation, leading to more 90° responses. Dots indicate observed values. In addition, mean posterior predictions and their 95% highest density continuous intervals are shown. Plots for participants with the smallest and largest estimated proximity effect are indicated in red. (b) Mean individual responses to the second stimulus dependent on aspect ratio Schwiedrzik et al. (2014). The probability of responding 0° to the second stimulus increases with aspect ratio (|a|/|b|; i.e., adaptation effect), and increases when the first stimulus was perceived as 0° rather than 90° (i.e., hysteresis effect). Dots indicate observed values. In addition, mean posterior predictions and their 95% highest density continuous intervals are shown. Plots for participants with the smallest and largest estimated hysteresis effect are indicated in blue, participants with the smallest and largest estimated adaptation effect are indicated in red.

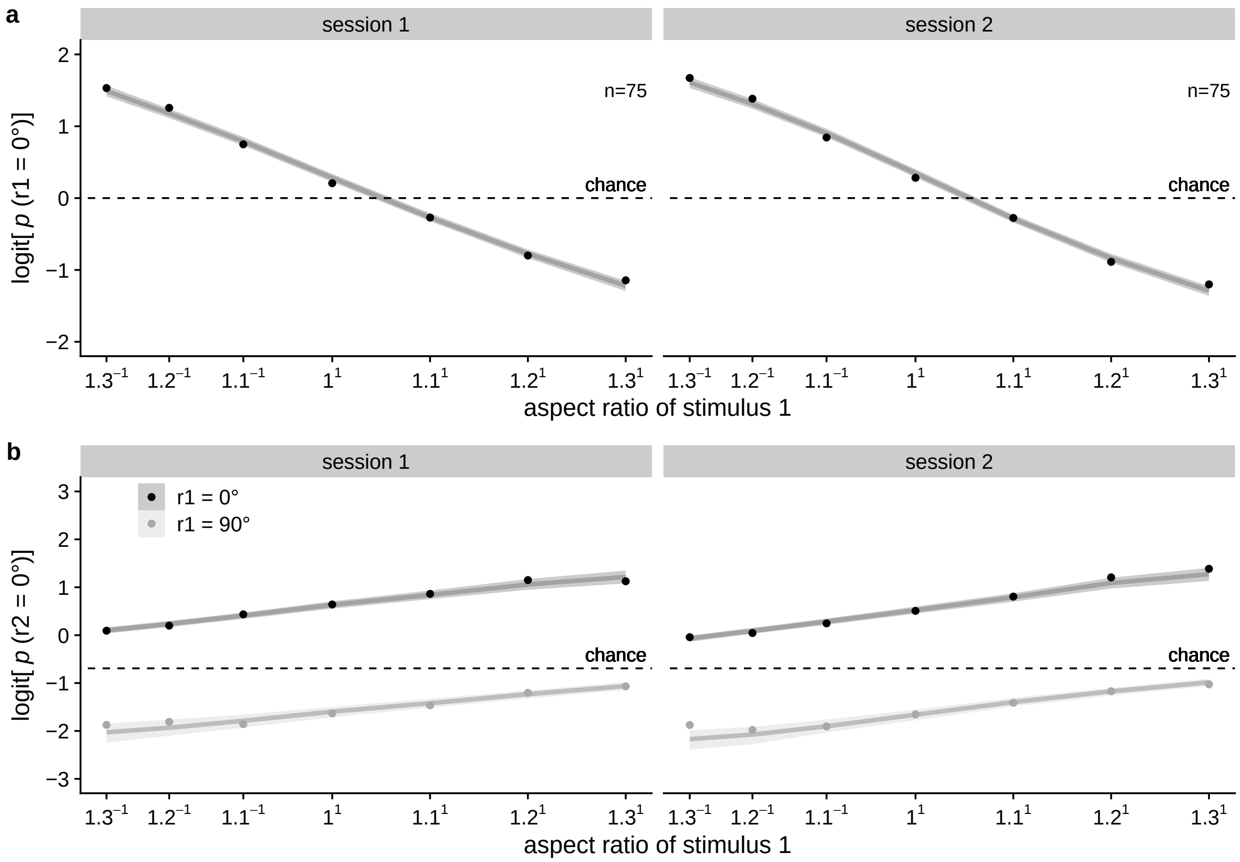

Figure A5. (a) Mean response to the first stimulus dependent on aspect ratio (logit), for both the first and the second session. The probability of responding 0° to the first stimulus decreases with aspect ratio (|a|/|b|). The value of aspect ratio increases with increasing distance in the 0°-orientation, leading to more 90° responses. (b) Mean response to the second stimulus dependent on aspect ratio (logit), for both the first and the second session. The probability of responding 0° to the second stimulus increases with aspect ratio (|a|/|b|; i.e., adaptation effect), and increases when the first stimulus was perceived as 0° rather than 90° (i.e., hysteresis effect). Dots indicate observed values. In addition, mean posterior predictions and their 95% highest density continuous intervals are shown.

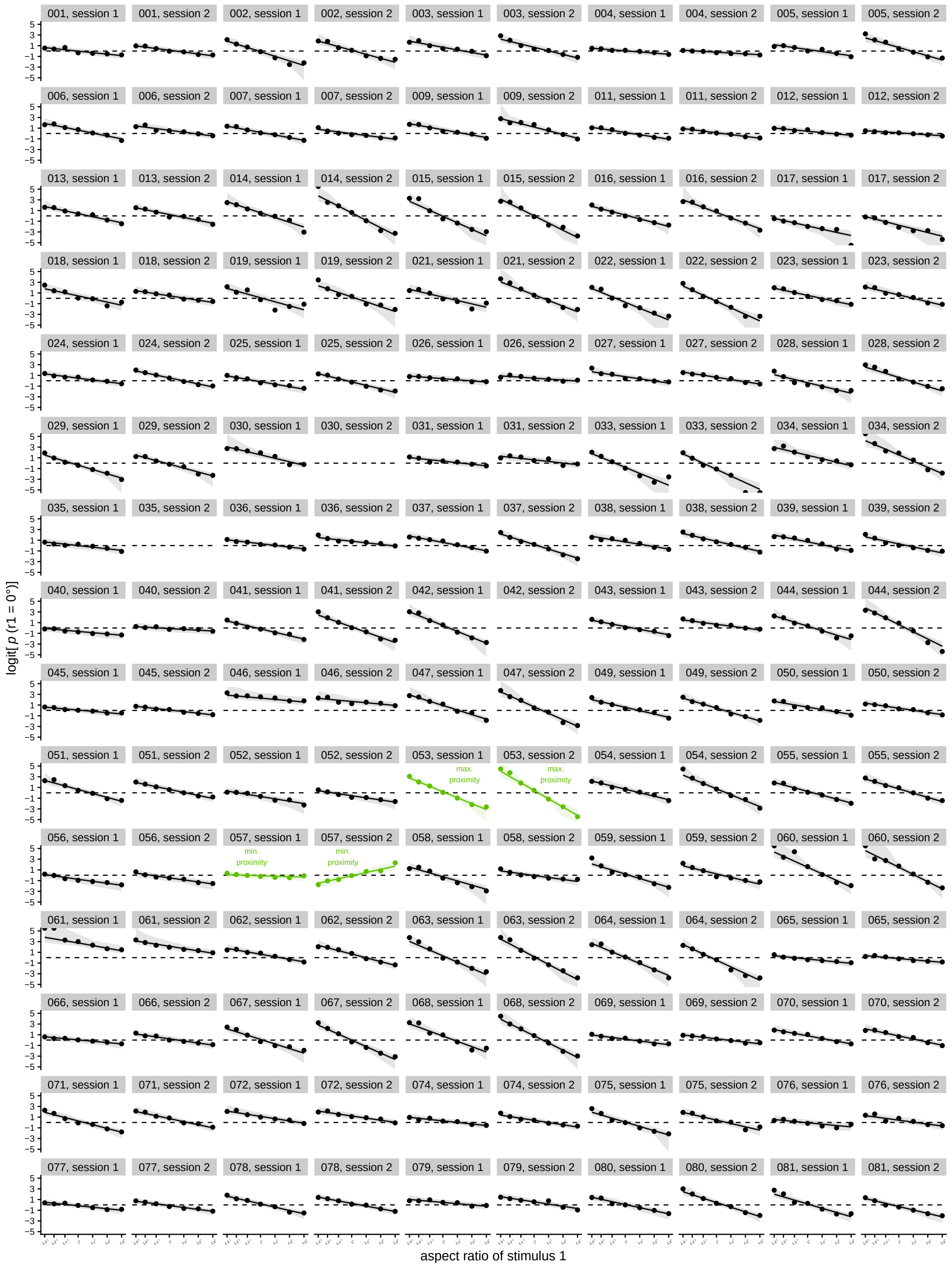

Figure A6. Mean individual responses to the first stimulus dependent on aspect ratio (logit), for both the first and the second session. The probability of responding 0° to the first stimulus decreases with aspect ratio (|a|/|b|). The value of aspect ratio increases with increasing distance in the 0°-orientation, leading to more 90° responses. Dots indicate observed values. In addition, mean posterior predictions and their 95% highest density continuous intervals are shown. Plots for participants with the smallest and largest estimated proximity effect are indicated in green.

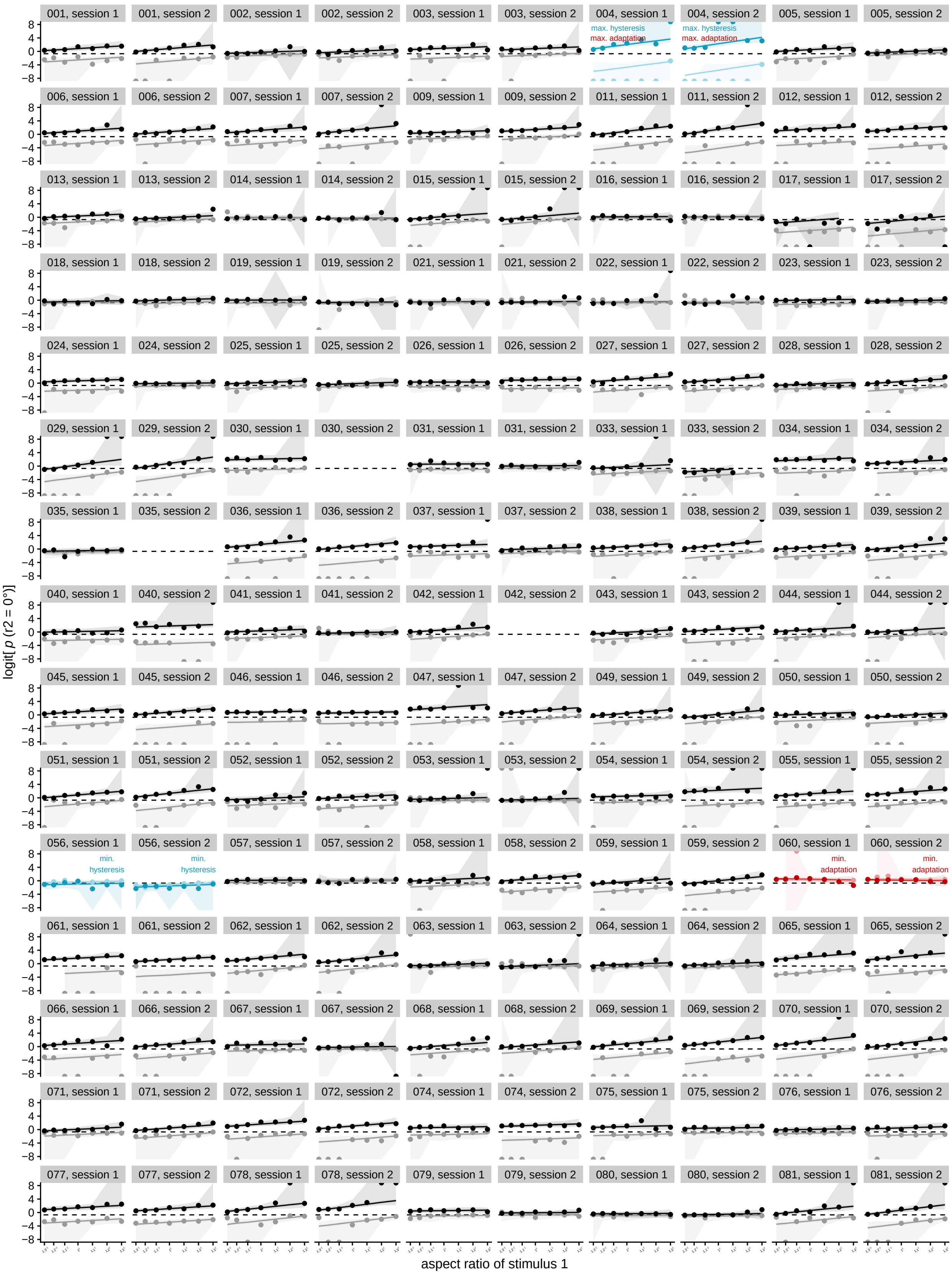

Figure A7. Mean individual responses to the second stimulus dependent on aspect ratio (logit), for both the first and the second session. The probability of responding 0° to the second stimulus increases with aspect ratio (|a|/|b|; i.e., adaptation effect), and increases when the first stimulus was perceived as 0° rather than 90° (i.e., hysteresis effect). Dots indicate observed values. In addition, mean posterior predictions and their 95% highest density continuous intervals are shown. Plots for participants with the smallest and largest estimated hysteresis effect are indicated in blue, participants with the smallest and largest estimated adaptation effect are indicated in red.

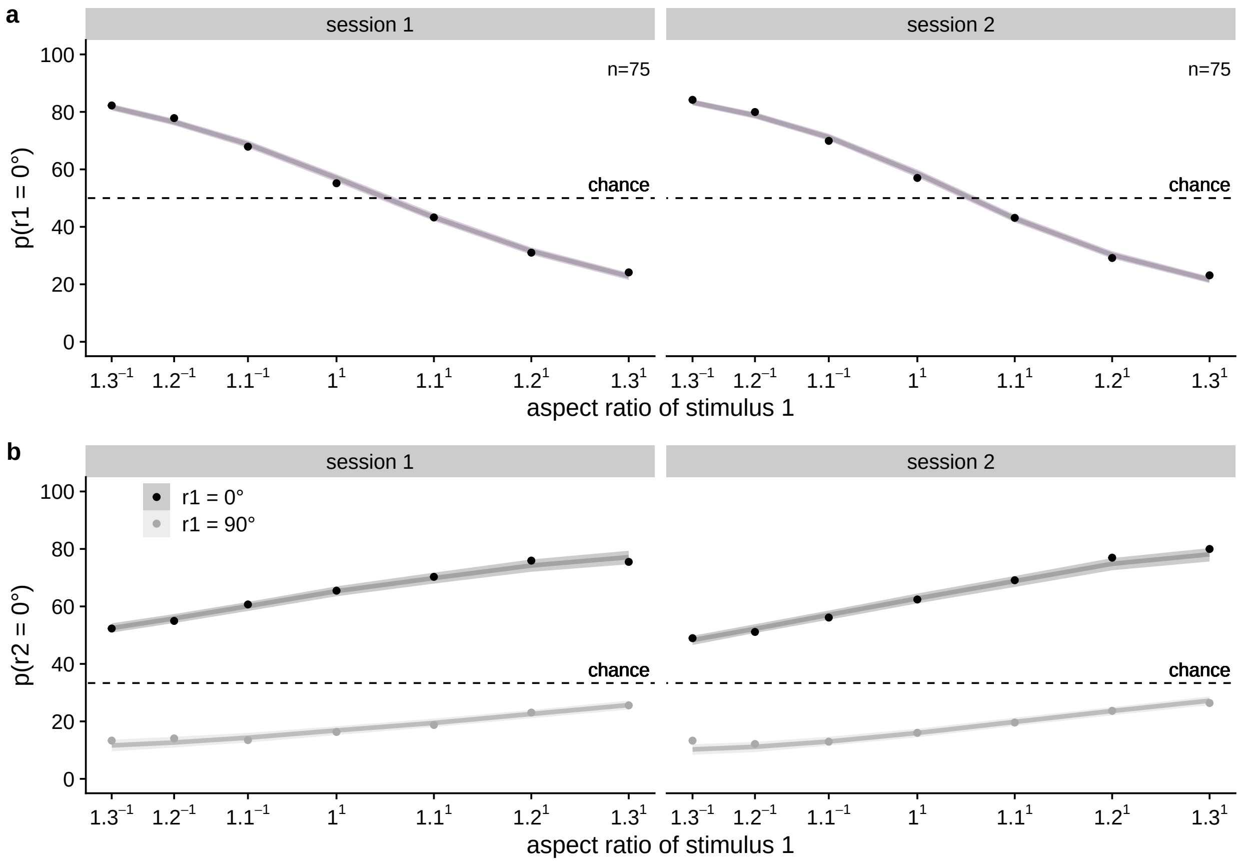

Figure A8. (a) Mean response to the first stimulus dependent on aspect ratio (percentage), for both the first and the second session. The probability of responding 0° to the first stimulus decreases with aspect ratio (|a|/|b|). The value of aspect ratio increases with increasing distance in the 0°-orientation, leading to more 90° responses. (b) Mean response to the second stimulus dependent on aspect ratio (probability), for both the first and the second session. The probability of responding 0° to the second stimulus increases with aspect ratio (|a|/|b|; i.e., adaptation effect), and increases when the first stimulus was perceived as 0° rather than 90° (i.e., hysteresis effect). Dots indicate observed values. In addition, mean posterior predictions and their 95% highest density continuous intervals are shown.

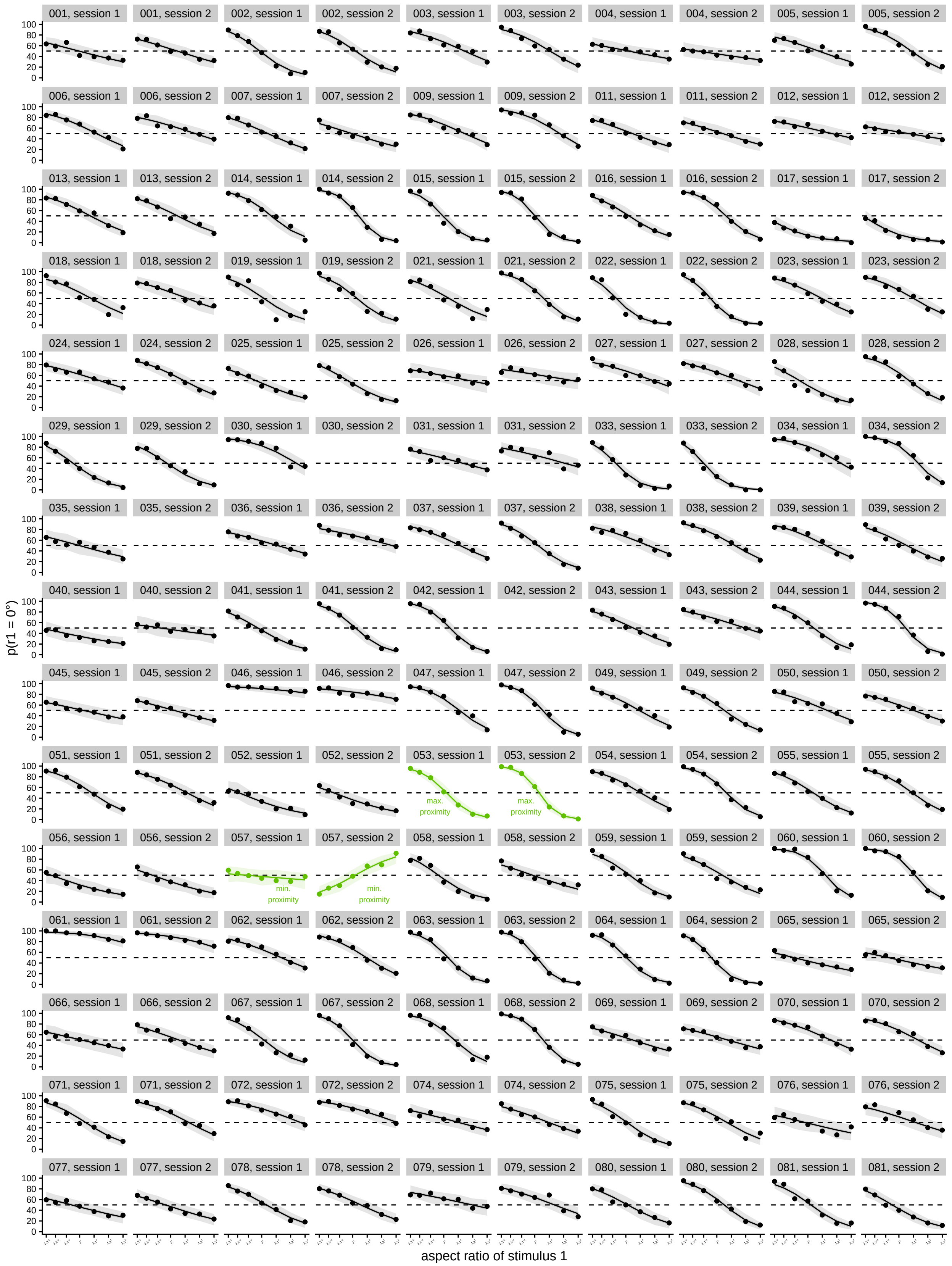

Figure A9. Mean individual responses to the first stimulus dependent on aspect ratio (percentage), for both the first and the second session. The probability of responding 0° to the first stimulus decreases with aspect ratio (|a|/|b|). The value of aspect ratio increases with increasing distance in the 0°-orientation, leading to more 90° responses. Dots indicate observed values. In addition, mean posterior predictions and their 95% highest density continuous intervals are shown. Plots for participants with the smallest and largest estimated proximity effect are indicated in green.

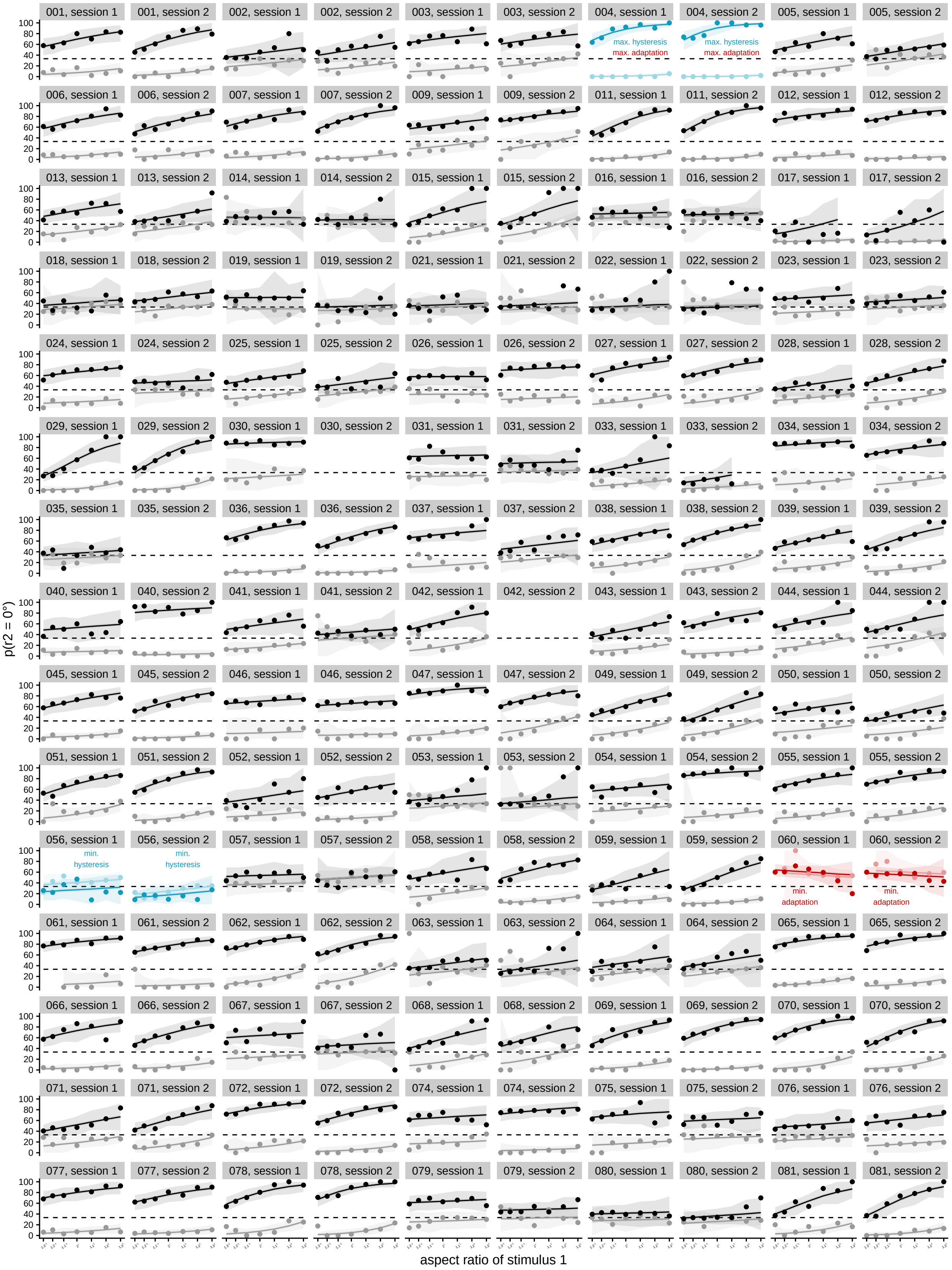

Figure A10. Mean individual responses to the second stimulus dependent on aspect ratio (percentage), for both the first and the second session. The probability of responding 0° to the second stimulus increases with aspect ratio (|a|/|b|; i.e., adaptation effect), and increases when the first stimulus was perceived as 0° rather than 90° (i.e., hysteresis effect). Dots indicate observed values. In addition, mean posterior predictions and their 95% highest density continuous intervals are shown. Plots for participants with the smallest and largest estimated hysteresis effect are indicated in blue, participants with the smallest and largest estimated adaptation effect are indicated in red.

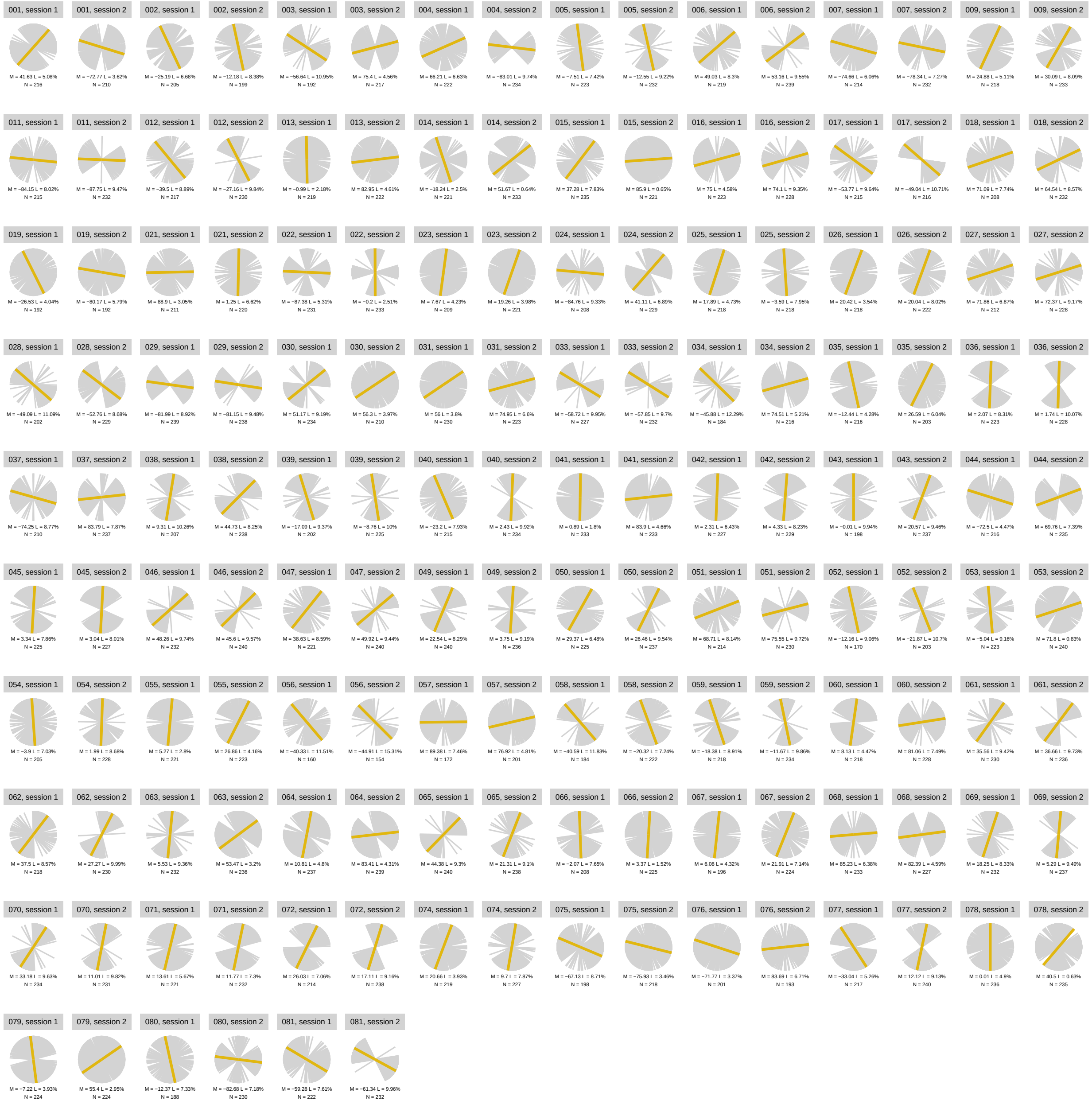

Figure A11. Visual representation of the absolute orientation bias of an individual, corrected for implausible 90° responses, separately for the first and the second session. The colored oriented line indicates the mean direction of the absolute orientation bias per individual. Also the numeric values for the mean direction (M) and the magnitude (L) of the absolute orientation bias and the number of included trials (N) per individual are given.

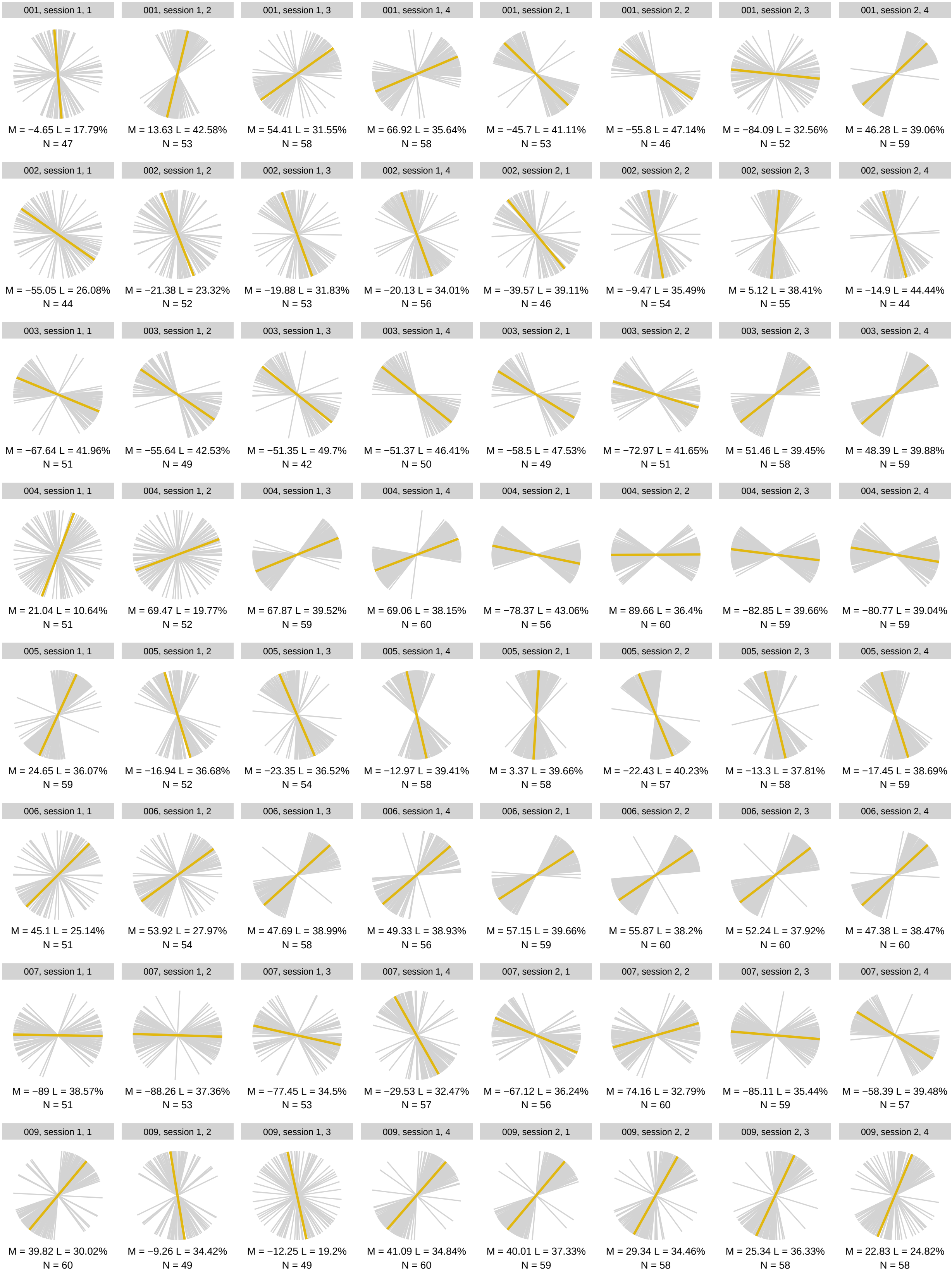

Figure A12. Visual representation of the absolute orientation bias of an individual per block of 60 trials, corrected for implausible 90° responses, separately for the first and the second session (Part 1/10). The colored oriented line indicates the mean direction of the absolute orientation bias per individual per block. Also the numeric values for the mean direction (M) and the magnitude (L) of the absolute orientation bias and the number of included trials (N) per individual per block are given.

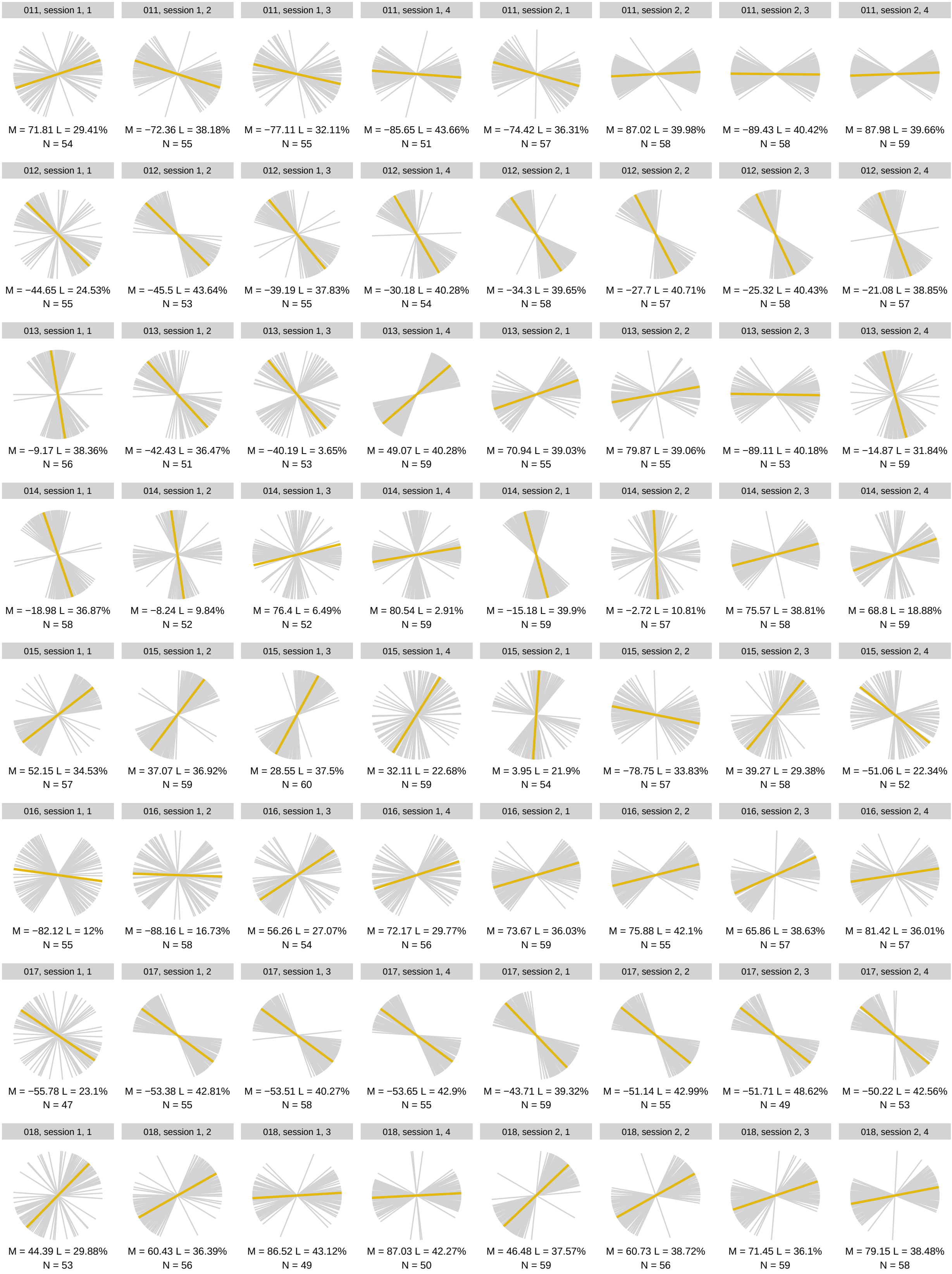

Figure A13. Visual representation of the absolute orientation bias of an individual per block of 60 trials, corrected for implausible 90° responses, separately for the first and the second session (Part 2/10). The colored oriented line indicates the mean direction of the absolute orientation bias per individual per block. Also the numeric values for the mean direction (M) and the magnitude (L) of the absolute orientation bias and the number of included trials (N) per individual per block are given.

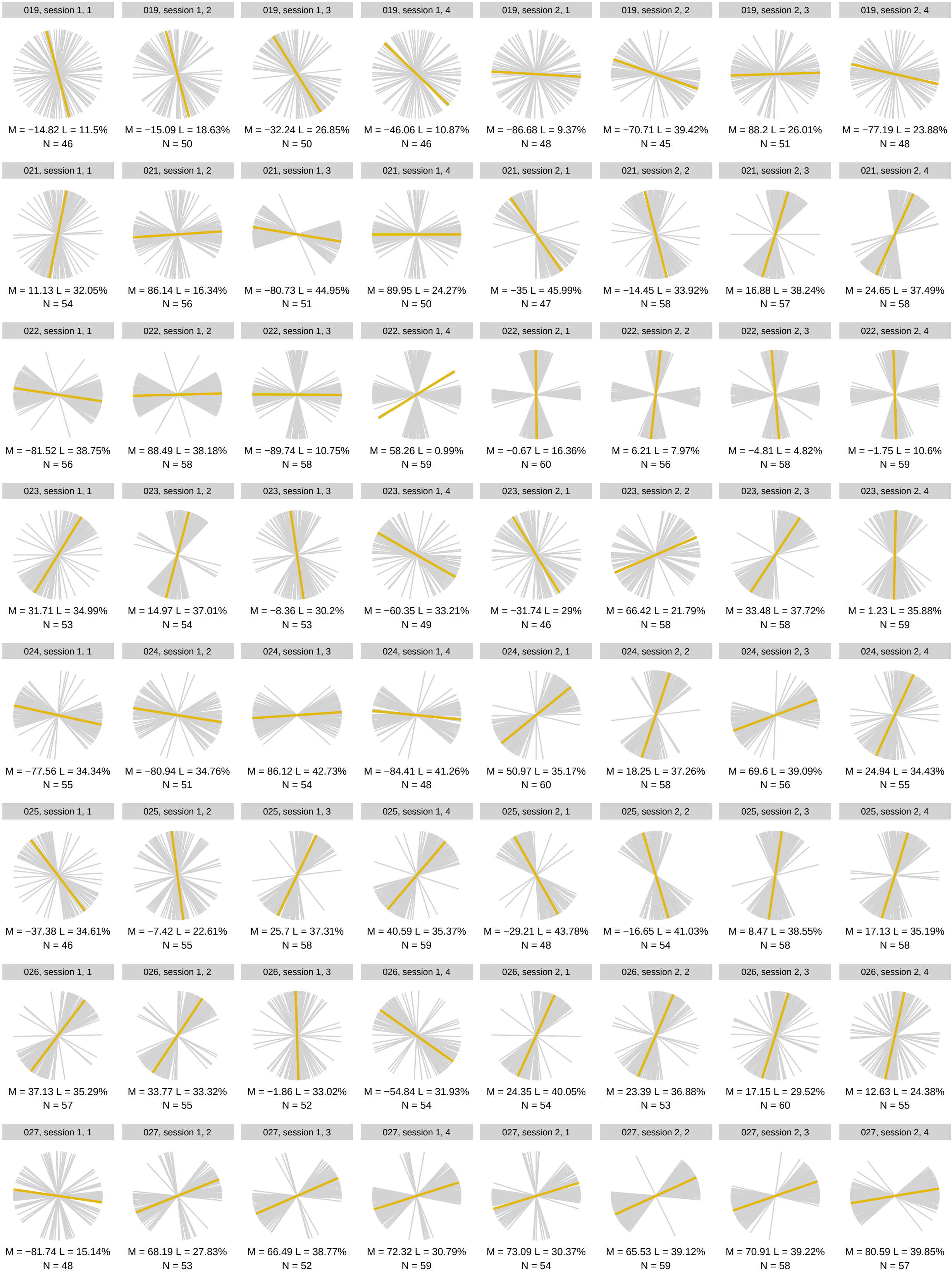

Figure A14. Visual representation of the absolute orientation bias of an individual per block of 60 trials, corrected for implausible 90° responses, separately for the first and the second session (Part 3/10). The colored oriented line indicates the mean direction of the absolute orientation bias per individual per block. Also the numeric values for the mean direction (M) and the magnitude (L) of the absolute orientation bias and the number of included trials (N) per individual per block are given.

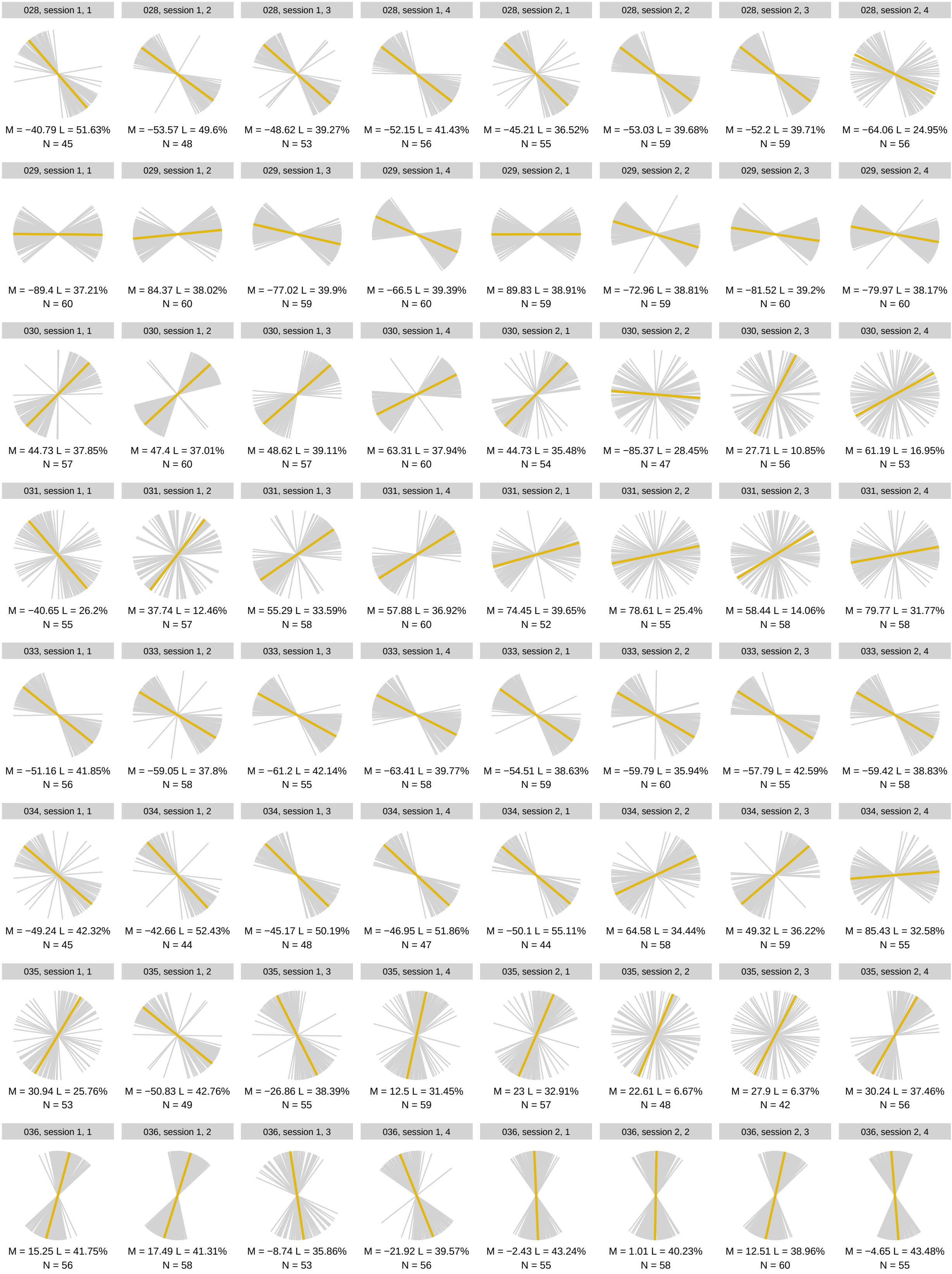

Figure A15. Visual representation of the absolute orientation bias of an individual per block of 60 trials, corrected for implausible 90° responses, separately for the first and the second session (Part 4/10). The colored oriented line indicates the mean direction of the absolute orientation bias per individual per block. Also the numeric values for the mean direction (M) and the magnitude (L) of the absolute orientation bias and the number of included trials (N) per individual per block are given.

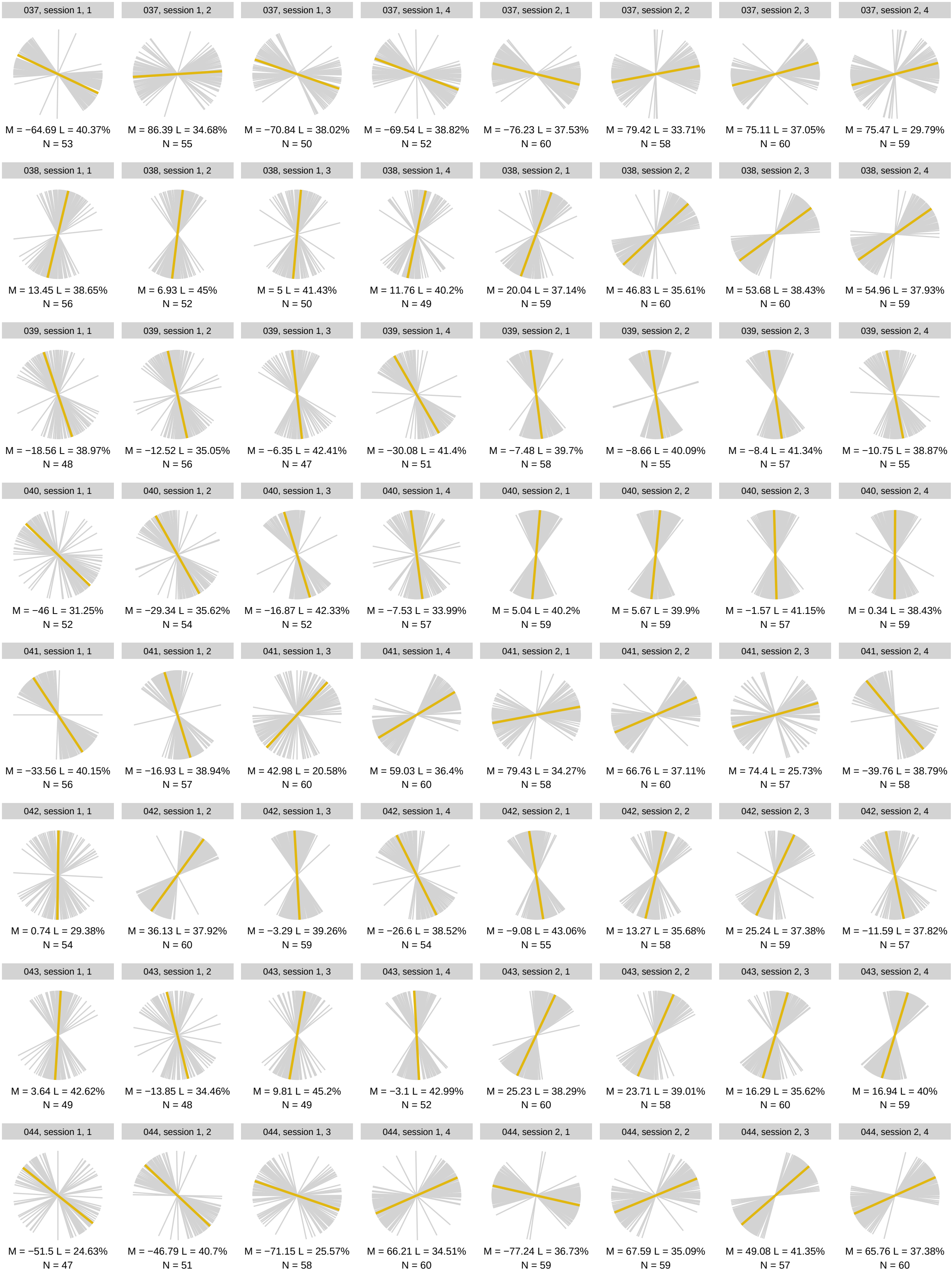

Figure A16. Visual representation of the absolute orientation bias of an individual per block of 60 trials, corrected for implausible 90° responses, separately for the first and the second session (Part 5/10). The colored oriented line indicates the mean direction of the absolute orientation bias per individual per block. Also the numeric values for the mean direction (M) and the magnitude (L) of the absolute orientation bias and the number of included trials (N) per individual per block are given.

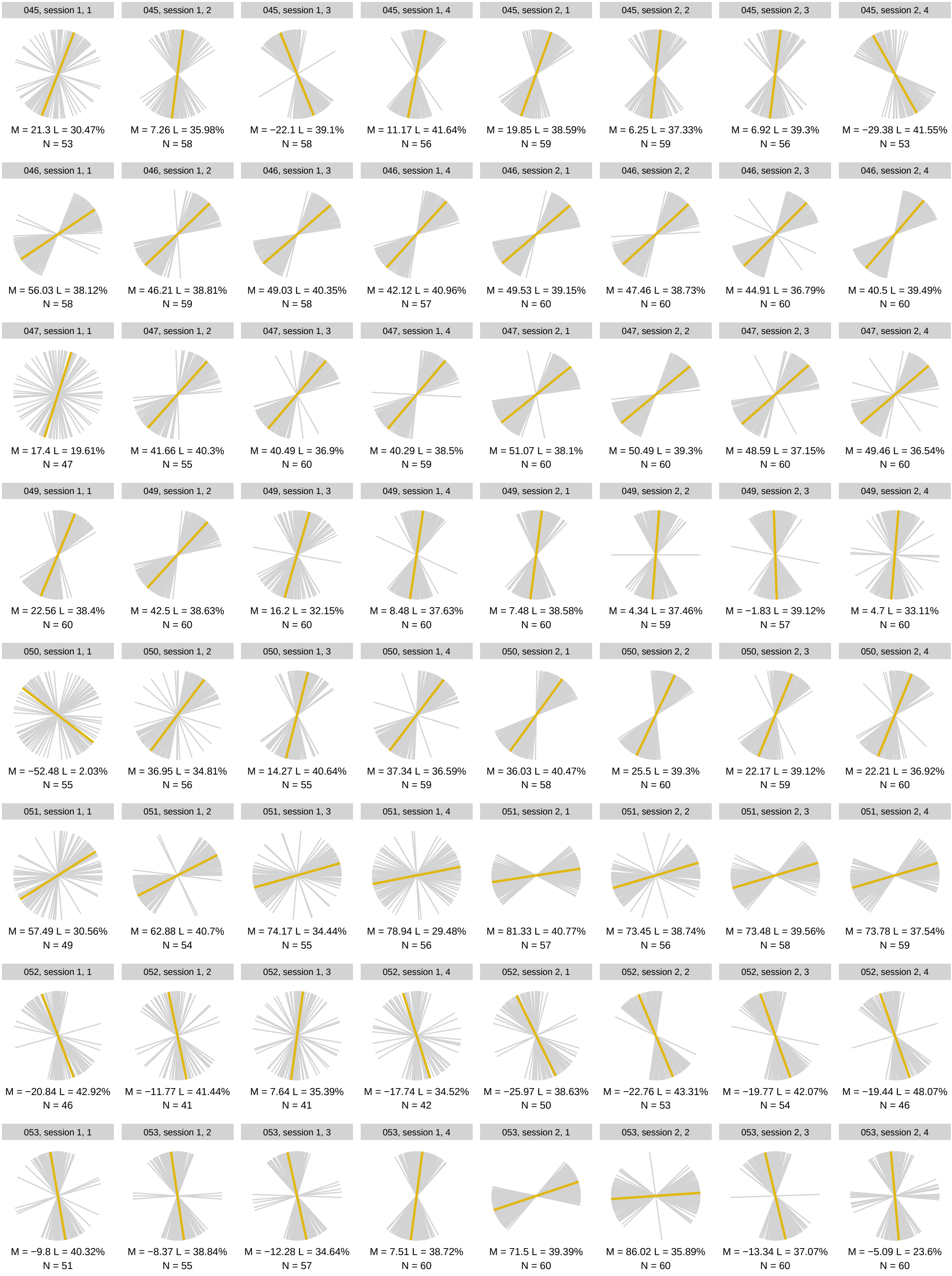

Figure A17. Visual representation of the absolute orientation bias of an individual per block of 60 trials, corrected for implausible 90° responses, separately for the first and the second session (Part 6/10). The colored oriented line indicates the mean direction of the absolute orientation bias per individual per block. Also the numeric values for the mean direction (M) and the magnitude (L) of the absolute orientation bias and the number of included trials (N) per individual per block are given.

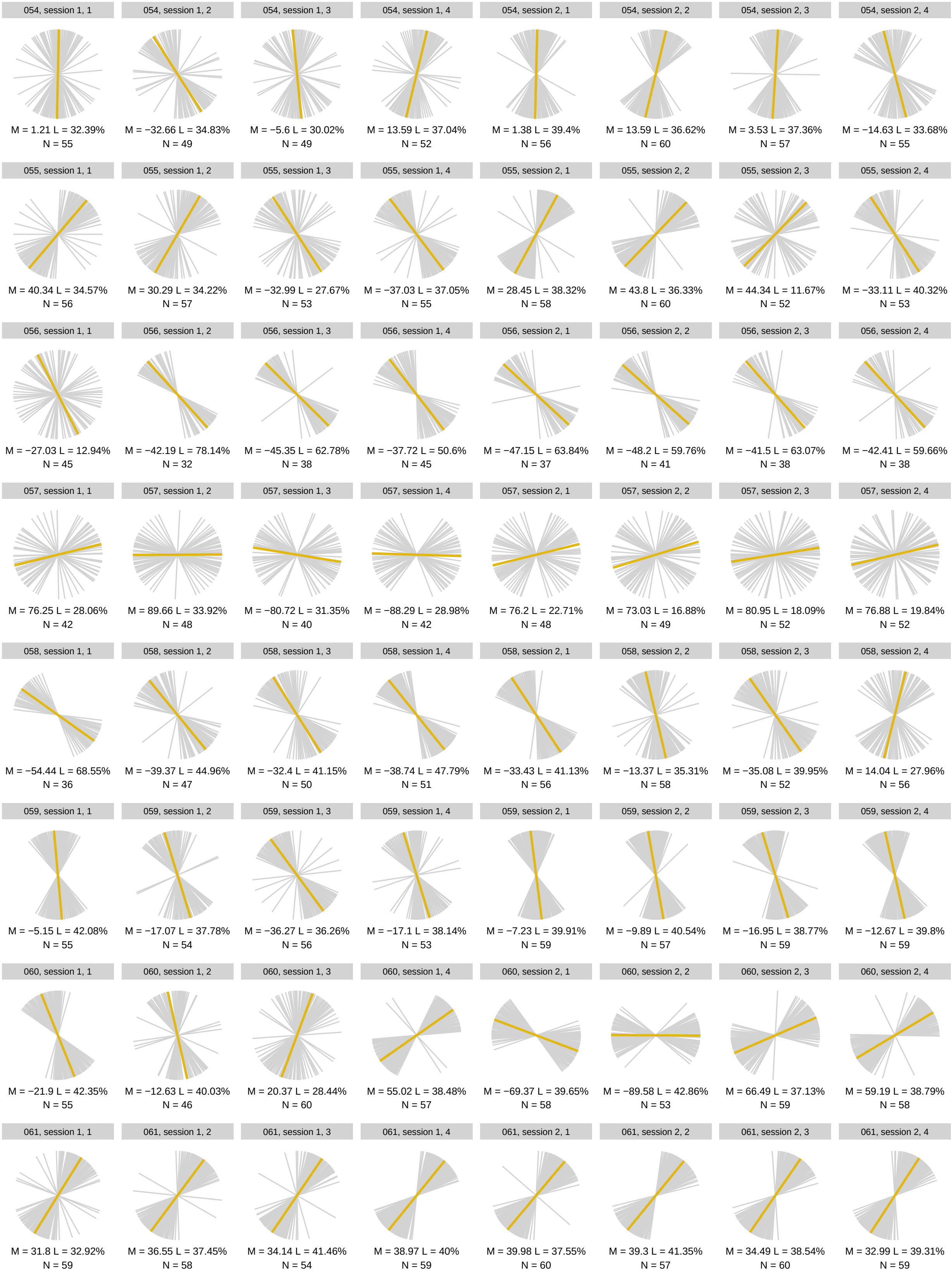

Figure A18. Visual representation of the absolute orientation bias of an individual per block of 60 trials, corrected for implausible 90° responses, separately for the first and the second session (Part 7/10). The colored oriented line indicates the mean direction of the absolute orientation bias per individual per block. Also the numeric values for the mean direction (M) and the magnitude (L) of the absolute orientation bias and the number of included trials (N) per individual per block are given.

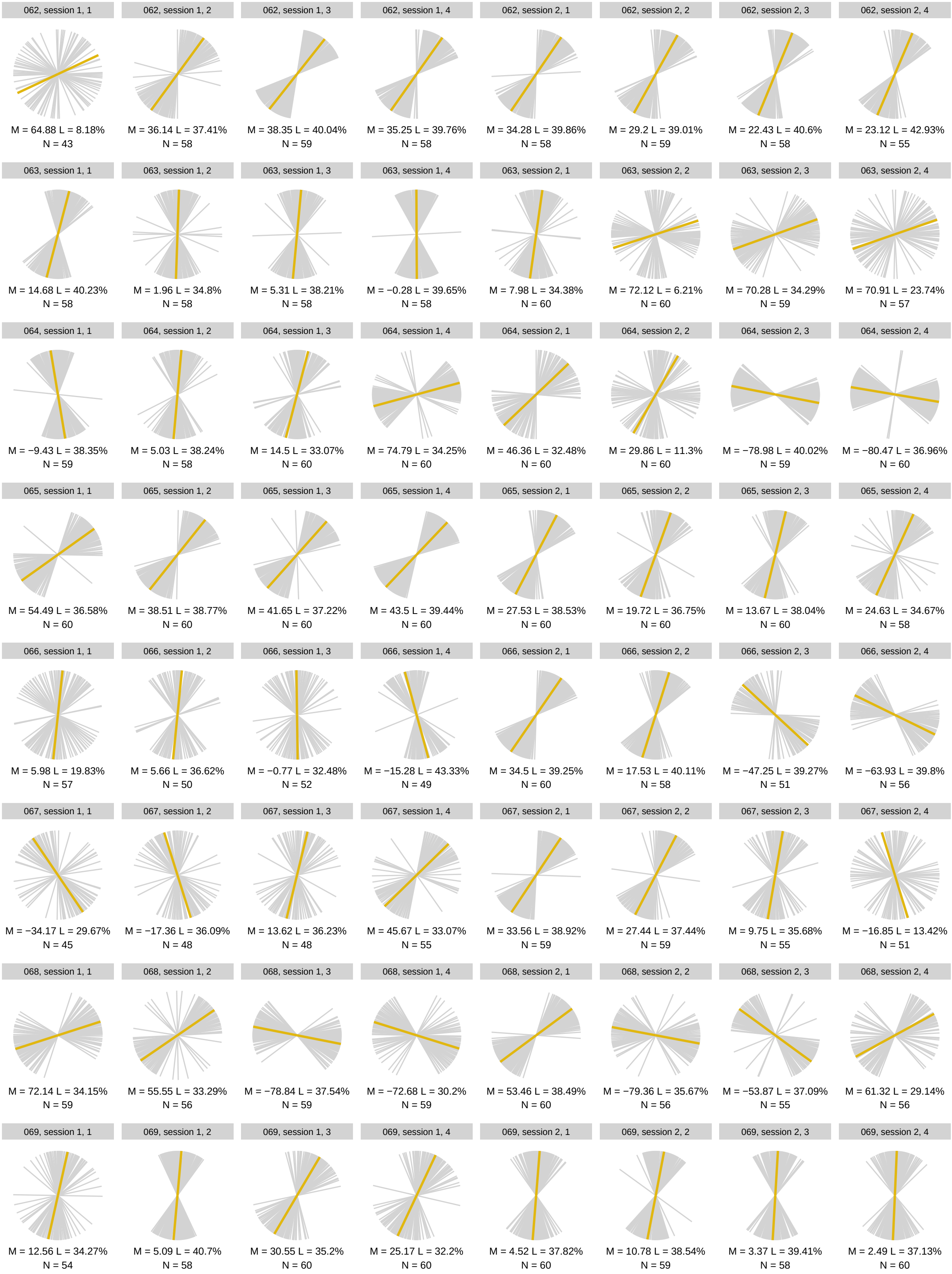

Figure A19. Visual representation of the absolute orientation bias of an individual per block of 60 trials, corrected for implausible 90° responses, separately for the first and the second session (Part 8/10). The colored oriented line indicates the mean direction of the absolute orientation bias per individual per block. Also the numeric values for the mean direction (M) and the magnitude (L) of the absolute orientation bias and the number of included trials (N) per individual per block are given.

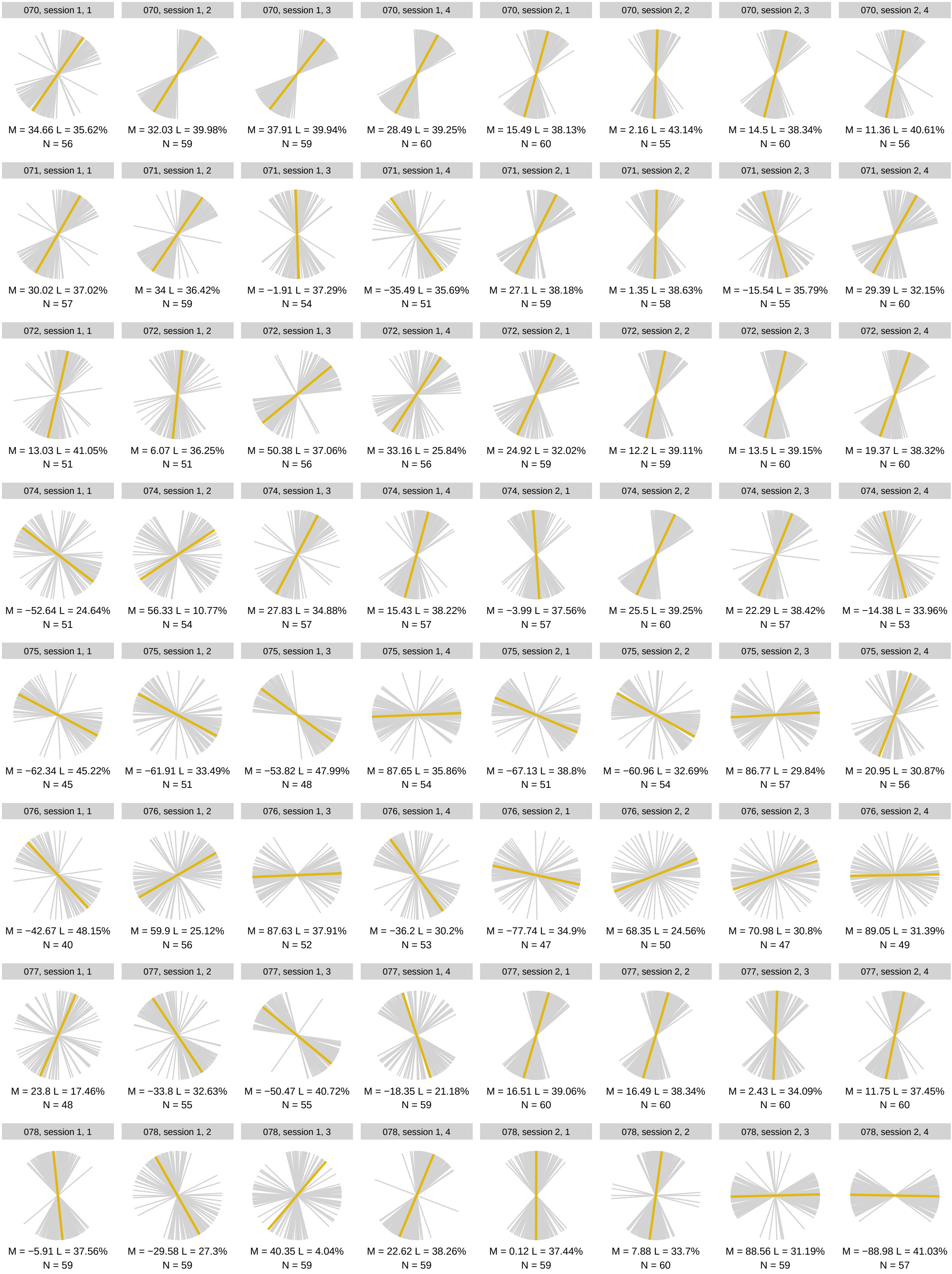

Figure A20. Visual representation of the absolute orientation bias of an individual per block of 60 trials, corrected for implausible 90° responses, separately for the first and the second session (Part 9/10). The colored oriented line indicates the mean direction of the absolute orientation bias per individual per block. Also the numeric values for the mean direction (M) and the magnitude (L) of the absolute orientation bias and the number of included trials (N) per individual per block are given.

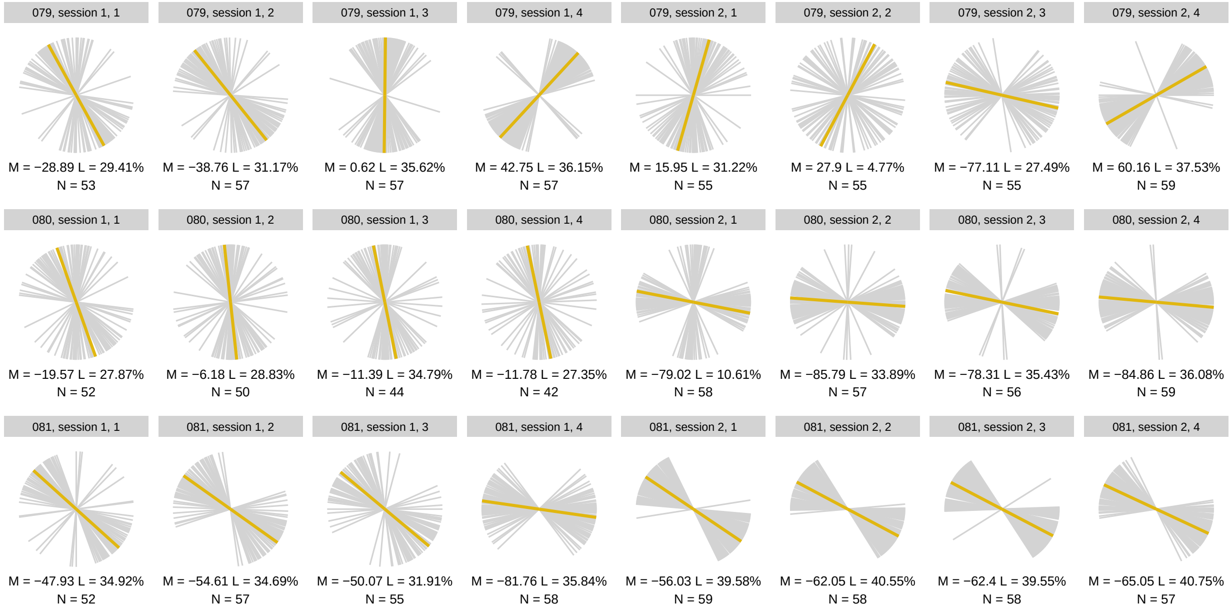

Figure A21. Visual representation of the absolute orientation bias of an individual per block of 60 trials, corrected for implausible 90° responses, separately for the first and the second session (Part 10/10). The colored oriented line indicates the mean direction of the absolute orientation bias per individual per block. Also the numeric values for the mean direction (M) and the magnitude (L) of the absolute orientation bias and the number of included trials (N) per individual per block are given.

References Appendix

Aust, F., & Barth, M. (2020). *papaja: Create APA manuscripts with R Markdown*. Retrieved from https://github.com/crsh/papaja

Bates, D., & Maechler, M. (2021). *Matrix: Sparse and dense matrix classes and methods*. Retrieved from https://CRAN.R-project.org/package=Matrix

Bürkner, P.-C. (2017). brms: An R package for Bayesian multilevel models using Stan. *Journal of Statistical Software*, *80*(1), 1–28. https://doi.org/10.18637/jss.v080.i01

Bürkner, P.-C. (2018). Advanced Bayesian multilevel modeling with the R package brms. *The R Journal*, *10*(1), 395–411.

Bürkner, P.-C. (2021). Bayesian item response modeling in R with brms and Stan. *Journal of Statistical Software*, *100*(5), 1–54. https://doi.org/10.18637/jss.v100.i05

Eddelbuettel, D., & Balamuta, J. J. (2018). Extending extitR with extitC++: A Brief Introduction to extitRcpp. *The American Statistician*, *72*(1), 28–36. https://doi.org/10.1080/00031305.2017.1375990

Eddelbuettel, D., & François, R. (2011). Rcpp: Seamless R and C++ integration. *Journal of Statistical Software*, *40*(8), 1–18. https://doi.org/10.18637/jss.v040.i08

Gepshtein, S., & Kubovy, M. (2005). Stability and change in perception: Spatial organization in temporal context. *Experimental Brain Research*, *160*(4), 487–495. https://doi.org/10.1007/s00221-004-2038-3

Grolemund, G., & Wickham, H. (2011). Dates and times made easy with lubridate. *Journal of Statistical Software*, *40*(3), 1–25. Retrieved from https://www.jstatsoft.org/v40/i03/

Henry, L., & Wickham, H. (2020). *Purrr: Functional programming tools*. Retrieved from https://CRAN.R-project.org/package=purrr

Henry, L., Wickham, H., & Chang, W. (2020). *Ggstance: Horizontal 'ggplot2' components*. Retrieved from https://CRAN.R-project.org/package=ggstance

Hester, J., & Bryan, J. (2021). *Glue: Interpreted string literals*. Retrieved from https://CRAN.R-project.org/package=glue

Kay, M. (2021). *tidybayes: Tidy data and geoms for Bayesian models*. https://doi.org/10.5281/zenodo.1308151

Morey, R. D., & Rouder, J. N. (2018). *BayesFactor: Computation of bayes factors for common designs*. Retrieved from https://CRAN.R-project.org/package=BayesFactor

Murdoch, D., & Chow, E. D. (2020). *Ellipse: Functions for drawing ellipses and ellipse-like confidence regions*. Retrieved from https://CRAN.R-project.org/package=ellipse

Müller, K. (2020). *Here: A simpler way to find your files*. Retrieved from https://CRAN.R-project.org/package=here

Müller, K., & Wickham, H. (2021). *Tibble: Simple data frames*. Retrieved from https://CRAN.R-project.org/package=tibble

Pedersen, T. L. (2020). *Patchwork: The composer of plots*. Retrieved from https://CRAN.R-project.org/package=patchwork

Pedersen, T. L. (2021). *Ggforce: Accelerating 'ggplot2'*. Retrieved from https://CRAN.R-project.org/package=ggforce

Plummer, M., Best, N., Cowles, K., & Vines, K. (2006). CODA: Convergence diagnosis and output analysis for MCMC. *R News*, *6*(1), 7–11. Retrieved from https://journal.r-project.org/archive/

R Core Team. (2021). *R: A language and environment for statistical computing*. Vienna, Austria: R Foundation for Statistical Computing. Retrieved from https://www.R-project.org/

Schwiedrzik, C. M., Ruff, C. C., Lazar, A., Leitner, F. C., Singer, W., & Melloni, L. (2014). Untangling perceptual memory: Hysteresis and adaptation map into separate cortical networks. *Cerebral Cortex*, *24*(5), 1152–1164. https://doi.org/10.1093/cercor/bhs396

Stan Development Team. (2020). StanHeaders: Headers for the R interface to Stan. Retrieved from https://mc-stan.org/

Stan Development Team. (2021). RStan: The R interface to Stan. Retrieved from https://mc-stan.org/

Wickham, H. (2016). *ggplot2: Elegant graphics for data analysis*. Springer-Verlag New York. Retrieved from https://ggplot2.tidyverse.org

Wickham, H. (2019). *Stringr: Simple, consistent wrappers for common string operations*. Retrieved from https://CRAN.R-project.org/package=stringr

Wickham, H. (2021a). *Forcats: Tools for working with categorical variables (factors)*. Retrieved from https://CRAN.R-project.org/package=forcats

Wickham, H. (2021b). *Tidyr: Tidy messy data*. Retrieved from https://CRAN.R-project.org/package=tidyr

Wickham, H., Averick, M., Bryan, J., Chang, W., McGowan, L. D., François, R., … Yutani, H. (2019). Welcome to the tidyverse. *Journal of Open Source Software*, *4*(43), 1686. https://doi.org/10.21105/joss.01686

Wickham, H., Bryan, J., & Barrett, M. (2021). *Usethis: Automate package and project setup*. Retrieved from https://CRAN.R-project.org/package=usethis

Wickham, H., François, R., Henry, L., & Müller, K. (2021). *Dplyr: A grammar of data manipulation*. Retrieved from https://CRAN.R-project.org/package=dplyr

Wickham, H., Hester, J., & Bryan, J. (2021). *Readr: Read rectangular text data*. Retrieved from https://CRAN.R-project.org/package=readr

Wickham, H., Hester, J., Chang, W., & Bryan, J. (2021). *Devtools: Tools to make developing r packages easier*. Retrieved from https://CRAN.R-project.org/package=devtools

Wilke, C. O. (2020). *Cowplot: Streamlined plot theme and plot annotations for 'ggplot2'*. Retrieved from https://CRAN.R-project.org/package=cowplot

Xie, Y. (2015). *Dynamic documents with R and knitr* (2nd ed.). Boca Raton, Florida: Chapman; Hall/CRC. Retrieved from https://yihui.org/knitr/

Zhu, H. (2021). *kableExtra: Construct complex table with 'kable' and pipe syntax*. Retrieved from https://CRAN.R-project.org/package=kableExtra
